# Enhancing Oxygenic Photosynthesis by Cross-Linked Perylenebisimide “Quantasomes”

**DOI:** 10.1021/jacs.2c05857

**Published:** 2022-07-26

**Authors:** Thomas Gobbato, Francesco Rigodanza, Elisabetta Benazzi, Paolo Costa, Marina Garrido, Andrea Sartorel, Maurizio Prato, Marcella Bonchio

**Affiliations:** † Department of Chemical and Pharmaceutical Sciences, University of Trieste, Via L. Giorgieri 1, I-34127 Trieste, Italy; ‡ Department of Chemical Sciences, University of Padova, 35131 Padova, Italy; § Department of Chemical Sciences, INSTM UdR, Padova, University of Padova, 35131 Padova, Italy; ∥ Department of Chemical and Pharmaceutical Sciences, CENMAT, Center of Excellence for Nanostructured Materials, INSTM UdR, Trieste, University of Trieste, 34127 Trieste, Italy; ⊥ Center for Cooperative Research in Biomaterials (CIC biomaGUNE), Basque Research and Technology Alliance (BRTA), 20014 Donostia San Sebastián, Spain; # Basque Fdn Sci, Ikerbasque, 48013 Bilbao, Spain; ∇ Istituto per la Tecnologia delle Membrane, ITM-CNR, UoS di Padova, 35131 Padova, Italy

## Abstract

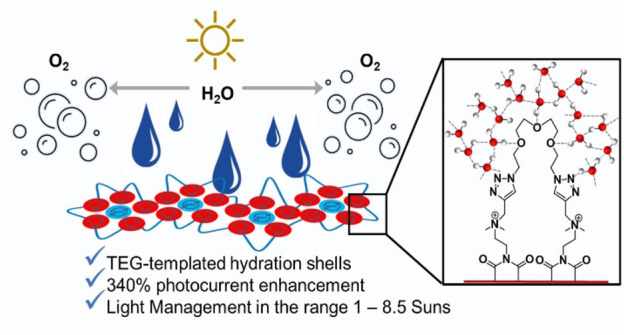

As the natural-born photoelectrolyzer for oxygen delivery, photosystem II (PSII) is hardly replicated with man-made constructs. However, building on the “quantasome” hypothesis (Science1964, 144, 1009−10111781160710.1126/science.144.3621.1009), PSII mimicry can be pared down to essentials by shaping a photocatalytic ensemble (from the Greek term ”soma” = body) where visible-light quanta trigger water oxidation. PSII-inspired quantasomes (QS) readily self-assemble into hierarchical photosynthetic nanostacks, made of bis-cationic perylenebisimides (PBI^2+^) as chromophores and deca-anionic tetraruthenate polyoxometalates (Ru_4_POM) as water oxidation catalysts (Nat. Chem.2019, 11, 146−1533051021610.1038/s41557-018-0172-y). A combined supramolecular and click-chemistry strategy is used herein to interlock the PBI-QS with tetraethylene glycol (TEG) cross-linkers, yielding QS-TEG_lock_ with increased water solvation, controlled growth, and up to a 340% enhancement of the oxygenic photocurrent compared to the first generation QS, as probed on 3D-inverse opal indium tin oxide electrodes at 8.5 sun irradiance (λ > 450 nm, 1.28 V vs RHE applied bias, TOF_max_ = 0.096 ± 0.005 s^–1^, FE_O2_ > 95%). Action spectra, catalyst mass-activity, light-management, photoelectrochemical impedance spectroscopy (PEIS) together with Raman mapping of TEG-templated hydration shells point to a key role of the cross-linked PBI/Ru_4_POM nanoarrays, where the interplay of hydrophilic/hydrophobic domains is reminiscent of PSII-rich natural thylakoids.

Photosystem II (PSII) organization in natural thylakoids suggests a perfectioned model to elaborate on the oxidative artificial photoelectrolyzers.^1−3^ Inspired by the PSII core-assembly, we have designed supramolecular “quantasomes” (QS), i.e., multichromophore architectures integrated with catalytic cores, which can convert radiation quanta into chemical energy by mimicking the PSII oxygenic function. This approach represents a paradigm change with respect to the classical sensitizer-catalyst dyad approach.^1,4^ Supramolecular **QSs** are readily obtained in water by encapsulation of the polyanionic oxygen evolving catalyst **Ru**_**4**_**POM**,^5−8^ within a multichromophore “corolla” of cationic perylenebisimides, **PBI**^**2+**^ (Scheme 1).^4^ The self-assembled **QSs** display a 5:1 stoichiometry ([**PBI**^**2+**^]_5_**Ru**_**4**_**POM**), dictated by complementary electrostatic interactions, and aggregate into a multilamellar architecture as a consequence of the PBI π–π aromatic stacking.^4^ In native chloroplasts, PSII paired function is regulated by protein–protein interactions, holding together the membrane stacks, while favoring the PSII contact (velcro effect, Scheme 1E).^9,10^ This asset is essential for the stability of the photosynthetic machinery and for its unique adaptation to illumination conditions.^11^ Inspired by the PSII membrane packing and building on our artificial design, we have now cross-linked the quantasome network by installing hydrophilic tetraethylene glycol (TEG) bridges, using click-chemistry (**QS-TEG**_**lock**_, Scheme 1A–D).^12^ Our results compare and contrast the photoelectrocatalytic (PEC) performance of **QS** versus **QS-TEG**_**lock**_, probed on the 3D-photoconductive lattice of inverse opal indium tin oxide (IO-ITO) electrodes (Scheme 1F,G).^13,14^ The locked structure exhibits up to 340% photocurrent increase associated with quantitative oxygen evolution (faradaic efficiency, FE_O2_ > 95%) and provides a keen stability gain under high solar irradiance (> 8 suns), compared to state-of-the-art PSII biohybrid and molecular photoanodes (Table S1).

**Scheme 1:**
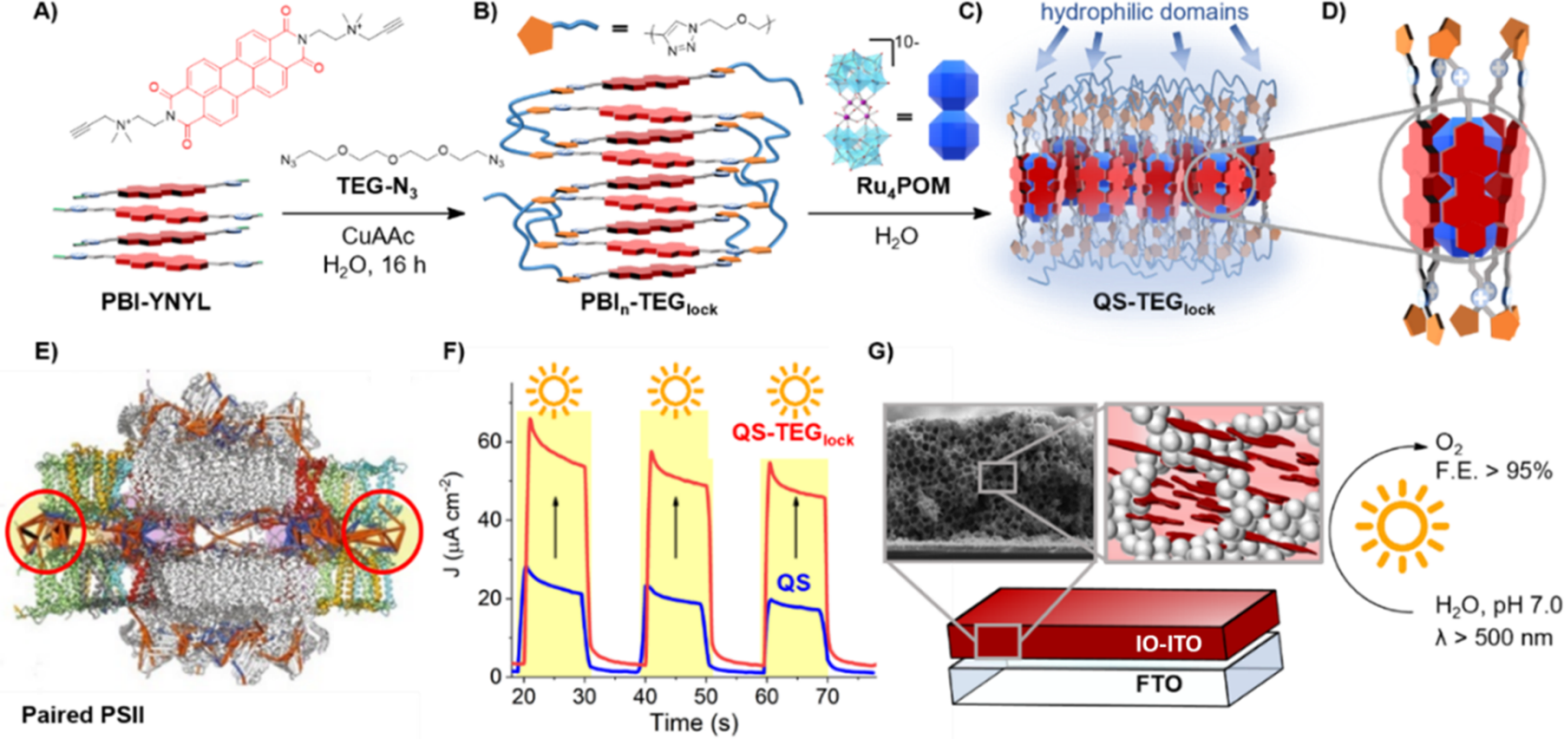
(A**)** Cross-linking of **PBI-YNYL** with bis-azido-TEG linkers yielding (B) **PBI**_**n**_**-TEG**_**lock**_ in water. (C) Self-assembly of **QS-TEG**_**lock**_. (D) Cartoon of the **QS-TEG**_**lock**_ photosynthetic unit. (E) Natural PSII pairs in appressed thylakoids.^10^ (F, G) Oxygenic PEC transients by **QS-TEG**_**lock**_ vs **QS** probed on IO-ITO electrodes.

**QS-TEG**_**lock**_ was obtained after installation of clickable 2-propynyl terminals on the PBI scaffold (**PBI-YNYL**) followed by copper-catalyzed azide–alkyne cycloaddition (CuAAC) in water, with bis-azido-TEG linkers (Scheme 1A).^12^ This protocol interlocks a multi-PBI network (**PBI**_**n**_**-TEG**_**lock**_, Scheme 1B) that can be purified by gel permeation chromatography.^12^ Characterization by FTIR and NMR spectroscopy confirms the formation of the expected triazole cross-linkers (Figures S1–S3). UV–vis spectra, in both DMF and H_2_O (pH 7), show a solvent-independent broad absorption spanning a 400–650 nm wavelength range (Figure S4), ruling out residual PBI monomers, typically characterized by sharp vibronic features.^15^ Indeed, ^1^H-diffusion ordered spectroscopy (DOSY) NMR recorded for **PBI**_**n**_**-TEG**_**lock**_ (2 mM in D_2_O, 400 MHz NMR, at 20 °C, Figure S5) provides a translational diffusion coefficient of 6.3 × 10^–11^ m^2^ s^–1^ (log *D*/m^2^ s^–1^ = −10.2), 1 order of magnitude lower than what reported for PBI monomers and dimers (in the range (2.0–9.0) × 10^–10^ m^2^ s^–1^, Table S2) and consistent with a columnar stacking of ∼20 PBI cores (*d* = 6.7 nm, based on a hydrodynamic spherical model; see Table S2).^16,17^ Accordingly, **PBI**_**n**_**-TEG**_**lock**_ shows aggregation-induced emission quenching in DMF with a fluorescence quantum yield of 4% (fluorescence lifetime τ_Fl,1_ = 1.3 ns, τ_Fl,2_ = 4.0 ns, Figures S6 and S7), while maintaining an estimated potential of the excited state, *E*(**PBI**_**n**_**-TEG**_lock_^*/•**–**^) **=** 2.26 V vs NHE, suitable to drive photoassisted water oxidation (Figure S8).^4^ Association of **PBI**_**n**_**-TEG**_**lock**_ with **Ru**_**4**_**POM** yields the integrated **QS-TEG**_**lock**_, as probed by UV–vis, fluorescence, and ζ-potential titrations (25–100 μM, pH 7, Figure S9). The resulting spectral fingerprint is typical of the quantasome assembly (Figure 1A), which suggests a similar structural motif, where the multi-PBI network is templated around the polyoxometalate.^4^ This is confirmed by powder X-ray diffraction (PXRD) patterns that are consistent with previous WAXS/SAXS evidence,^4^ ascribed to a lamellar-type structure, with broad π–π stacking reflections at *q*_c_ = 18–21 nm^–1^ and low q reflections at q_b_ = 4.7–4.9 nm^–1^ arising from the **Ru**_**4**_**POM** scattering centers. However, the interlamellar diffraction peak at *q*_a_ = 3.2 nm^–1^ is not observed for **QS-TEG**_**lock**_, likely due to a dislocation of the lamellar stacks by the TEG spacers (Figure 1B). Colloid characterization by dynamic light scattering (DLS, 25 μM in H_2_O) indicates smaller dimensions for **QS-TEG**_**lock**_ compared to **QS**, with hydrodynamic radius distribution centered respectively at 20 and 95 nm (Figure 1C), which supports the **QS-TEG**_**lock**_ improved stability against overaggregation and precipitation (> 10 h, up to 5 mM in water, Figure S10). **QS-TEG**_**lock**_ exhibits a superior PEC response, probed upon co-deposition of the quantasome building blocks on IO-ITO electrodes (600 ± 100 nm voids, 10 ± 2 μm film thickness, roughness factor RF = 950 ± 200, Figures S11 and S12, Table S3). Deep infiltration of the IO-ITO cross-section is demonstrated by the EDX-SEM profile of the Ru component (Figure 1D). In all cases, diffuse reflectance spectra of the IO-ITO|**QS-TEG**_**lock**_ vs **QS** photoanodes display a superimposable spectral envelope matching the quantasome signature (Figure 1A, solid lines).^18^ Chopped light linear sweep voltammetries (LSV) of IO-ITO|**QS-TEG**_**lock**_ vs **QS** electrodes (12 nmol cm^–2^, Figure 2A) display a photocurrent onset at 0.62 V vs RHE and a dual transient regime with dominant recombination at low applied bias (<0.90 V vs RHE) while reaching an optimal charge collection in the range 0.90–1.62 V vs RHE. Photocurrent densities up to *J*(**QS-TEG**_**lock**_) = 100 ± 10 μA cm^–2^ and *J*(**QS**)= 35 ± 3 μA cm^–2^ are recorded at 1.12 V vs RHE, anticipating the thermodynamic barrier for oxygen evolution (*E*(O_2_/H_2_O) = 1.23 V vs RHE) and reaching values of *J*(**QS-TEG**_**lock**_) = 370 ± 30 μA cm^–2^ and *J*(**QS**) = 290 ± 40 μA cm^–2^ at 1.52 V vs RHE (Figure 2A), in both cases leading to quantitative oxygen evolution (FE_O2_ > 95%) as determined with generator-collector method (Figures S15 and S16, Table S4). The photocurrent density and associated turnover frequency (TOF) depend on the applied voltage, showing a well-behaved, log(TOF) vs *E* (V), steady increase for **QS-TEG**_**lock**_ while a biphasic plot is obtained for **QS** with a break point at 1.28 V vs RHE (Figure 2B). At this break potential, the *J*(**QS-TEG**_**lock**_)/*J*(**QS**) photocurrent enhancement reaches a 340% apex of a volcano-type profile (Figure 2C). This potential-dependent regime is indicative of a rate-limiting interfacial charge-transfer at the WOC sites triggering diverse mechanistic manifolds (Scheme S1).^8,19^ Photoelectrochemical impedance spectroscopy (PEIS) allows us to deconvolute the photogenerated charge-transfer resistance (*R*_CT_) at the quantasome/water interface, as a function of the applied potential (Figure 2C, Figures S17–S19, Table S5). It turns out that the hydrophilic domains of **QS-TEG**_**lock**_ are instrumental to lower *R*_CT_, boosting the oxygenic performance at levels that can be matched by **QS** only upon increasing the applied bias, to counteract competitive recombination phenomena.^20^ The incident photon to current efficiency (IPCE, Figure 2D) profile confirms the competent action spectrum of the PBI-quantasomes (λ = 470–500 nm) and is consistent with the efficiency enhancement imparted by the **QS-TEG**_**lock**_ structural modification (IPCE up to 1.2% at 1.52 V vs RHE applied bias, Figure S20, Table S6).^21^ Chopped light chronoamperometry (CLCA) experiments, recorded at increasing quantasome loading (2.4–12.0 nmol cm^–2^, Figure 2E), remark the **QS-TEG**_**lock**_ advantage, which provides a linear photocurrent increase in the entire loading range, paralleling the photoanode optical density (Figure 2E, gray squares). On the contrary, a steady deviation is observed at **QS** loading of > 5.0 nmol cm^–2^, leveling the photocurrent to a lower plateau, likely due to a major colloid clustering (Figures 2E and S21, Tables S7 and S8).^22^ Under light management conditions, probed at photon irradiance in the range *I*_ph_ = 100–850 mW cm^–2^ (1–8.5 suns), both PBI-quantasomes reach a similar saturation plateau at *I*_ph_ > 6.7 suns, which sets the upper limit for a photon flux-regulated photocurrent (yellow area in Figure 2F, Figure S23). Above this threshold, the main photocurrent effector is independent of the light intensity (blue area in Figure 2F, Tables S11 and S12).^23,24^ To decouple the hydrophilic effect of TEG terminals from their cross-linking impact, the **PBI-YNYL** cores were clicked with methoxy-terminated, monoazide TEG pendants,^12^ preventing their covalent interlocking (Figure S26). This yields the unlocked TEGylated quantasome by self-assembly with **Ru**_**4**_**POM** (Figure S26). The resulting **QS-TEG**_**unlock**_ features the expected spectral signature, similar CLCA response, and quantitative oxygen production (Figures S27–S29, Tables S15 and S16, FE_O2_ > 95%), confirming that decoration of the PBI scaffold with TEG residues, with or without cross-linking, can leverage the quantasome hydration and facilitate water oxidation, under light-assisted or dark electrocatalytic conditions (Figures S30–S32 and Tables S17–S19).^25,26^ Formation of TEG-templated hydration shells has been detected by Raman microscopy of water exposed photoanodes (Figure 3, Supporting Information section 1.2),^27^ showing a diffuse water distribution (band area 3100–3600 cm^–1^) and a specific effect of the TEG residues in structuring “ordered water” via H-bonding, (band area < 3350 cm^–1^, green and red traces in the blue zone Figure 3A, Figures S33–S35) with respect to “disordered (bulk) water” observed for the TEG-free **QS** (band area >3350 cm^–1^, blue trace in the pink zone, Figure 3A).^27^ The added value of TEG cross-linkers stems from the improved **QS-TEG**_**lock**_ film stability under a 1 h photoelectrolysis at > 8 sun irradiance, whereby the unlocked structure reports a major photocurrent loss (73% vs 56% Figure S36, Table S1).^28^ Our results highlight that modulation of the molecular environment is strategic to boost the photosystem performance. Following this approach, our vision aims at a modular, biomorphic design of fully functional integrated photosynthetic architectures to face the artificial photosynthesis challenge.

**Figure 1:**
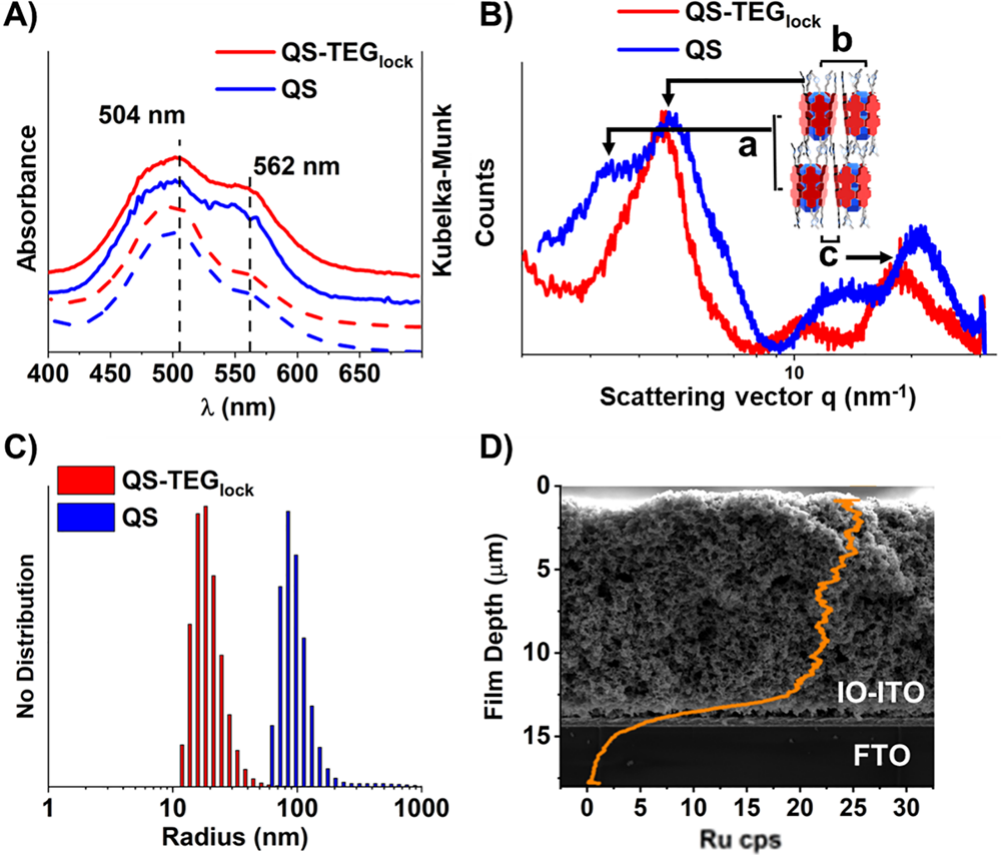
**QS-TEG**_**lock**_ versus **QS** characterization. (A) Superimposed UV–vis spectra, shifted for clarity, in H_2_O (dashed lines) and diffuse reflectance spectra of IO-ITO electrodes (solid lines, KM units). (B) PXRD patterns with notable distances (see text). (C) DLS size distribution in H_2_O. (D) SEM-EDX cross-section of loaded IO-ITO electrodes mapping Ru infiltration (2.4 nmol cm^–2^ loading, orange line).

**Figure 2:**
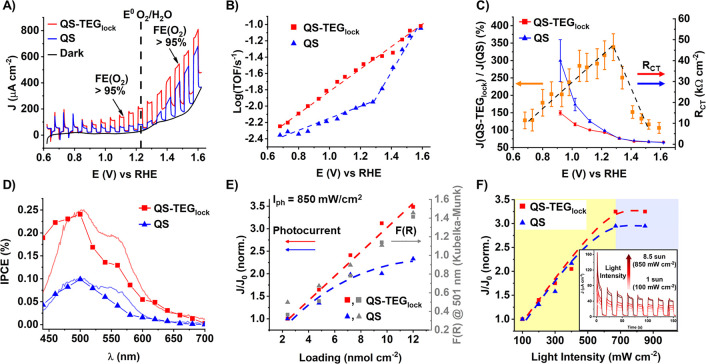
(A) Representative chopped light LSV (solar simulator AM 1.5 G, 850 mW cm^–2^ = 8.5 suns, λ > 450 nm, scan rate 10 mV/s, in 0.1 M NaHCO_3_, pH 7) of IO-ITO|**QS-TEG**_**lock**_ (red trace) vs **QS** (blue trace) and (B) corresponding log(TOF) vs *E* (V) (mean values ± 15%) based on nominal loading (12 nmol cm^–2^). (C) Photocurrent enhancement (*J*(**QS-TEG**_**lock**_)/*J*(**QS**)%, left axis, orange squares) and related *R*_CT_ values resulting from PEIS measurements at increasing applied potential (right axis). (D) Corresponding action spectra (mean values ± 10% at 1.12 V RHE applied bias, Supporting Information section 1.3) with superimposed diffuse reflectance spectra. Normalized *J*/*J*_o_ plots (scaled with respect to the minimum value, *J*_o_, mean values ± 15% at 1.12 V RHE applied bias) for IO-ITO|**QS-TEG**_**lock**_ (red squares) vs **QS** (blue triangles) at (E) increasing loading (2.4–12.0 nmol cm^–2^) with corresponding diffuse reflectance intensity converted in KM units F(R) values at 500 nm (gray triangles and squares) and at (F) increasing light irradiance (*I*_ph_ = 100–850 mW cm^–2^). Representative CLCA transients (inset) of IO-ITO|**QS-TEG**_**lock**_.

**Figure 3:**
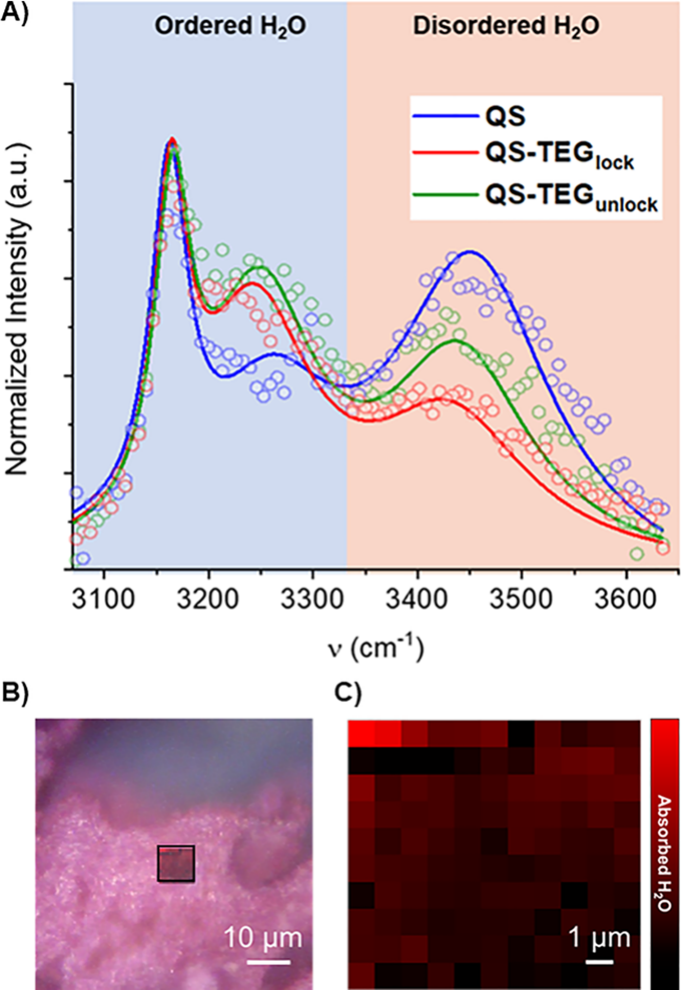
Raman microscopy mapping of quantasome hydration shells on loaded IO-ITO electrodes. (A) Spectra deconvolution by fitting to Gaussian peaks (solid lines) corresponding to tetrahedrally ordered water (< 3350 cm^–1^) and disordered water (> 3350 cm^–1^). (B) Optical microscopy image of ITO-IO|**QS-TEG**_**lock**_; red square section: mapped surface in (C) where the integrated area of the O–H stretching signal (3100–3600 cm^–1^) is plotted for every pixel.

## Supporting Information Available

Instruments and methods, synthetic and characterization procedures, photoelectrocatalytic experiments, data analysis, and benchmarking tables (PDF)
