# Supplementary material for: Enhancing Oxygenic Photosynthesis by Cross-Linked Perylenebisimide “Quantasomes”
Source: J Am Chem Soc. 2022 Jul 26;144(31):14021–5. doi: 10.1021/jacs.2c05857 (PMC9376926; doi:10.1021/jacs.2c05857)
Supplement: Supplementary file 1 — ja2c05857_si_001.pdf [file ja2c05857_si_001.pdf]

Supporting Information for

Enhancing Oxygenic Photosynthesis by Cross-Linked Perylenebisimide "Quantasomes"

Thomas Gobbato, Francesco Rigodanza, Elisabetta Benazzi, Paolo Costa, Marina Garrido, Andrea Sartorel, Maurizio Prato and Marcella Bonchio

Department of Chemical Sciences, INSTM UdR, Padova, University of Padova, 35131 Padova, Italy; Istituto per la Tecnologia delle Membrane, ITM-CNR, UoS di Padova, 35131 Padova, Italy; orcid.org/0000- 0002-7445-0296.
Email: marcella.bonchio@unipd.it
Department of Chemical and Pharmaceutical Sciences, CENMAT, Center of Excellence for Nanostructured Materials, INSTM UdR, Trieste, University of Trieste, 34127 Trieste, Italy; Center for Cooperative Research in Biomaterials (CIC biomaGUNE), Basque Research and Technology Alliance (BRTA), 20014 Donostia San Sebastián, Spain; Basque Fdn Sci, Ikerbasque, 48013 Bilbao, Spain; orcid.org/0000-0002-8869-8612
E-mail: prato@units.it

1. EXPERIMENTAL SECTION 3

1.1 MATERIALS 3
1.2 EQUIPMENT AND METHODS 3
1.3 PHOTOELECTROCHEMICAL EXPERIMENTS 4
1.4 DETERMINATION OF FARADAIC EFFICIENCY 6
1.5 PREPARATION OF IO-ITO 8
1.6 DETERMINATION OF IO-ITO ECSA (ELECTROCHEMICAL SURFACE AREA) BY DOUBLE-LAYER CAPACITANCE MEASUREMENTS 8
1.7 QUANTASOME DEPOSITION ON IO-ITO ELECTRODES 9
1.8 DETERMINATION OF SURFACE-ACTIVE SITES OF RU_4POM 9

2. SYNTHETIC PROCEDURE 10

2.1 SYNTHESIS OF PBI-YNYL[16] 10
2.2 SYNTHESIS OF TEG-OTS 11
2.3 SYNTHESIS OF TEG-N_3 11
2.4 SYNTHESIS OF PBI_N-TEG_{LOCK} 12
2.5 SYNTHESIS OF 2-(2-(2-METHOXYETHOXY)ETHOXY)ETHYL P-TOLUENESULFONATE 13
2.6 SYNTHESIS OF TEG-OME 13
2.7 SYNTHESIS OF PBI-TEG_{UNLOCK} 14

3. SUPPORTING FIGURES 15

3.1 Benchmarking of state-of-the-art photoanodes for water oxidation 15
3.2 Fourier Transform Infrared Spectroscopy 16
3.3 NMR characterization 17
3.4 Absorption Spectroscopy 17
3.5 DOSY NMR studies 18
3.6 Translational diffusion coefficient of PBIs 18
3.7 Fluorescence Quantum yield and decay dynamics 19
3.8 PBI_N-TEG_{LOCK} absorption and emission spectra and redox properties 20
3.9 Spectrophotometric and z -potential Titrations of the PBI_N-TEG_{LOCK} / Ru_4POM assembly 21
3.10 Stability of QS and QS-TEG_{LOCK} colloids 22
3.11 Scanning Electron Microscopy imaging of the IO-ITO electrodes 22
3.12 Determination of electrochemical surface area 23
3.13 Chopped light chronoamperometry (CLCA) at different PBI^{2+}/Ru_4POM and PBI_N-TEG_{LOCK}/Ru_4POM Ratio 24
3.14 Faradaic efficiency of Oxygen production (FE_{O2}) by IO-ITO|QS and IO-ITO|QS-TEG_{LOCK} determined with generator collector method 25
3.15 PEIS analysis for IO-ITO|QS and IO-ITO|QS-TEG_{LOCK} 26
3.16 IPCE and APCE of IO-ITO|QS and IO-ITO|QS-TEG_{LOCK} 28
3.17 Quantasome Loading 29
3.18 Light management 31
3.19 Synthesis and PEC performance of QS-TEG_{UNLOCK} 33
3.20 Determination of surface-active sites of Ru_4POM used for calculation of the dark electrocatalytic TOF 35
3.21 Dark electrocatalytic water oxidation on IO-ITO|QS, IO-ITO|QS-TEG_{LOCK} and IO-ITO|QS-TEG_{UNLOCK} 36
3.22 Raman Spectroscopy Mapping of Quantasomes Hydration Shells 38
3.23 Prolonged photoelectrochemical experiments 39
3.24 Stability of Photoanodes before and after oxygenic turnover 40

4. APPENDIX 43

4.1 Reproducibility of QS and QS-TEG_{LOCK} photoanodes 43

5. REFERENCES 44

1. Experimental section

1.1 Materials

Chemicals and solvents for all synthetic and characterization procedures have been purchased from Sigma-Aldrich and Acros and used as received if not differently specified. All solvents used for photochemical characterization of single molecules and hybrid materials are of spectroscopic grade (99.5%). Deuterated solvents have been purchased from Sigma-Aldrich and Cambridge Isotope Laboratories. ITO nanoparticles, Alconox and TEC 8 Fluorine-doped Tin Oxide (FTO) conductive glass slides were obtained from Sigma Aldrich. Polystyrene beads were purchased from Polyscience Inc. Milli-Q water was obtained using a Millipore apparatus, equipped with 0.22 µm filters. N,N′-Bis(2-(dimethylammonium)ethylene)perylene-3,4,9,10-tetracarboxylic acid bisimide (**PBI^{2+}**), N,N′-Bis(2-(trimethylammonium)ethylene)perylene-3,4,9,10-tetracarboxylic acid bisimide bisbromide salt (**PBI-YNYL**) and $Na_{10}[Ru_4(\mu\text{-}O)_4(\mu\text{-}OH)_2(H_2O)_4(\gamma\text{-}SiW_{10}O_{36})_2]$ (**Ru_4POM**) have been synthesized according to literature procedures.[1–3]

1.2 Equipment and Methods

NMR spectra were recorded on Bruker 400 Advance III HD equipped with a BBI-z grad probe head 5 mm and Bruker 300 equipped with a BBI-ATM-z grad probe head 5 mm. The chemical shifts (δ) for ^{1}H and ^{13}C are given in ppm relative to residual signals of the solvents ($CHCl_3$ @ 7.26 ppm ^{1}H NMR, 77.16 ppm ^{13}C NMR). Coupling constants are given in Hz. The following abbreviations are used to indicate the multiplicity: s, singlet; d, doublet; t, triplet; q, quartet; m, multiplet; bs, broad signal. Diffusion ordered spectroscopy (DOSY) NMR experiments were carried out with the sample at a concentration of 2 mM in D_2O to evaluate the aggregate size in solution. The value obtained for the translational diffusion coefficient is subsequently converted into the hydrodynamic radius applying the Stokes-Einstein equation $D = k_BT/(6\pi\eta R)$ (see Table S2).

Steady-state absorption spectroscopy studies have been performed at room temperature on a Varian Cary 5000 UV-Vis-NIR double beam spectrophotometer or on a Varian Cary 100 UV-Vis beam spectrophotometer. 10 mm path length Hellma Analytics 100 QS quartz cuvettes have been used.

Steady-state fluorescence spectra have been recorded on a Varian Cary Eclipse Fluorescence spectrophotometer or on a Horiba FluoroMax3 spectrophotometer; 10 mm path length Hellma Analytics 100F QS quartz cuvettes have been used.

Fluorescence decay dynamics studies have been performed using 405 nm laser pulses on a FLS1000 by Edinburgh Instruments equipped with a PMT-980 detector. 10 mm path length Hellma Analytics 100F QS quartz cuvettes have been used.

Fluoresce Quantum Yield Φ_f measurements were performed with Rhodamine 6G in ethanol (literature quantum yield 0.91 at 490 nm) as the standard.[4] The fluorescence quantum yields were calculated, exciting the samples at 480 nm, according to equation:

$$\Phi_{f,x} = \Phi_{f,st} \cdot \frac{F_x}{F_{st}} \cdot \frac{f_{st}}{f_x} \cdot \frac{n_x^2(\lambda_{em})}{n_{st}^2(\lambda_{em})}$$

F is the integral photon flux, *f* is the absorption factor, *n* is the refractive index of the solvent and Φ_f is the quantum yield. The index x denotes the sample, and the index st denotes the standard. The Φ_f reported in the work are averages of 5 measurements at different concentrations.

Cyclic Voltammetry (CV) characterization was carried out in acetonitrile (MeCN)/0.1 M tetrabutylammonium hexafluorophosphate ($TBAPF_6$) at room temperature, on an Epsilon LC electrochemical workstation (BASi research products) in a glass cell. A typical three-electrode cell was employed, which was composed of glassy carbon (GC) working electrode (3 mm diameter), a platinum wire as counter electrode and a saturated calomel electrode (SCE) as reference electrode (RE). Oxygen was removed by purging MeCN solution with high-purity Nitrogen. The potential of ferrocenium/ferrocene (Fc/Fc^+) couple was measured and found to be 0.45 V vs SCE, in agreement with the value reported in literature (in MeCN).[5] The GC electrode was polished before any measurement with diamond paste, ultrasonically rinsed with deionized water for 15 minutes and electrochemically activated in the background solution by means of several voltammetric cycles at 100 mV/s between the anodic and cathodic solvent/electrolyte discharges.

ζ-Potential measurements and Dynamic Light Scattering (DLS) have been performed using a Malvern Zetasizer Nano ZS90 instrument, in poly-(methyl methacrylate) (PMMA) cuvettes containing 1 ml of the solution under investigation.

Powder XRD measurements have been performed on a Bruker D8 diffractometer equipped with a Göbel mirror and a CuKα X-ray source.

SEM was performed with a Zeiss Sigma HD microscope, equipped with a Schottky FEG source, one detector for backscattered electrons and two detectors for secondary electrons (InLens and Everhart Thornley). The microscope is coupled to an EDX detector (from Oxford Instruments, x-act PentaFET Precision) for X-rays microanalysis, working in energy dispersive mode.

Spectral reflectance measurements were recorded with an integrating sphere (120 mm in diameter and BenFlect inner surface coating) fully integrated in the sample compartment of the Edinburgh photoluminescence spectrometer model FLS1000, equipped with double monochromators, a 450 W Xe arc lamp as the excitation source and an extended red photomultiplier (Hamamatsu R13456, spectral response 185 – 980 nm) as detector. For the reflectance spectra, the step and dwell time were set at 1 nm and 0.2 s, respectively, and the slit was kept at 2 nm and 0.2 nm for excitation and emission monochromators, respectively. Reflectance was expressed in Kubelka-Munk units, according to equation:

$$\frac{k}{s} = \frac{(1 - R_{\propto})^2}{2R_{\propto}}$$

Apparent Absorbance is calculated according to equation[6]:

$$A(\lambda) = -\ln(R)$$

Fourier-Transform Infrared (FT-IR) spectra have been recorded on a Varian 660 FT-IR with KBr pellets.

Raman measurements were acquired with a Renishaw instrument, model Invia reflex equipped with 532, 633, and 785 nm lasers. In particular, for the investigation of Quantasomes hydration shell, we employed 532 nm excitation with detecting window 3000-3700 cm^{-1}. The Raman spectra were registered after addition of 5 µL of milli-Q water to the IO-ITO|**QS, QS-TEG$_{lock}$** and **QS-TEG$_{unlock}$** electrodes prepared as described in Section 1.5. The samples were analyzed in three different surface spots and repeated in duplicate. The Raman mapping was performed selecting an area of 10 µm of the electrode that was consequently divided in 100 pixels, every pixel represents the integral area of the 3100-3700 $cm\text{-}^{1}$ signal ascribed to the -OH stretching modes.

1.3 Photoelectrochemical experiments

Electrochemical and photoelectrochemical experiments were carried out in sodium hydrogen carbonate $NaHCO_3$ 0.1 M, pH 7 (adjusted using HCl), on a PGSTAT302N potentiostat in a three-electrode configuration using as working electrode IO-ITO|**QS** or **QS-TEG$_{lock}$** and **QS-TEG$_{unlock}$** electrodes, as counter electrode Au and as reference electrode Ag/AgCl (NaCl 3 M). Potentials are then converted to RHE using the correlation

$V(RHE) = V(Ag/AgCl) + 0,205 + 0,0592 \cdot pH$. A LOT-QuantumDesign solar simulator, equipped with an AM 1.5 G filter, was used as the illumination source**.** The power of the light-source for light management experiments was measured using a THOR LABS PM100D power meter coupled with a CCD THOR LABS S370C and then to minimize the IO-ITO electrodes contribution, a 450 nm cut-off filter was used. Front illumination was adopted for all the experiments and unless otherwise stated, the majority of the measurements were recorded at 850 mW/cm^2 in order to better appreciate the photocurrent delivered by the photoanodes. Current-voltage curves were all recorded at a scan rate of 10 mV/s. Current-voltage and current-time curves under chopped illumination (10 s) were acquired by manually chopping the excitation source. All the photoelectrochemical data represent the average of three different samples. All the experiments were performed before on blank samples and then on co-deposited photoanodes. The electrodes were cleaned electrochemically scanning three times in dark CV (range 0.62 – 1.62 V vs RHE) until reaching a superimposable current response. This procedure enabled the elimination both of impurities coming from electrodes synthesis and of halides (i.e bromide) present in PBIs that could affect the measurements by behaving as redox mediators.[7] In particular, regarding IO-ITO|**QS-TEG$_{\mathbf{Lock}}$** and IO-ITO|**QS-TEG$_{\mathbf{unlock}}$**, EDX experiments after the electrochemical cleaning procedure confirmed the absence of Br signal (Scheme S1). Electrochemical analysis of voltammetric scans after cleaning procedures (spanning from 0.62 V to 1.62 V vs RHE) is reported in section 3.21 for IO-ITO|**QS**, or **QS-TEG$_{\mathbf{lock}}$** and **QS-TEG$_{\mathbf{unlock}}$** electrodes.

A) EDX experiments on IO-ITO|**QS-TEG$_{\mathbf{lock}}$** 12 nmol cm^{-2} after electrochemical cleaning procedure.

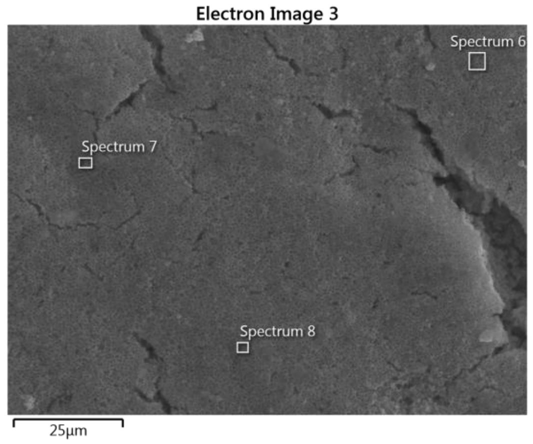

B) EDX experiments on IO-ITO|**QS-TEG$_{\mathbf{unlock}}$** 12 nmol cm^{-2} after electrochemical cleaning procedure.

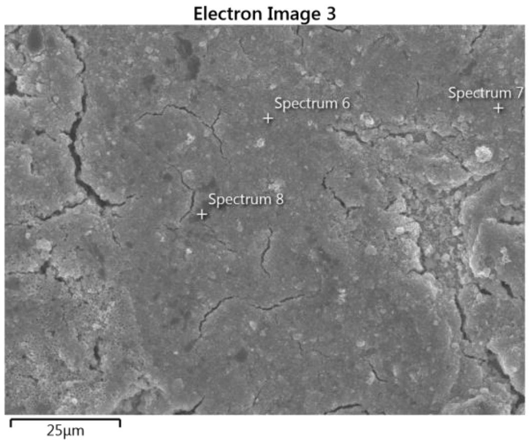

Spectrum Label	Spectrum 6	Spectrum 7	Spectrum 8
C	6.28	5.93	5.86
O	15.53	17.02	15.33
Na	0.09	0.11	0.07
**Br**	/	/	/
Ru	0.65	0.48	0.46
In	65.54	66.73	66.83
Sn	6.01	4.97	6.17
W	5.90	4.76	5.28
Total	100.00	100.00	100.00

Spectrum Label	Spectrum 6	Spectrum 7	Spectrum 8
C	4.26	4.40	5.80
O	14.03	18.90	20.46
Na	0.05	0.14	0.11
**Br**	/	/	/
Ru	0.17	0.20	0.50
In	72.28	67.69	63.45
Sn	7.20	6.22	5.41
W	2.01	2.45	4.27
Total	100.00	100.00	100.00

SEM-EDX experiments on co-deposited A) IO-ITO|**QS-TEG$_{\mathbf{lock}}$** and B) IO-ITO|**QS-TEG$_{\mathbf{unlock}}$** electrodes with 12 nmol cm^{-2} loading. "After electrochemical cleaning" samples are tested, confirming the absence of Br atom signal.

<u>Photoelectrochemical Impedance Spectroscopy (PEIS)</u> experiments were acquired with an Autolab PGSTAT302N potentiostat coupled with a FRA32M module and Nova electrochemical software. PEIS data were registered at different applied potentials (from 0.92 to 1.62 V vs RHE, every 0.10 V) using the experimental setup of photoelectrocehmical experiments and by applying a 10 mV amplitude perturbation with frequencies between 10^5 and 0.1 Hz. Data were fitted using Zview software (Scribner Associates).

For IPCE and APCE measurements the photocurrents and photon flux were determined at different wavelengths by dark/light chronoamperometry experiments, at 1.12 V and 1.52 V vs RHE, in sodium hydrogen carbonate $NaHCO_3$ 0.1 M, pH 7 (adjusted using HCl). Chronoamperometric experiments (100 s light/dark intervals) were performed under monochromatic irradiation in the range λ = 450 - 700 nm, with 10 nm steps, generated by a 175 W Xenon lamp coupled to an Applied Photophysics monochromator. The irradiance, i.e. the irradiated power per area, was calculated at each wavelength by a calibrated silicon photodiode. The IPCE value was calculated according to equation:

$$IPCE_\lambda = \frac{\Phi_{e^-}}{\Phi_{ph}} x\, 100 = \frac{J_\lambda\, x \frac{10^{-2}}{F}}{\frac{J_\lambda}{N_A}\, x\, E_\lambda} x100 \simeq 1240\, x\, \frac{J_\lambda}{\lambda\, x\, E_\lambda}$$

where Φ_{e-} and Φ_{ph} are the flux of electrons and incident photons (mol/s m^2), respectively, J_λ is the steady state photocurrent density (µA/cm^2) generated at λ (nm), F is the Faraday constant, I_λ is the irradiance (W m^{-2}), N_A is Avogadro's number and $E_\lambda = hc/\lambda \times 10^{-9}$ is the monochromatic photon energy (eV). The APCE value was then calculated from the IPCE data based on the absorption features of the photoanode, calculated as the light harvesting efficiency (LHE) at each wavelength, according to equation:

$$APCE_\lambda = \frac{IPCE_\lambda}{LHE_\lambda} = \frac{IPCE_\lambda}{1 - T(\lambda)} = \frac{IPCE_\lambda}{1 - 10^{-A(\lambda)}}$$

where T(λ) is the transmittance and A(λ) is the apparent absorbance calculated from reflectance at λ.

1.4 Determination of Faradaic Efficiency

In order to measure the O_2 evolved from the photoelectrochemical water oxidation by IO-ITO|**QS** or **QS-TEG$_{lock}$** and **QS-TEG$_{unlock}$**, the Generator–Collector method, originally introduced by the Mallouk group and subsequently adapted by Meyer, Finke and our group, was employed.[1,8–10] Briefly, the IO-ITO|**QS** or **QS-TEG$_{lock}$** and **QS-TEG$_{unlock}$** photoanode was illuminated under an applied bias, thus acting as O_2 generator. An FTO electrode (previously cleaned via 10 min sonication in KOH/iPrOH, 10 min sonication in iPrOH and annealed at 500 °C for 30 min) was sandwiched to the photoanode (with both conducting sides facing inward), in order to be used as the collector, i.e. the electrode at which the reduction of the evolved O_2 takes place. The sandwiched device was held together by 3 layers of unstretched parafilm (ca. 200 µm spacing), sealed together by employing a cell made with polyether ether ketone engineered by our group (see Scheme S2). The parafilm was designedly cut to form a U-shaped chamber to minimize O_2 loss, while still allowing the electrolyte access by capillary forces. Both the photoanode generator and the FTO collector were contacted using Cu tape (also covered by the parafilm layers), and respectively connected to the two working electrodes of a bipotentiostat (PGSTAT302N), while an Ag/AgCl and a Pt wire were used as the reference and the counter electrode respectively. The sandwich was then immersed in degassed $NaHCO_3$ 0.1 M solution (pH 7) and illuminated at 850 mW/cm^2 (using a calibrated LOT-QuantumDesign solar simulator, equipped with an AM 1.5 G filter plus a 450 nm cut off filter to minimize IO-ITO contribution). The generator electrode was held at 1.12 V and 1.52 V vs RHE bias, while the collector at -0.28 V vs RHE, which was identified to be the optimal O_2 reduction potential. In a typical experiment, currents at both the working electrodes were recorded for 100 s in the dark, then 100 s of illumination, then in the dark for additional 300 s, to allow the diffusion of oxidation products across the solution. The faradaic efficiency for O_2 production, ηO_2, can be calculated by:

$$\eta O_2 = \frac{Q_{coll}}{Q_{gen}} \times \frac{1}{\eta_{coll}}$$

where Q_{coll} is the integrated current measured at the Collector electrode, Q_{gen} is the integrated photocurrent measured at the Generator electrode, and η_{coll} is the collector efficiency (which must be quantified under the specific set-up and experimental conditions used). In particular, to the registered current values, we have subtracted the corresponding stable current values measured in the initial dark step. This value was usually negligible in the case of photoanodes, while on the FTO collector the reduction of some residual O_2 was registered. The corrected current values were then integrated (to yield Q_{gen} and Q_{coll}) using the "subtract baseline" tool in Origin, in order to account also for the current registered in the final dark step of the experiments. As regards η_{coll}, it has been estimated to be 78%, from generator-collector experiments registered on FTO-FTO sandwiches prepared in the same way of the analogous photoanode-FTO ones. Firstly, it was registered the dark current associated to water oxidation on FTO in $NaHCO_3$ 0.1 M solution (pH 7), then once the potential at which the faradaic current begins was identified, one of the two FTO (which acts as the generator) was held at such potential (up to 1.70 V vs RHE), while the other FTO electrode (acting as the collector) was set at -0.28 V vs RHE (the optimal oxygen reduction potential). Current at both working electrodes was recorded, subtracting at the generator the current at open circuit potential, i.e. before applying the oxygenic overpotential. The corrected current values were then integrated on origin allowing the calculation of the collection efficiency from the ratio between the areas calculated.

In order to measure O_2 evolved from the electrochemical water oxidation by IO-ITO|**QS** or **QS-TEG$_{lock}$** and **QS-TEG$_{unlock}$** the same method was employed, registering dark current in $NaHCO_3$ 0.1 M solution (pH 7) in the potential window used to screen photoanodes. IO-ITO|**QS** or **QS-TEG$_{lock}$** and **QS-TEG$_{unlock}$** photoanodes were held at applied bias where faradaic dark current start to be appreciable, thus acting as O_2 generators. In particular one potential was identified: the one at which falls the first dark oxygenic manifold (i.e. 1.40 V vs RHE for IO-ITO|**QS** and 1.45 V vs RHE for IO-ITO|**QS** and **QS-TEG$_{lock}$** and **QS-TEG$_{unlock}$**). While the FTO collector was held at -0.28 V vs RHE. Current at both the working electrodes was recorded, subtracting at the generator the current delivered at non faradaic potential (i.e. 1.00 V vs RHE). The corrected current values were then integrated on origin using the same procedure named before, allowing the calculation of the faradaic efficiency from the ratio between the areas calculated.

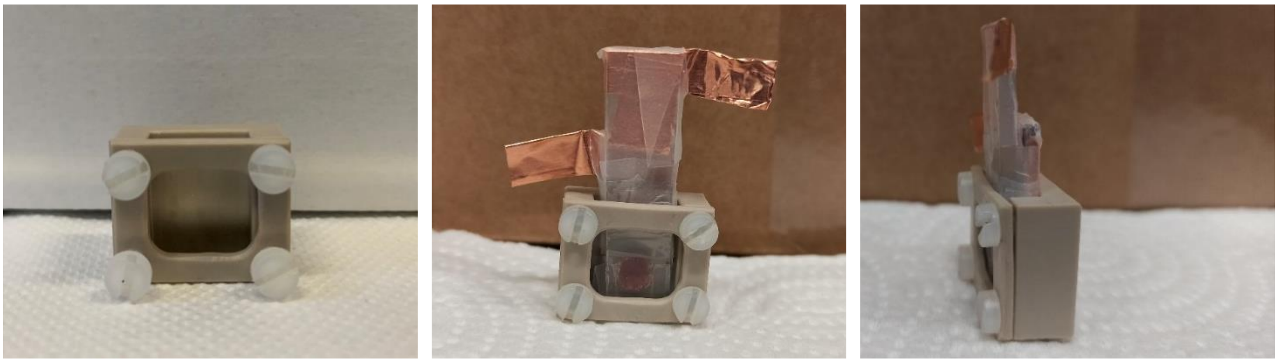

Picture of the cell made with polyether ether ketone and sealed with Teflon screws along with a Generator-Collector sandwich.

1.5 Preparation of IO-ITO

FTO cleaning was performed by sonicating the slides in Alconox® solution, in Milli-Q water and then in 2-propanol, every time for 10 minutes. After every step, FTO was rinsed with Milli-Q water. Subsequently, electrodes were sintered at 450 °C for 30 min. IO-ITO was prepared following a literature procedure.[11] A mixed dispersion of ITO nanoparticles (< 50 nm diameter) and polystyrene beads (750 nm diameter, 2.5% solids (w/v)) was prepared as follows: ITO nanoparticles (35 mg) were dispersed by sonication in 300 uL of a MeOH/water (6:1 v/v) mixture (300 μL taken from a mother solution of 10 mL) for 3 h. The dispersion of polystyrene beads (1 mL) was centrifuged for 1 hour at 4000 rpm, the supernatant removed, and the polystyrene pellet redispersed in MeOH (1 mL). The polystyrene dispersion was centrifuged again with same parameters. The supernatant was removed, and the dispersion of ITO nanoparticles added to the polystyrene pellet. This mixture was thoroughly vortexed and sonicated for 5 min in ice cold water (< 5 °C) to give the polystyrene–ITO dispersion. 30 minutes before deposition, electrodes were placed in a chamber with controlled atmosphere of the mother solution (MeOH/water, 6:1 v/v). On the electrodes, a magic tape mask to delimit the geometrical area of the deposition to 0.25 cm^2 was placed. An amount of 4.2 μL of the described polystyrene–ITO dispersion were deposited corresponding to a 10 ± 2 μm thick IO-ITO structure. The electrodes were then heated with 1°C min^{-1} to 500 °C and annealed at this temperature for 20 min. The IO-ITO electrodes were then cleaned by placing them in a mixture of 30% H_2O_2/ H_2O / 30% NH_4OH (1:5:1 v/v) at 70 °C for 15 min, rinsed with water, and heated for 1 h at 180 °C to give a contamination-free hydrophilic ITO surface.

1.6 Determination of IO-ITO ECSA (Electrochemical Surface Area) by Double-Layer Capacitance Measurements

Experiments were carried out using an Autolab PGSTAT302N potentiostat in a three-electrode setup using as counter electrode Au and as reference electrode Ag/AgCl (NaCl 3M). Potentials are then converted to RHE using the correlation $V(RHE) = V(Ag/AgCl) + 0{,}205 + 0{,}0592 \cdot pH$. CV scans for IO-ITO were recorded at scan rates in the range of 2 – 50 mV/s, spanning ca. ± 30 mV of the OCP, a range where no faradic processes occur.[12] The current values were divided by the geometric area of the electrodes. From the CV traces, the capacitive current was then calculated as $(J_a - J_c)/2$, where J_a and J_c are, respectively, the anodic and cathodic current densities at OCP. The resulting values (in A/cm^2) were plotted against the scan rate of the CV experiments (in V/s) and the data fitted with a linear equation. The slope of the linear regression gives the capacitance of the electrode (in F/cm^2). Assuming the FTO to be featureless (roughness factor, RF = 1 by definition), the RFs of IO-ITO electrodes can be calculated by dividing the corresponding capacitance values by the capacitance of the FTO foil used as the reference. For each anode, the OCP value was directly read on the potentiostat display after connecting all the three electrodes. The reading was stable.

1.7 Quantasome deposition on IO-ITO electrodes

IO-ITO|**QS** and **QS-TEG$_{lock}$** electrodes were prepared by co-deposition of the quantasome building blocks with 5:1 **PBI/Ru$_4$POM** stoichiometry yielding a nominal quantasome loading of 2.4, 4.8, 7.2, 9.6, 12 nmol cm^{-2} (Scheme S3).
The optimized deposition protocols is as follows: aliquots of a Ru$_4$POM solution in water are sequentially co-deposited with aliquots of the PBI-derivatives solution in water, as specified in the following Table, in order to reach the desired quantasome loading amount. 10:1 and 5:2 **PBI/Ru$_4$POM** stoichiometries were also co-deposited by varying the concentration of the mother solutions and the deposited aliquots.

Concentration and associated deposited volumes of the solution of **Ru$_4$POM** and **PBI^{2+}/PBI$_n$-TEG$_{lock}$.**

**Ru$_4$POM** (mM)[a]	Volume (μL)[b]	**PBI^{2+}** or **PBI$_n$-TEG$_{lock}$** (mM)[c]	Volume (μL)[d]	**QS** or **QS-TEG$_{lock}$** (nmol cm^{-2})[e]
0.12	2+2+1	0.6	2+2+1	2.4
0.24	2+2+1	1.2	2+2+1	4.8
0.36	2+2+1	1.8	2+2+1	7.2
0.12 + 0.36	(2+2+1) + (2+2+1)	0.6 + 1.8	(2+2+1) + (2+2+1)	9.6
0.24 + 0.36	(2+2+1) + (2+2+1)	1.2 + 1.8	(2+2+1) + (2+2+1)	12

[a]Concentration of deposited **Ru$_4$POM** catalyst solutions. [b]Volume of catalyst solution deposited. [c]Concentration of deposited **PBI^{2+}** or **PBI$_n$-TEG$_{lock}$** solutions. [d]Volume of perylene solution deposited. [e]Nominal loading on the electrode.

Scheme S3. Concentration and aliquots deposited of the mother solutions used in co-deposition.

1.8 Determination of surface-active sites of Ru$_4$POM

The active sites of **Ru$_4$POM** deposited on IO-ITO|**QS** or **QS-TEG$_{lock}$** and **QS-TEG$_{unlock}$** were calculated from the oxidation redox active peak at ca. 0.80 V vs RHE associated to a monoelectronic process as reported in Pourbaix diagram from literature.[13,14] Figure S30 shows an example of the calculation of the oxidation peak area of **Ru$_4$POM** extrapolated from cyclic voltammetry recorded at 10 mV s^{-1} in sodium hydrogen carbonate $NaHCO_3$ 0.1M (adjusted at pH 7).[15] Calculated area and charge are reported in Table S17. The number of electrons is extracted from charge. From the number of electrons calculated above, moles of **Ru$_4$POM** active sites can be obtained dividing for Avogadro constant.

2. Synthetic procedure

***2.1 Synthesis of PBI-YNYL*[16]**

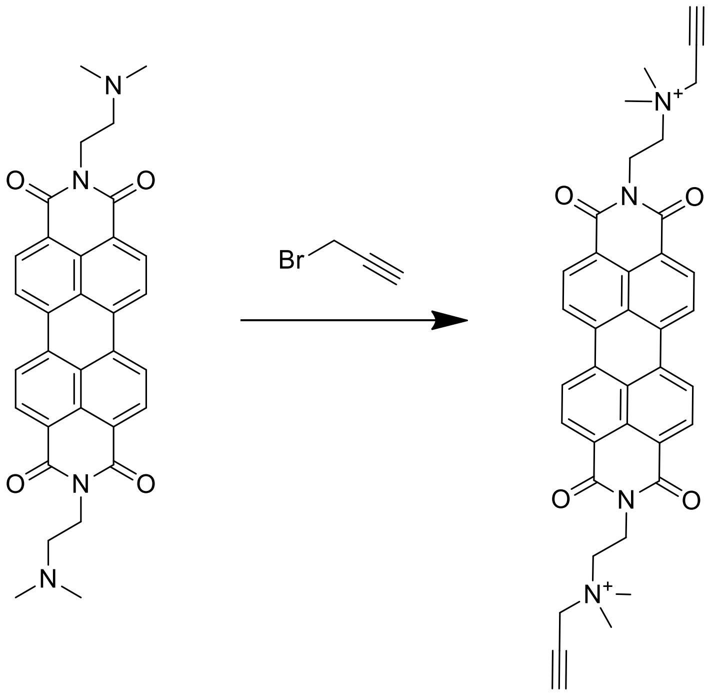

PBI-1 **PBI-YNYL**

PBI-YNYL: A suspension of 200 mg of **PBI-1** (0.37 mmol, MW = 532) in 1.6 ml of propargyl bromide 80% solution in toluene was stirred at 50°C overnight. The following day the product was precipitated adding 25 ml of THF to the mixture, which was consecutively filtered on a gooch washing abundantly with THF. The dry solid was then redissolved in milliQ water and filtered on a gooch to remove unreacted material. The filtrated was recovered and water was removed by rotavapor yielding 44 % of **PBI-YNYL.**

^{1}H NMR (200 MHz, d6-DMSO) δ = 8.54 (bs, 4H), 8.31 (bs, 4H), 4.63 – 4.52 (m, 8H), 4.14 (bs, 2H), 3.81-3.65 (m, 4H), 3.32 (s, 12H + H_2O). ^{13}C NMR (75 MHz, d6-DMSO) δ = 164.0, 135.1, 132.1, 129.7, 125.5, 123.5, 84.9, 73.6, 55.5, 51.6; MS (ESI+) calcd for $C_{38}H_{34}N_4O_4^{2+}$ $[M]^{2+}$, 305.1, found 305.1, FTIR (KBr): $\bar{\upsilon}$ = 3410, 2121, 1692, 1649, 1589, 1572 cm^{-1}

2.2 Synthesis of TEG-OTs

Adapted from previous reported procedure[16]

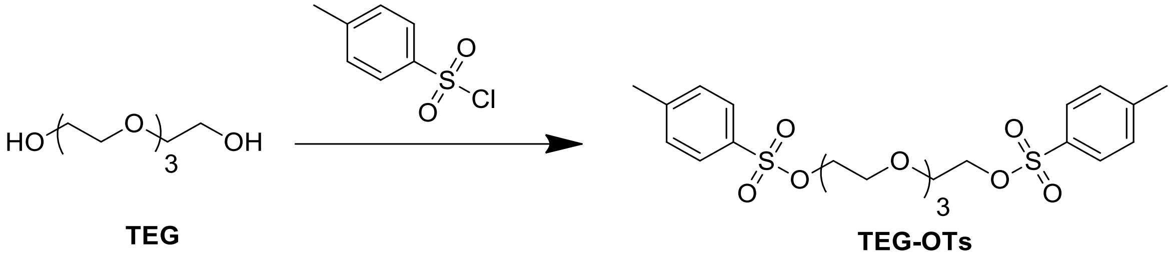

TEG-OTs: tetraethyleneglycol (0.35 g, 1.8 mmol) was added dropwise to a solution of p-toluenesulfonyl chloride (0.70 g, 3.7 mol) in anhydrous pyridine (5 mL) stirring in an ice bath at 0 °C, over 3 h. The resulting mixture was added to 10 mL ice water and extracted with CH_2Cl_2 (3×10 mL). The organic layer was then washed with 20 mL of HCl 6 M, followed by 20 mL of saturated NaCl solution and dried over anhydrous $MgSO_4$. The solvent was removed under reduced pressure affording a yellow oil in a 30% yield (0.27 g) without further purification.

^{1}H NMR (200 MHz, $CDCl_3$) δ = 7.77 (d, J = 8 Hz, 4H), 7.3 (d, J = 8 Hz, 4H), 4.10 (t, J = 4.8 Hz, 4H), 3.68 (t, J = 4.8 Hz, 4H), 3.68 – 3.55 (m, 28H), 2.41 (s, 6H); MS (ESI+) calcd for $C_{22}H_{32}O_9S_2^{2+}$ $[M+2H]^{2+}$ 252.1; found 252.0; FTIR (KBr): 2866, 1346, 1176, 1105 cm^{-1}.

2.3 Synthesis of TEG-N$_3$

Adapted from previous reported procedure[16]

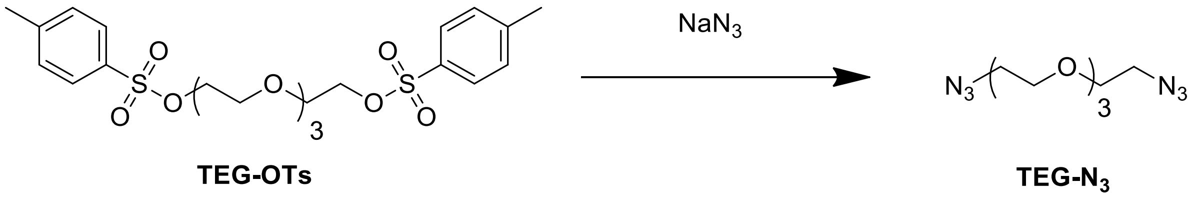

TEG-N$_3$: NaN_3 (130 mg, 2 mmol) and **TEG-OTs** (200 mg, 0.5 mmol, MW = 502) were dissolved in anhydrous DMF. The solution was degassed with N_2 and stirred at 50°C overnight. After cooling to room temperature, 100 ml of ice water were added to the reaction mixture and subsequently extracted with DCM. The organic fraction was washed with saturated NaCl and dried over magnesium sulphate to yield a 100 mg of a yellow solid in 80 % yield.

^{1}H NMR (200 MHz, $CDCl_3$): δ = 3.62, (m, 28 H), 3.35 (t, J=4Hz, 4H), 2.87 (t, J = 4 Hz, 4H); MS (ESI+) calcd for $C_8H_{17}N_6O_3$ $[M+H]^+$ = 245.1 found 245.0; FTIR (KBr): $\bar{\upsilon}$ = 2858, 2103, 1112 cm^{-1}.

2.4 Synthesis of PBI$_n$-TEG$_{lock}$

Adapted from previous reported procedure[16]

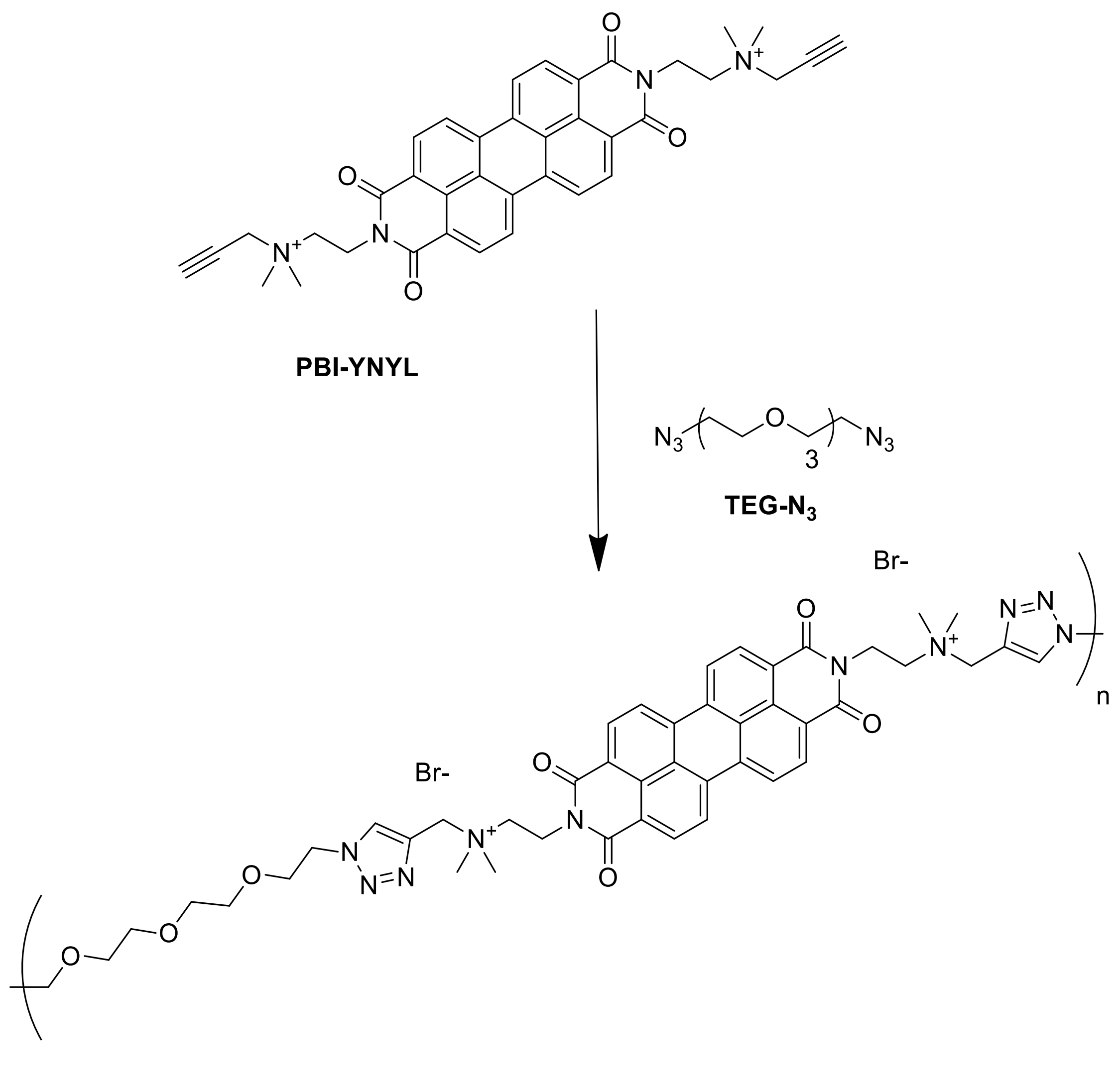

PBI$_n$-TEG$_{lock}$

PBI$_n$-TEG$_{lock}$: 20 mg **PBI-YNYL** (0.026 mmol, MW = 770) and 20 mg of **TEG-N$_3$** (0.08 mmol, MW = 244) were dissolved in 10 ml of MilliQ water and degassed with N_2 for 40 min. In a second flask 8 mg of $CuSO_4 \cdot 5H_2O$ and 4.5 mg of sodium ascorbate were dissolved in 5 ml degassed MilliQ water and transferred to the former flask. The solution was stirred at room temperature overnight under N_2. The following day the aqueous solution was extracted three time with DCM to remove the unreacted azidoglycole. The solution was concentrated under evaporation and passed through a Sephadex G50 column conditioned with water. The fractions were preliminary evaluated by UV-Vis in DMF to identify the successful locked aggregation system. Once the aqueous fraction was concentrated, acetonitrile was added and the solid was centrifugated three times

before yielding 18 mg of a dark red solid. The molecular weight of a **PBI$_n$-TEG$_{lock}$** monomer is estimated to be 1002 resulting in 70 % yield.

^{1}H NMR (400 MHz, D_2O) δ = 8.43, 7.04, 6.42, 3.92, 3.57, 3.24. FT-IR: $\bar{\upsilon}$ = 3369 (hydrated ammonium and PEG chains), 3425 (hydrated ammonium and PEG chains), 2915 (sp3 C-H, str.), 2879 (sp3 C-H str.), 1697 (imide C=O, str.), 1656 (imide C=O, str.), 1591 (C=C aromatic, str.), 1578 (C=C triazole, str.), 1472 (N=N, str.), 1439, 1403, 1386, 1364, 1341 (C-N, str.).

2.5 Synthesis of 2-(2-(2-methoxyethoxy)ethoxy)ethyl p-toluenesulfonate

Adapted from previous reported procedure[17]

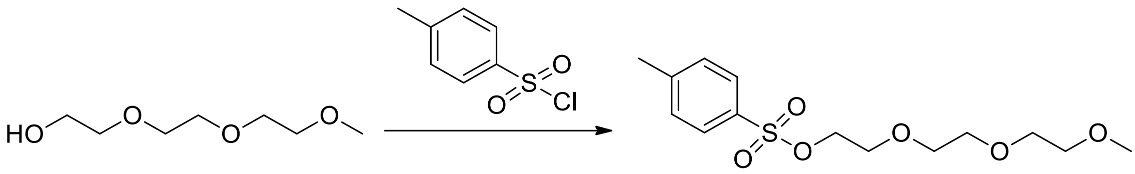

2-(2-(2-methoxyethoxy)ethoxy)ethyl p-toluenesulfonate*:* 2 mL of polyethylenglycol monomethylether (12.67 mmol) was added dropwise to a solution of 3.14 g of p-toluenesulfonyl chloride (16.5 mmol) and 3.6 mL of triethylamine (25 mmol) in 130 mL of MeCN at 0 °C. The mixture was stirred for 3 h at room temperature. The suspension was filtered and the solvent was removed under reduced pressure. The residue was dissolved in DCM and washed with 100 mL of 10% hydrochloric acid, followed by 100 mL of saturated NaCl solution and dried over anhydrous magnesium sulfate. The solvent was removed under reduced pressure affording a yellow oil. The product was separated by chromatographic column (n-hexane/ethyl acetate 3:1) to furnish a yellow oil in a 50% yield.

^{1}H NMR (300 MHz, $CDCl_3$) δ = 7.77 (d, J = 8 Hz, 2H), 7.31 (d, J = 8 Hz, 2H), 4.13 (t, J = 4.8 Hz, 2H), 3.66 (t, J = 4.8 Hz, 2H), 3.62 – 3.46 (m, 8H), 3.34 (s, 3H), 2.42 (s, 3H). FTIR (KBr): 2854, 1345, 1112, 1096 cm^{-1}.

2.6 Synthesis of TEG-OMe

Adapted from previous reported procedure[17]

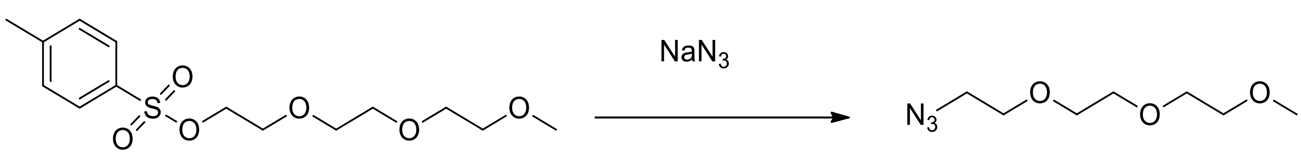

TEG-OMe: 0.44 g of NaN_3 (6.7 mmol) and 0.83 g of 2-(2-(2-methoxyethoxy)ethoxy)ethyl p-toluenesulfonate (2.6 mmol) were dissolved in 15 mL of anhydrous DMF. The solution was degassed with N_2 and stirred at 50 °C overnight. After cooling to room temperature, 100 ml of ice water were added to the reaction mixture and subsequently extracted with ethyl acetate (2 x 50 mL). The organic fraction was washed with saturated NaCl

and dried over magnesium sulfate. The solvent was removed under reduced pressure affording a yellow oil without further purification in 64 % yield.

^{1}H NMR (300 MHz, $CDCl_3$) δ = 3.67, (m, 8 H), 3.56 (m, 2H), 3.38 (m, 5H). FTIR (KBr): ῡ = 2832, 2120, 1098 cm^{-1}.

2.7 Synthesis of PBI-TEG$_{unlock}$

Adapted from previous reported procedure[16,17]

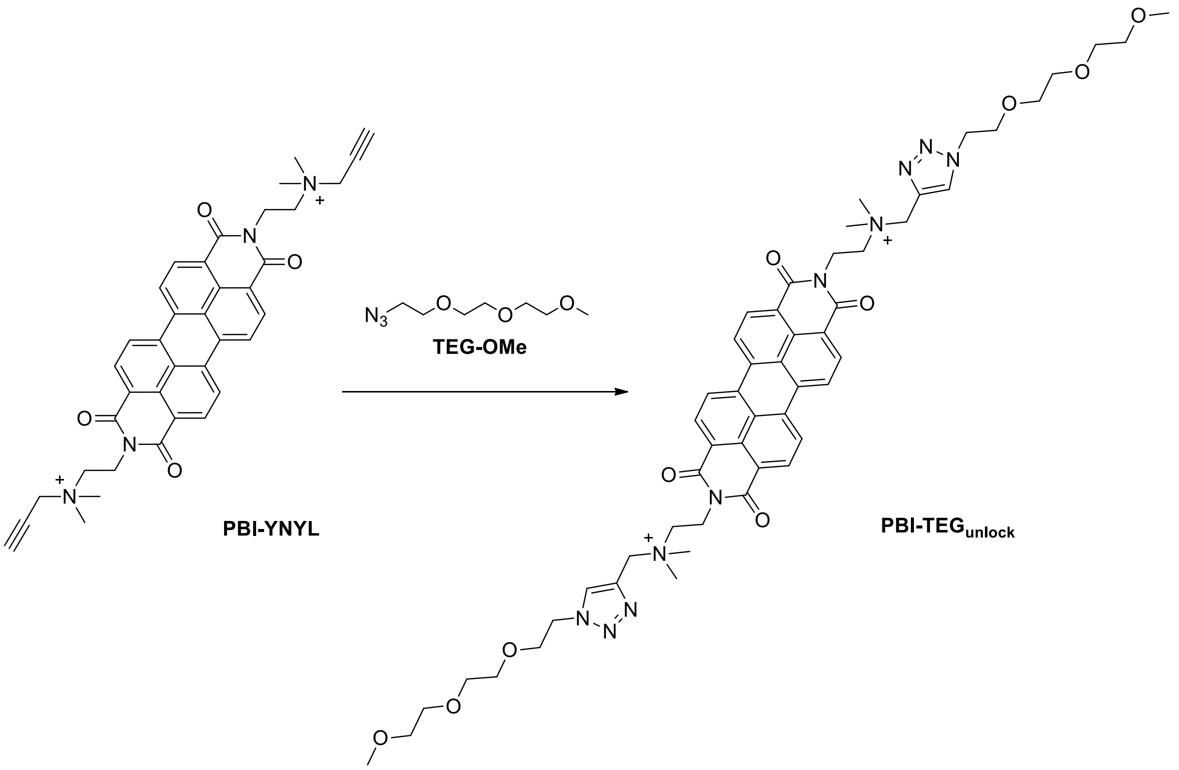

***PBI-TEG$_{unlock}$*:** 25 mg **PBI-YNYL** (0.032 mmol, MW = 770) and 155 mg of **TEG-OMe** (0.08 mmol, MW = 189) were dissolved in 20 ml of Milli-Q water and degassed with N_2 for 30 min. In a second flask 10 mg of $CuSO_4 \cdot 5H_2O$ and 8 mg of sodium ascorbate were dissolved in 10 ml degassed MilliQ water and transferred to the former flask. The solution was stirred at room temperature overnight under N_2. The following day the aqueous solution was extracted three times with diethyl ether to remove the unreacted azidoglycole. The solution was concentrated under evaporation and passed through a Sephadex G50 column conditioned with water. Once the aqueous fractions were concentrated, acetone was added and the solid was centrifugated three times before yielding 22.8 mg (62%) of a dark red solid.

^{1}H NMR (300 MHz, D_2O) δ = 8.63, 8.13, 5.02, 4.29, 4.06-3.33, 2.27. (Attribution in Figure S4) FT-IR: ῡ = 3430 (hydrated ammonium and PEG chains), 2878 (sp3 C-H, str.), 1698 (imide C=O, str.), 1648 (imide C=O, str.), 1596 (C=C aromatic, str.), 1579 (C=C triazole, str.), 1461 (N=N, str.), 1404, 1368, 1337 (C-N, str.), 1109 (C-O-O, str. PEG chains). MS (ESI+) calcd for $C_{52}H_{64}N_{10}O_{10}^{2+}$ $[M]^{2+}$ 494.2 found 494.2.

3. Supporting Figures

3.1 Benchmarking of state-of-the-art photoanodes for water oxidation

Table S1. Literature performances of state-of-the-art photoanodes in comparison with present work.

Photoanode[a]	Light Intensity (mW/cm^2)[b]	Experimental Conditions[c]	E (V) vs RHE[d]	t_{max} (min)[e]	I(t=0) – I (t_{max})[f]	F.E. (O_2) (%)[g]	Loss %[h]	Ref.
IO-ITO|PSII	10	I	0.88	60 min (1 h)	500 – 10 μA/cm^2 (MET via DCBQ)	75 ± 4	98	11
IO-ITO|P_{Os}-PSII		L			550 – 60 μA/cm^2 (MET via DCBQ)	//	89	18
TiO_2|QAP-C16| Zr^{4+}|RuOEC	100	M	0.3	360 min (6 h)	75-2 μA/cm^2	27%	97	19
TiO_2|RuDye-IrO_2	7.77	N	0.55	240 min (4 h)	(30 -10) – (2-1) μA/cm^2	20	93	20
TiO_2|RuDye-Ru_4POM	33	O<br>P	0.55<br>0.63	300 min (5 h)	33 – 2.5 μA/cm^2	> 80	85	21
nanoITO|MV^{2+} -Ru(II)Dye-Fe(II) Donor-Ru(II)Cat.	100	Q	0.77	60 min (1 h)	250-50 μA/cm^2	67%	80	22
TiO_2|PMI| 30ALD|IrIrCat	200	R	0.65	10 min	25 – 9 μA/cm^2	22	64	23
TiO_2|-RuDye-RuCat	100	S	0.54	120 min (2 h)	1.7-0.8 mA/cm^2	90%	53	24
SnO_2|PMPDI|CoO_x	100	T	0.82	5 min	30 – 14 μA/cm^2	31 ± 7	53	10
ITO|PMPDI|CoO_x			1.52		150 – 100 μA/cm^2	80 ± 15	33	25
IO-ITO|**QS-TEG$_{lock}$**	850	U	1.12	60 min (1 h)	30 – 13 μA/cm^2	95 ± 9	56	**This work**
IO-ITO|**QS-TEG$_{unlock}$**	850	U	1.12	60 min (1 h)	30 – 8 μA/cm^2	100 ± 10	73	**This work**
SnO_2/TiO_2|RuDye |NiO|-RuCat *	100	V	0.87	120 min (2 h)	[NiO 20 cycles] 1.15 – 0.98 mA/cm^2	84 - 86	15	26

[a] Photoanode components. [b] Incident light intensity. [c] Experimental conditions (light source, pH and buffer): I, red light, 679 nm,10 mW/cm^2, pH = 6.5, $CaCl_2$ (20 mM), MES (40 mM) and 5% glycerol; L, red light, 679 nm,10 mW/cm^2, pH = 6.5, $CaCl_2$ (20 mM), $MgCl_2$ (15 mM), KCl (50 mM) and MES (40 mM); M, White light, 400 nm filter, 100 mW/cm^2, pH = 7, 0.1 M phosphate buffer, 0.5 M KNO_3; N, 450 nm, 7.77 mW/cm^2, pH = 5.75, Na_2SiF_6 (30 mM) – $NaHCO_3$, Na_2SO_4 500 mM; O, 450 nm, 33 mW/cm^2, pH = 5.8, Na_2SiF_6/$NaHCO_3$ (200 mM in Na); P, 450 nm, 33 mW/cm^2, pH = 7.2, 50 mM lutidine 200 mM $NaClO_4$; Q, White light, 400 nm filter, 100 mW/cm^2, pH = 4.65, 0.1 M acetate buffer, 0.5 M $NaClO_4$; R, White light, 400 nm filter, 200 mW/cm^2, pH = 2.5, 1.0 M Na_2SO_4; S, White light, 400 nm filter, 100 mW/cm^2, pH = 5.8, 0.1M acetate buffer 0.5M $NaClO_4$; T, V = 0.82, 400 – 710 nm, 100 mW/cm^2, V = 1.52, 315 – 710 nm, 100 mW/cm^2, pH = 7, 0.1 M KPi buffer; U, White light, 450 nm filter, 850 mW/cm^2, pH = 7, $NaHCO_3$ 0.1 M adjusted using HCl; V, White light, 100 mW/cm^2, pH = 4.65, 0.1 M acetic acid/acetate buffer, 0.4 M $NaClO_4$. [d] Voltage applied. [e] Time of continuous illumination. [f] Initial photocurrent and final photocurrent registered. As initial value, the photocurrent after 1 minute is considered; values are obtained either from the text or derived from experimental figures. [g] Oxygenic faradaic efficiency of the photoanodes as obtained in PEC three electrodes set-up. [h] Percentage loss of photocurrent calculated as Loss % = [I(t=0) – I (t_{max})]/I(t=0). * Metal oxide electron transfer relay.

3.2 Fourier Transform Infrared Spectroscopy

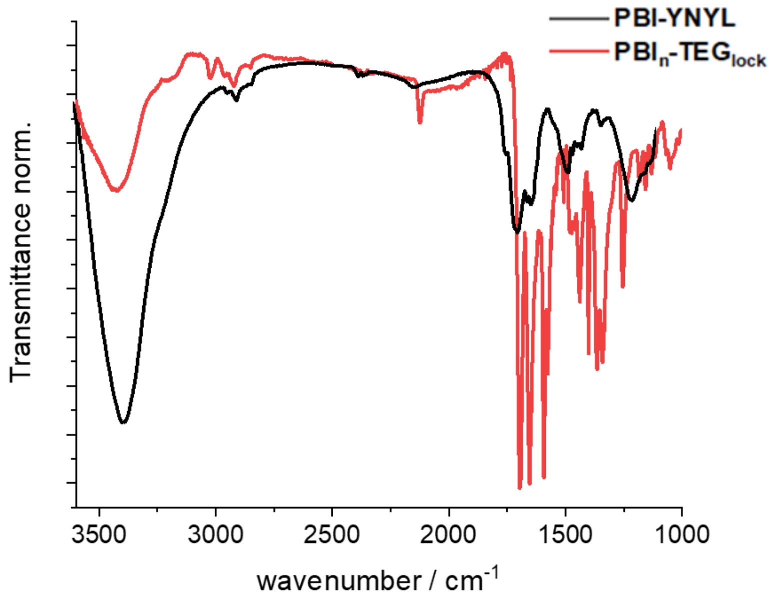

Figure S1. FTIR of the precursor **PBI-YNYL** and of the locked **PBI$_n$-TEG$_{lock}$** in the region 3600 cm^{-1} – 1000 cm^{-1}.

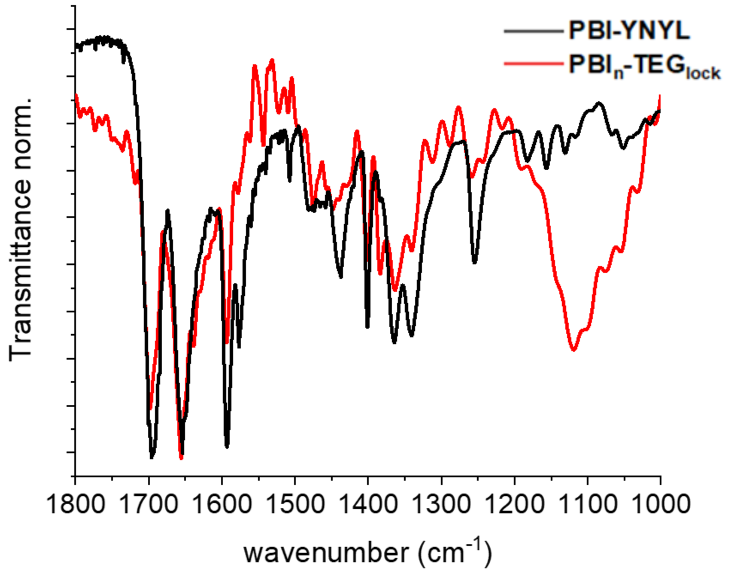

Figure S2. Zoom in the 1800 to 1000 cm^{-1} region of normalized FTIR of the precursor **PBI-YNYL** and the locked **PBI$_n$-TEG$_{lock}$**.

3.3 NMR characterization

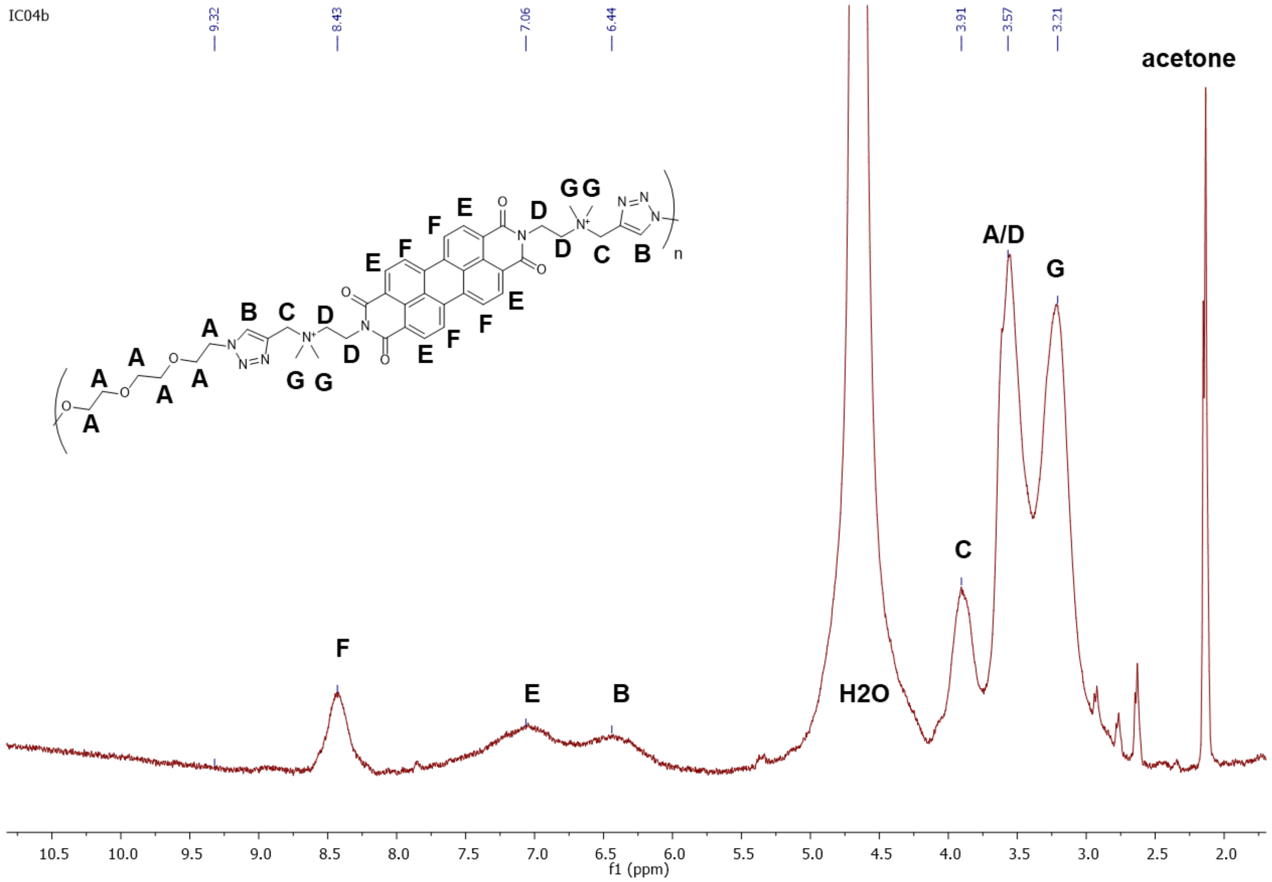

Figure S3. ^{1}H NMR (400 MHz, D_2O) of 2 mM **PBI$_n$-TEG$_{lock}$.** δ = 8.43, 7.04, 6.42, 3.92, 3.57, 3.24.

3.4 Absorption Spectroscopy

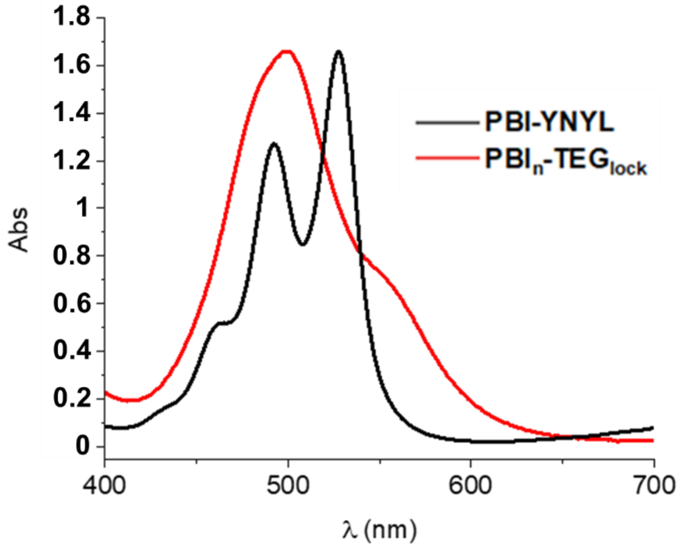

Figure S4. Superimposed absorption spectra of **PBI-YNYL** and **PBI$_n$-TEG$_{lock}$** in DMF showing the multi-PBI aggregation of the locked superstructure.

3.5 DOSY NMR studies

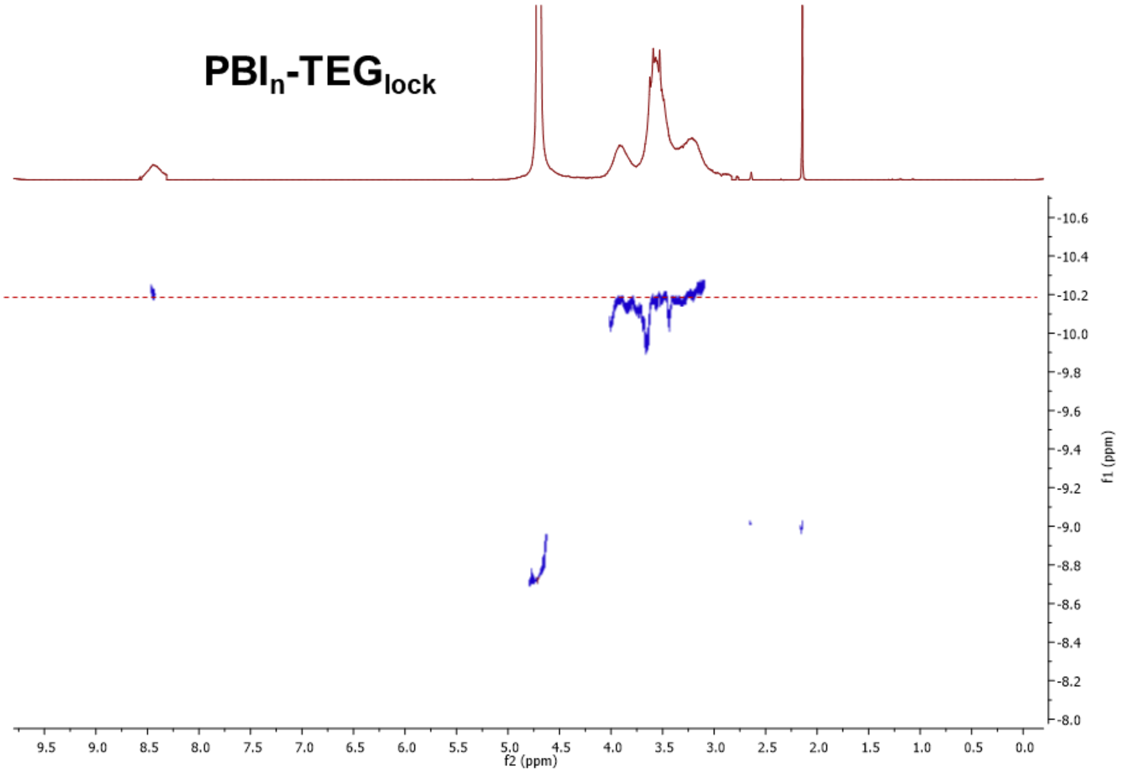

Figure S5. ^{1}H-Diffusion Ordered Spectroscopy (DOSY) NMR experiments registered for **PBI_n-TEG_{lock}** 2 mM in D_2O, 400 MHz NMR, at 20 °C.

3.6 Translational diffusion coefficient of PBIs

Table S2. Diffusion coefficient values, determined by DOSY-NMR, of reported PBI aggregates associated to the size (in terms of N_{PBI}) of the aggregates.

N_{PBI}[a]	D(m^2s^{-1})[b]	Solvent	Ref.
Monomer	3.40×10^{-10}	MCH	[27]
Monomer	$9.0x10^{-10}$	$CDCl_3$	[28]
Dimer	$2.11x10^{-10}$	$CDCl_3$/MCH	[29]
10	1.57×10^{-10}	MCH	[27]
20	7.36×10^{-11}	MCH	[27]
20	1.26×10^{-10}	$CDCl_3$/MCH	[30]
20*	$6.3 x 10^{-11}$	H_20	**This work**

a) Average number of aggregated molecules N of PBI obtained from DOSY estimated as $N_{DOSY} \approx (D_{ref}/D)^3$. b) Translational diffusion coefficient.
***N_{PBI}** is an approximation obtained from dividing the diameter obtained from Stoke-Einstein equation $D = k_BT/(6\pi\eta R)$, where diameter is 2R = d, by the reported columnar PBI-PBI distance.[31] This is a rough estimate, since it is not possible to have a monomeric reference PBI analogous in water in order to apply the formula $N_{DOSY} \approx (D_{ref}/D)^3$.

3.7 Fluorescence Quantum yield and decay dynamics

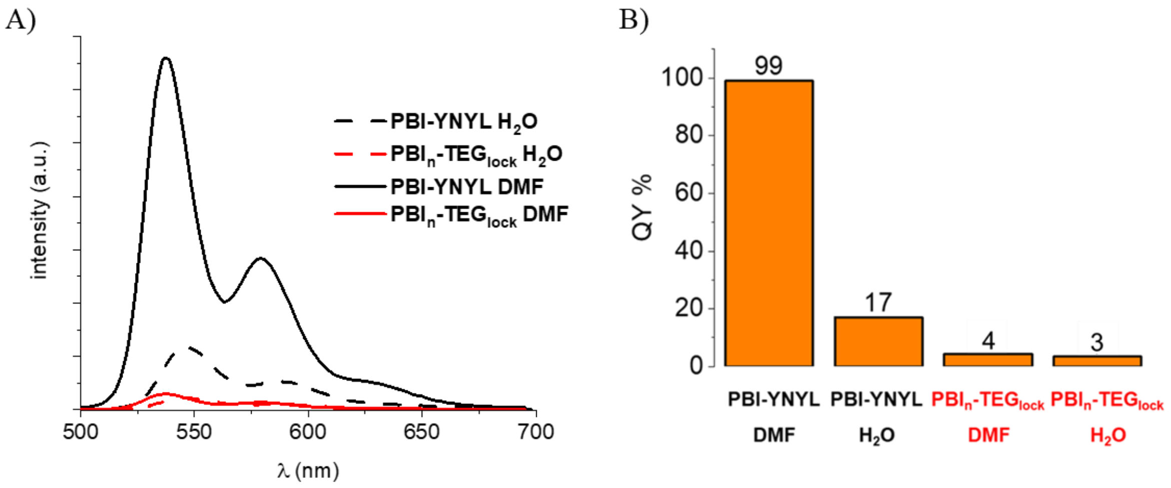

Figure S6. A) Fluorescence emission spectra of **PBI-YNYL** and **PBI$_n$-TEG$_{lock}$**. Experiments performed in H_2O and DMF at 298 K with 490 nm excitation wavelength. Intensity was normalized on the fluorescence quantum yield of the sample, calculated as reported in Methods section. B) Fluorescence Quantum Yield of **PBI-YNYL** and **PBI$_n$-TEG$_{lock}$**. Experiments performed in H_2O and DMF at 298 K. Fluorescensce quantum yields were calculated as reported in Equipment and Methods section 1.2.

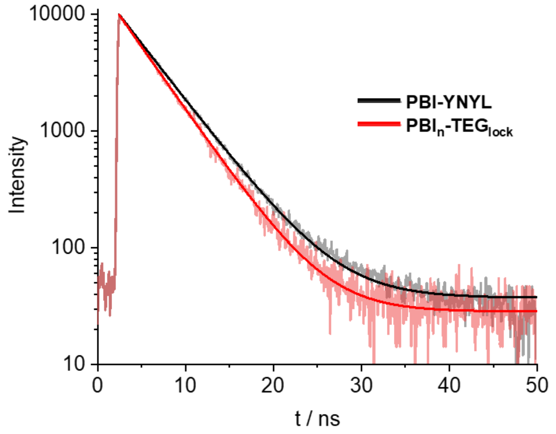

	PBI-YNYL	PBI$_n$-TEG$_{lock}$
$\tau_{Fl,1}$ / ns (amplitude)	4.3 (100%)	1.3 (1.5%)
$\tau_{Fl,2}$ / ns (amplitude)		4.0 (98.5%)

Figure S7. Fluorescence decay profiles of **PBI-YNYL** and **PBI$_n$-TEG$_{lock}$** in DMF under photoexcitation at 402 nm and probe wavelength at 545 nm.

3.8 PBI$_n$-TEG$_{lock}$ absorption and emission spectra and redox properties

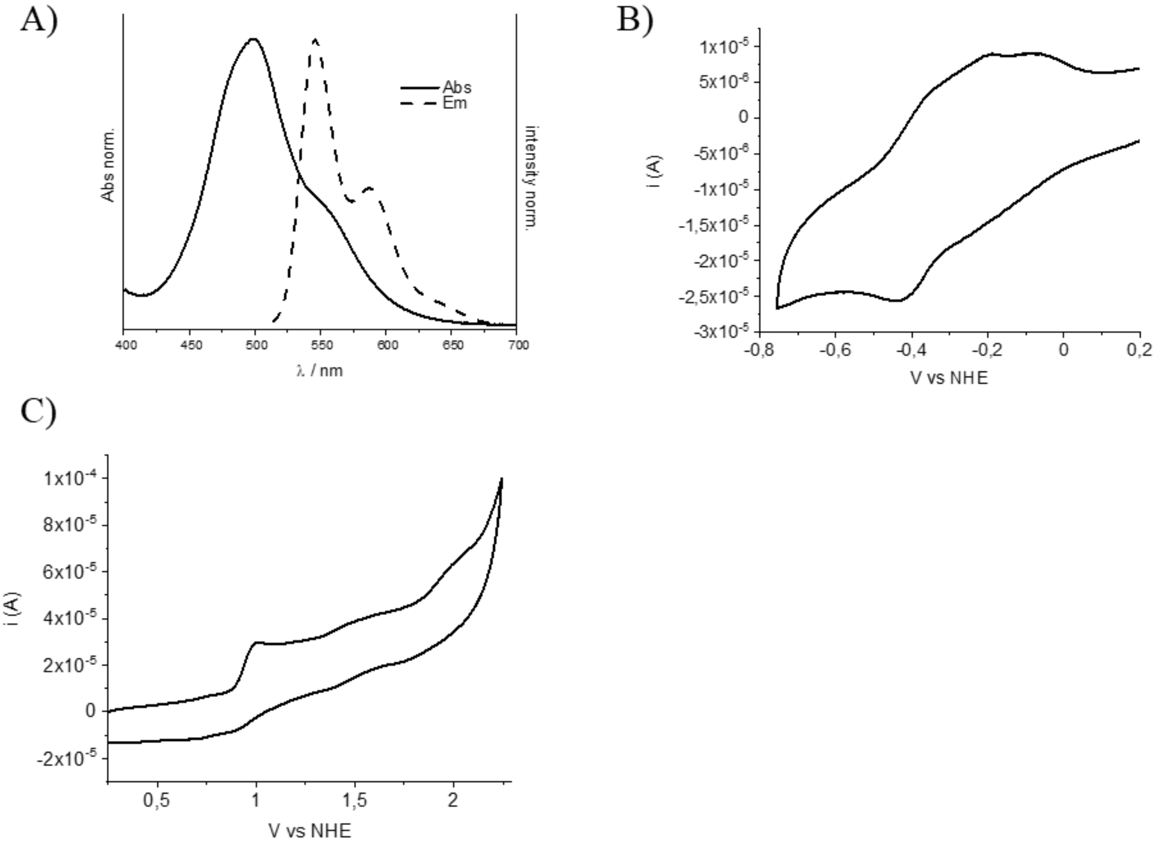

Figura S8. A) Normalized absorption and emission spectra of **PBI$_n$-TEG$_{lock}$** in water (λ_{ex} = 500 nm). The intersection of the curves at 534 nm was employed to estimate E_{00} equal to 2.32 eV. B) Representative cyclic voltammetry of **PBI$_n$-TEG$_{lock}$** recorded in MeCN showing the cathodic reduction processes. C) Representative cyclic voltammetry of **PBI$_n$-TEG$_{lock}$** recorded in MeCN showing the anodic oxidation processes. All measurements have been calibrated to SCE standard electrode using Fc/Fc^+ as an internal potentiometric standard Fc/Fc^+ = 0.45 V vs SCE in MeCN and then converted to NHE ($V(NHE) = V(SCE) + 0{,}241$). Experimental conditions: 0.1 M $TBAPF_6$, scan rate = 100 mV s^{-1}, glassy carbon working electrode, Ag/AgCl reference electrode, Pt counter electrode.

Subsequently, the excited state redox potential of the (**PBI$_n$-TEG$_{lock}$**$^{*/\bullet-}$) couple was calculated according to equation.[32]

E(**PBI$_n$-TEG$_{lock}$**$^{*/\bullet-}$) = $E^{1/2}$ (**PBI$_n$-TEG$_{lock}$**$^{/\bullet-}$) + E_{00}

Thus:

E(**PBI$_n$-TEG$_{lock}$**$^{*/\bullet-}$) = (-0.06 + 2.32)V = 2.26 V vs NHE

3.9 Spectrophotometric and ζ -potential Titrations of the PBI$_n$-TEG$_{lock}$ / Ru$_4$POM assembly

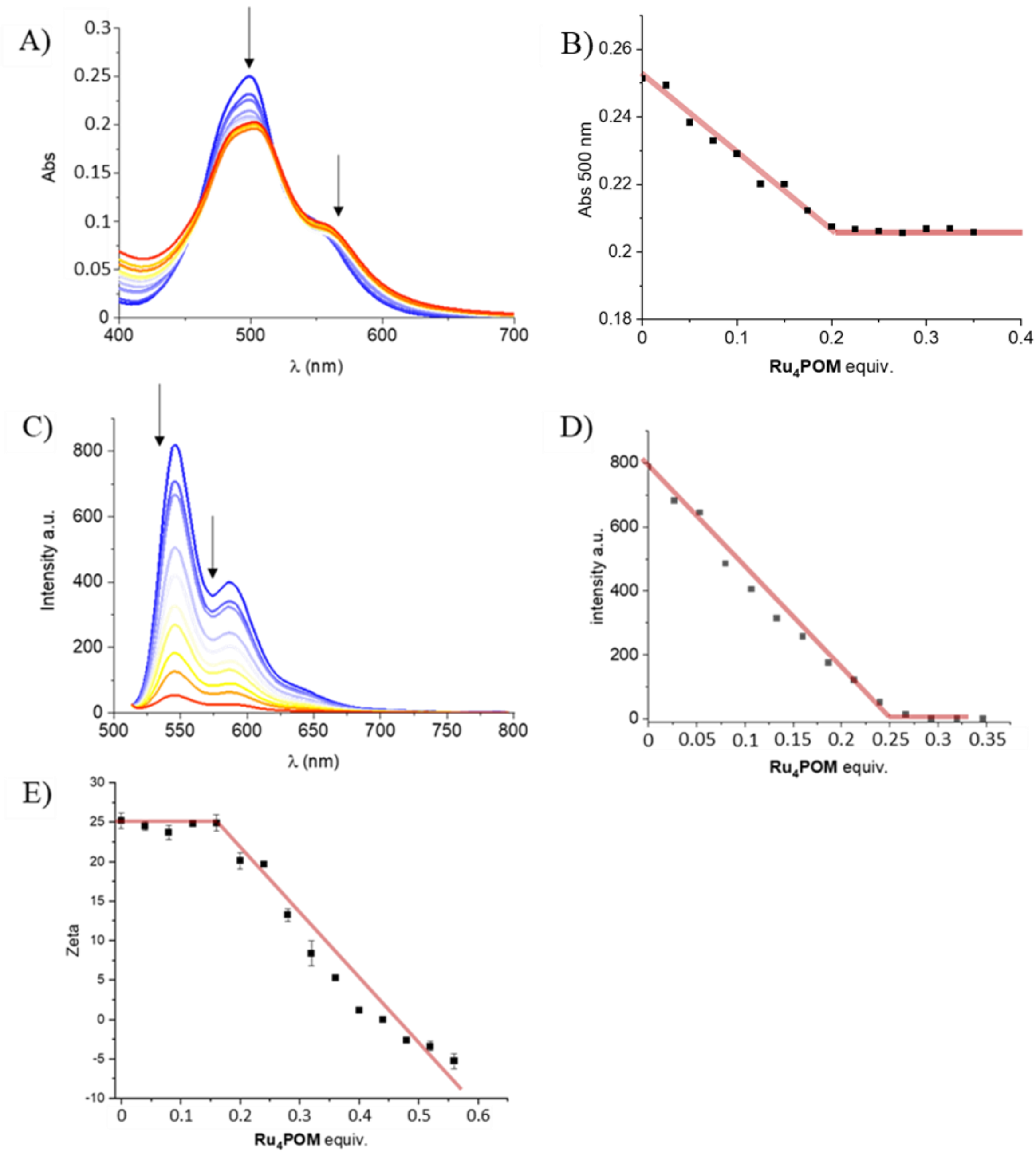

Figure S9. A) UV-Vis spectrophotometric titration of **PBI$_n$-TEG$_{lock}$** (12.5 µM, blue trace) in milliQ water with **Ru$_4$POM** (up to 2.5 µM, 0.2 eq., red trace). B) plot of the absorbance at 500 nm versus the equivalents of **Ru$_4$POM**. C) Emission spectrophotometric titration of **PBI$_n$-TEG$_{lock}$** (12.5 µM, blue trace) in milliQ water with **Ru$_4$POM** (up to 3.12 µM, 0.25 eq., red trace). Excitation wavelength 500 nm. D) plot of the emission intensity at 550 nm versus the equivalents of **Ru$_4$POM.** E) ζ-Potential titration of **PBI$_n$-TEG$_{lock}$** (12.5 µM) upon addition of **Ru$_4$POM** (up to 15 µM, 0.6 eq.) in milliQ water.

3.10 Stability of QS and QS-TEG$_{lock}$ colloids

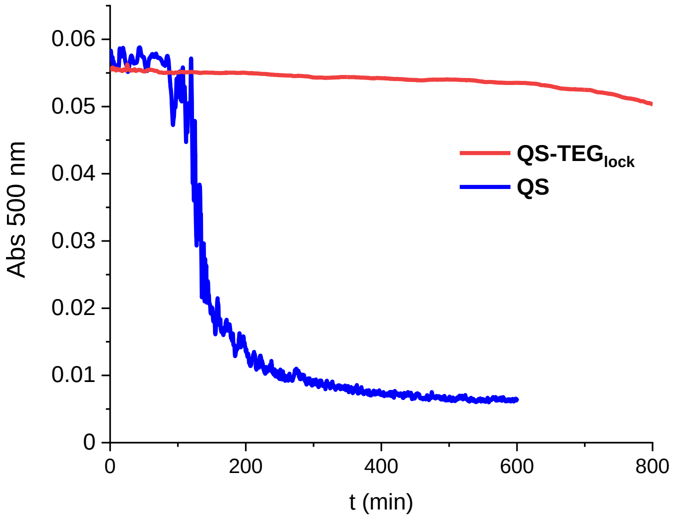

Figure S10. Stability of **QS** and **QS-TEG$_{lock}$** colloids against over-aggregation in water monitored by recording the solution absorption at λ = 500 nm as a function of time (min).

3.11 Scanning Electron Microscopy imaging of the IO-ITO electrodes

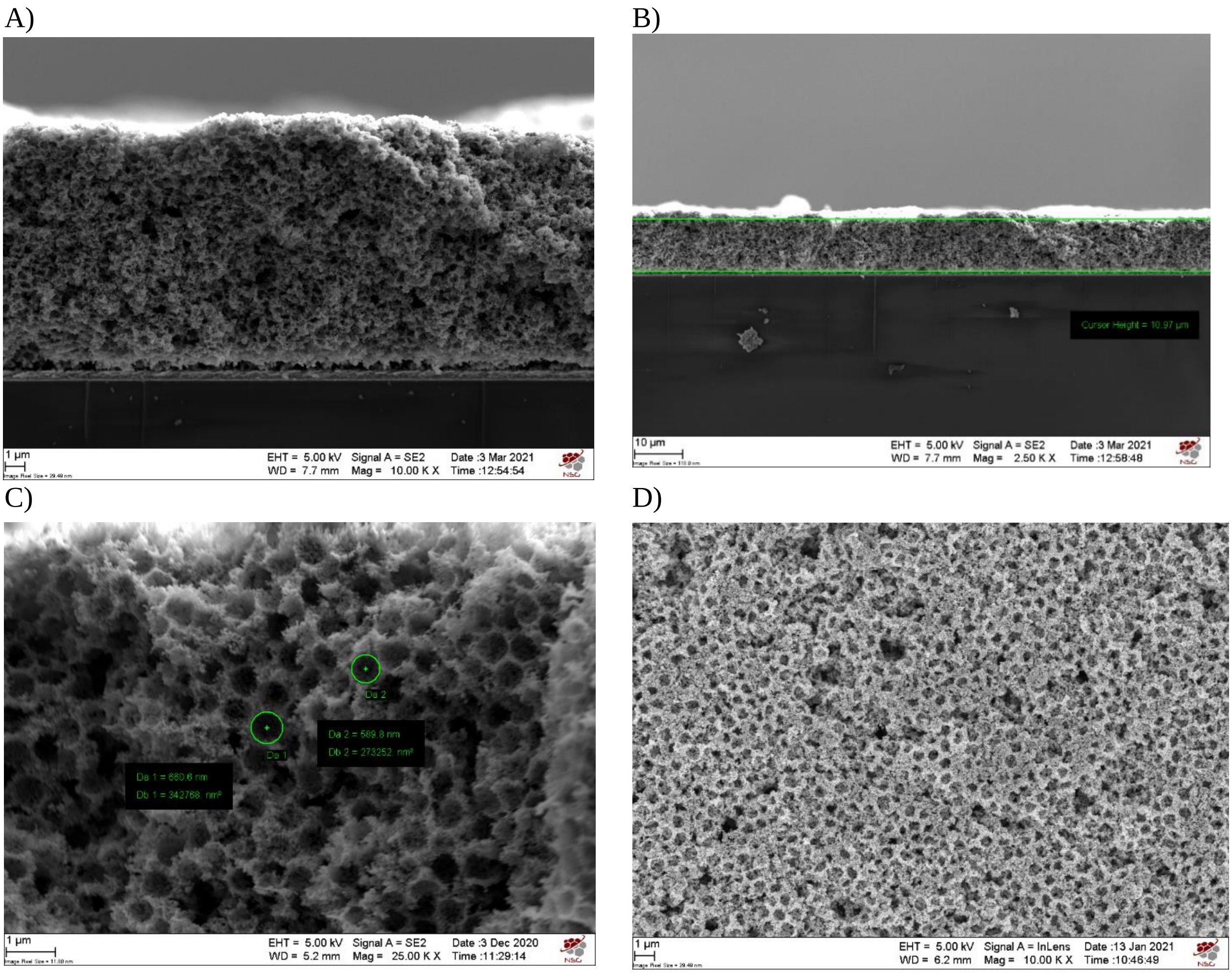

E) F)

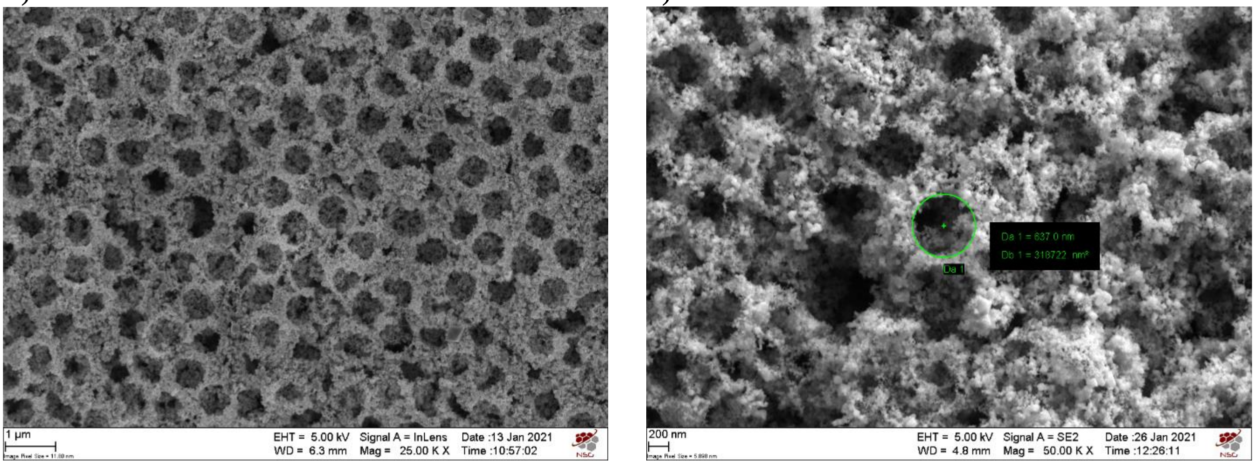

Figure S11. SEM images at different magnification factors of IO-ITO samples prepared. A)-C) cross-section images. D)-F) top view images. Measured thickness is 10 ± 2 µm. Pores dimension is 600 ± 100 nm.

3.12 Determination of electrochemical surface area

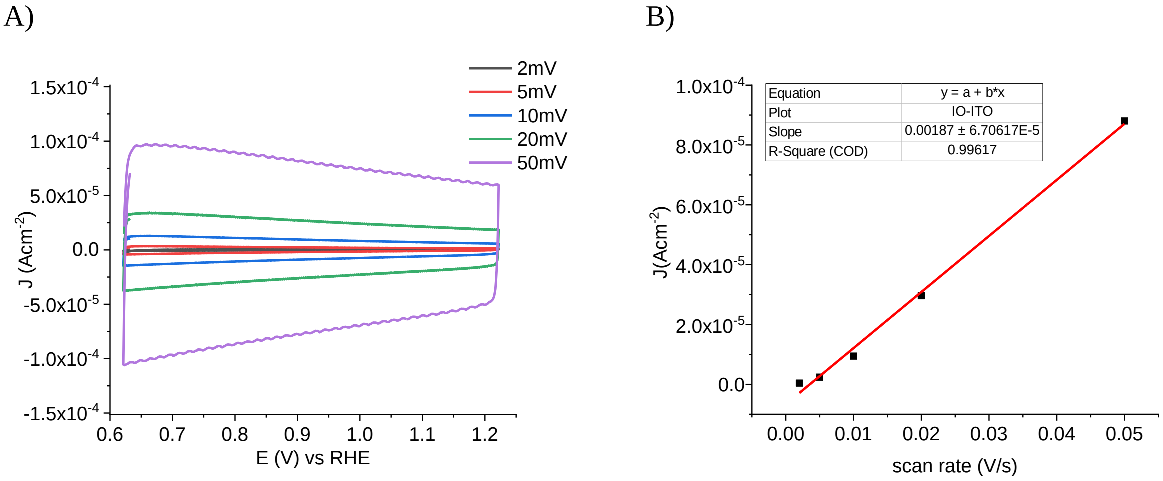

Figure S12. Double Layer Capacitance (DLC) measurements. A) Cyclic Voltammetry (CV) scans around the open circuit potential (OCP) for IO-ITO registered in 0.1 M $NaHCO_3$ (pH 7). The corresponding linear fit is reported in (B). The calculated slope corresponds to the electrode capacitance, from which the RF can be calculated as reported in the experimental section. Figures are related to one representative electrode. Average values (up to 3 samples) are reported in Table S3.

Table S3. Capacitance values and surface roughness factors calculated from the double layer capacitance experiments reported in Figure S12[33]. The surface roughness factor for the flat FTO electrode used as the reference is defined to be 1. Average values for 3 IO-ITO and FTO electrodes are reported.

	**FTO**	**IO-ITO**
Electrode Capacitance (F/cm^2)[a]	$(2 \pm 0.2) * 10^{-6}$	$(1.9 \pm 0.4) * 10^{-3}$
Roughness Factor[b]	1	950 ± 200

[a]Slope of the linear fit. [b]Electrode capacitance divided by FTO capacitance.

3.13 Chopped light chronoamperometry (CLCA) at different PBI^{2+}/Ru_4POM and PBI_n-TEG_{lock}/Ru_4POM Ratio

A) B)

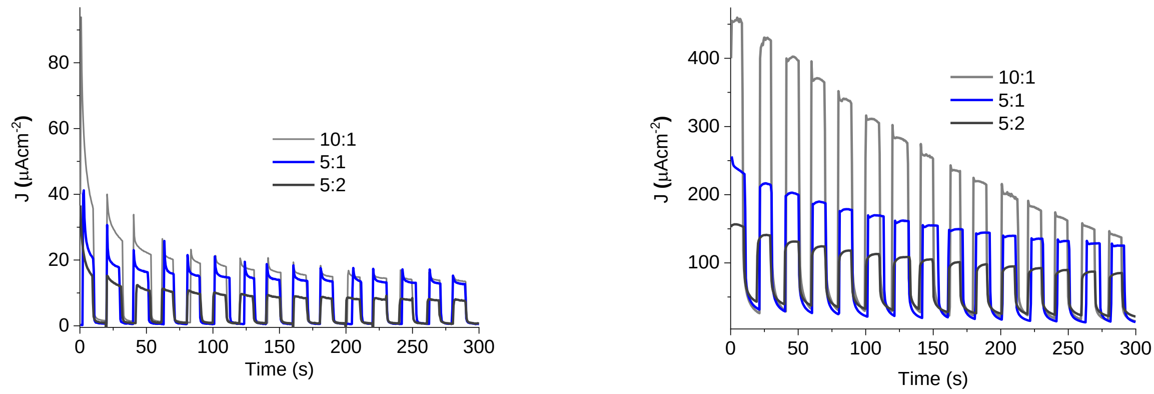

Figure S13. Representative chopped light chronoamperometries of IO-ITO|**QS** as a function of **PBI^{2+}/Ru_4POM** ratio registered at A) 1.12 V vs RHE and B) 1.52 V vs RHE in 0.1 M $NaHCO_3$, pH 7, with solar simulator equipped with AM 1.5 G filter, 850 mW cm^{-2}, λ > 450 nm. Samples prepared via co-deposition of 12 nmol cm^{-2} of **QS** on IO-ITO electrodes. Ratio **PBI^{2+}/Ru_4POM** 10:1 (light grey), 5:1 (blue), 5:2 (dark grey).

A) B)

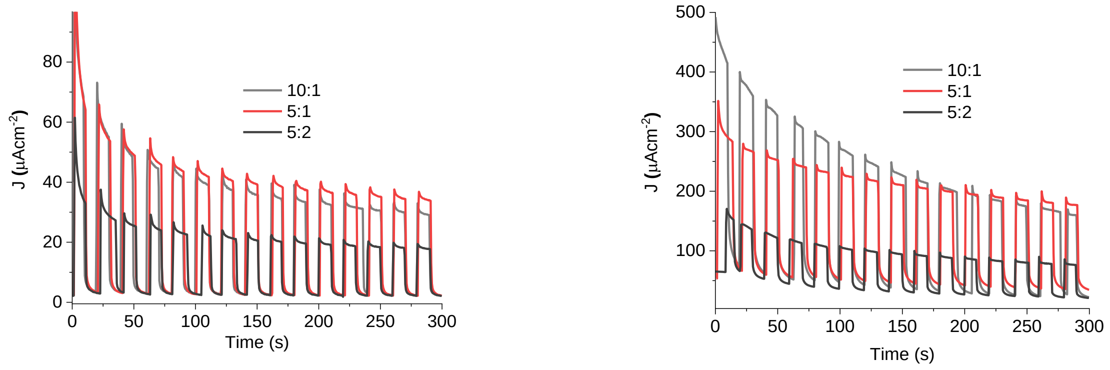

Figure S14. Representative chopped light chronoamperometries of IO-ITO|**QS-TEG_{lock}** as a function of **PBI_n-TEG_{lock}/Ru_4POM** ratio registered at A) 1.12 V vs RHE and B) 1.52 V vs RHE in 0.1 M $NaHCO_3$, pH 7, with solar simulator equipped with AM 1.5 G filter, 850 mW cm^{-2}, λ > 450 nm. Samples prepared via co-deposition of 12 nmol cm^{-2} of **QS-TEG_{lock}** on IO-ITO electrodes. Ratio **PBI_n-TEG_{lock}/Ru_4POM** 10:1 (light grey), 5:1 (red), 5:2 (dark grey).

3.14 Faradaic efficiency of Oxygen production (FE_{O2}) by IO-ITO|QS and IO-ITO|QS-TEG$_{lock}$ determined with generator collector method

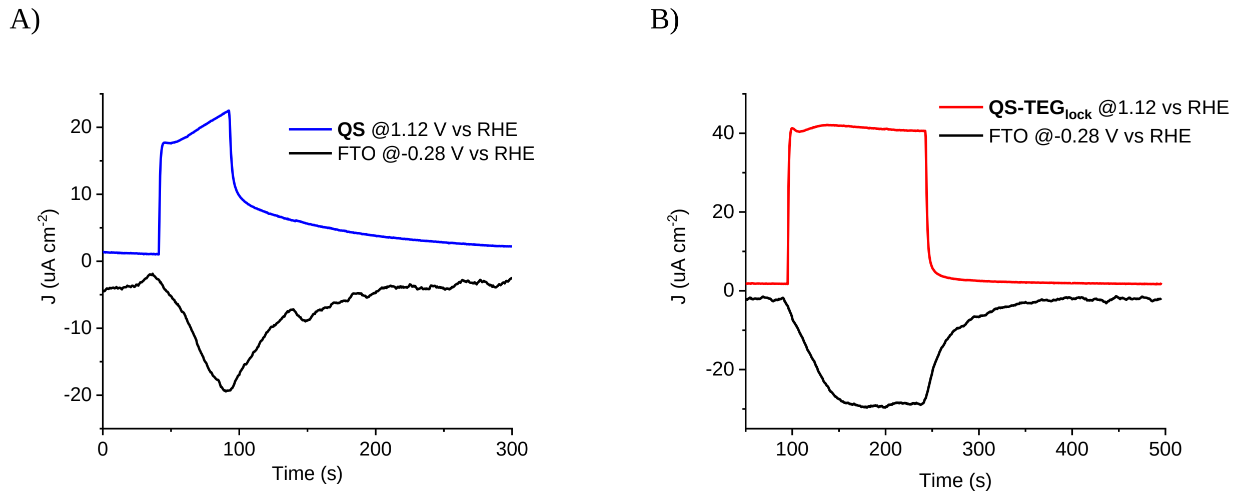

Figure S15. Photocurrent traces of A) IO-ITO|**QS** (blue trace) and B) IO-ITO|**QS-TEG$_{lock}$** (red trace) (12 nmol cm^{-2} loading) registered at 1.12 V vs RHE in 0.1 M $NaHCO_3$, pH 7, with solar simulator equipped with AM 1.5 G filter, 850 mW cm^{-2}, λ > 450 nm and corresponding O_2 reduction trace registered at the FTO collector under -0.28 V vs RHE applied bias (black trace).

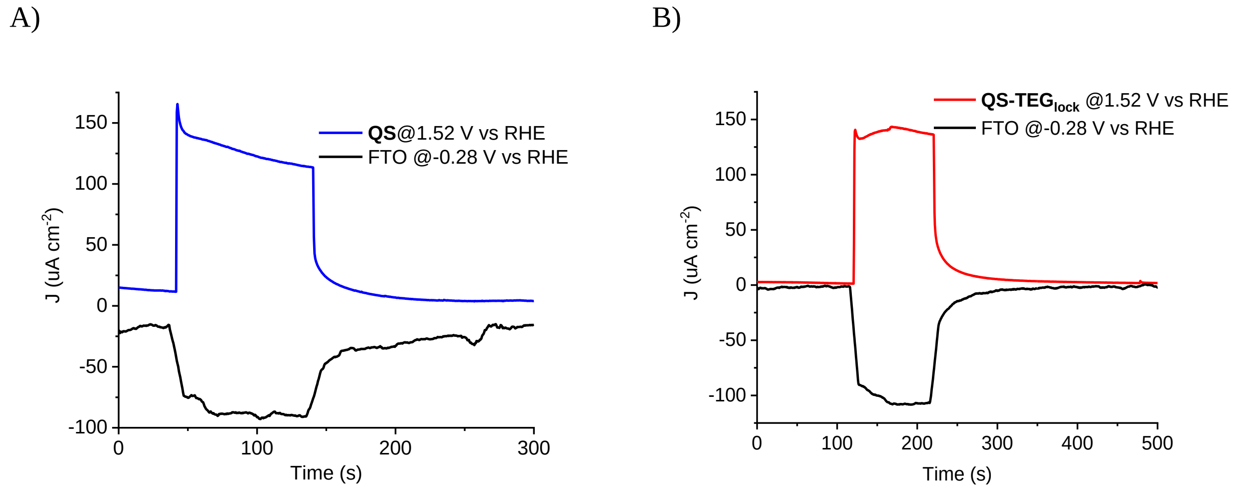

Figure S16. Photocurrent traces of a) IO-ITO|**QS** (blue trace) and b) IO-ITO|**QS-TEG$_{lock}$** (red trace) (12 nmol cm^{-2} loading) registered at 1.52 V vs RHE in 0.1 M $NaHCO_3$, pH 7, with solar simulator equipped with AM 1.5 G filter, 850 mW cm^{-2}, λ > 450 nm and corresponding O_2 reduction trace registered at the FTO collector under -0.28 V vs RHE applied bias (black trace).

Table S4. FE_{O2} , determined with generator – collector method,[1,8–10] of IO-ITO|**QS** and IO-ITO|**QS-TEG$_{lock}$** prepared via co-deposition of **QS** (12 nmol cm^{-2}), **QS-TEG$_{lock}$** (12 nmol cm^{-2}) on IO-ITO electrodes.

Photoanode	E (V) vs RHE[a]	FE_{O2} (%)[b]	E (V) vs RHE[c]	FE_{O2} (%)[d]
IO-ITO|**QS**	1.12	96 ± 6	1.52	100 ± 10
IO-ITO|**QS-TEG$_{lock}$**	1.12	95 ± 9	1.52	100 ± 10

[a,c] Voltage applied at generator electrode reported vs RHE. [b,d] Faradaic efficiency measured with a collection efficiency of 78%. Experiments are registered in 0.1 M $NaHCO_3$, pH 7 with solar simulator equipped with AM 1.5 G filter, 850 mW cm^{-2}, λ > 450 nm. Every experiment is repeated at least 3 times and the value here reported is the mean value.

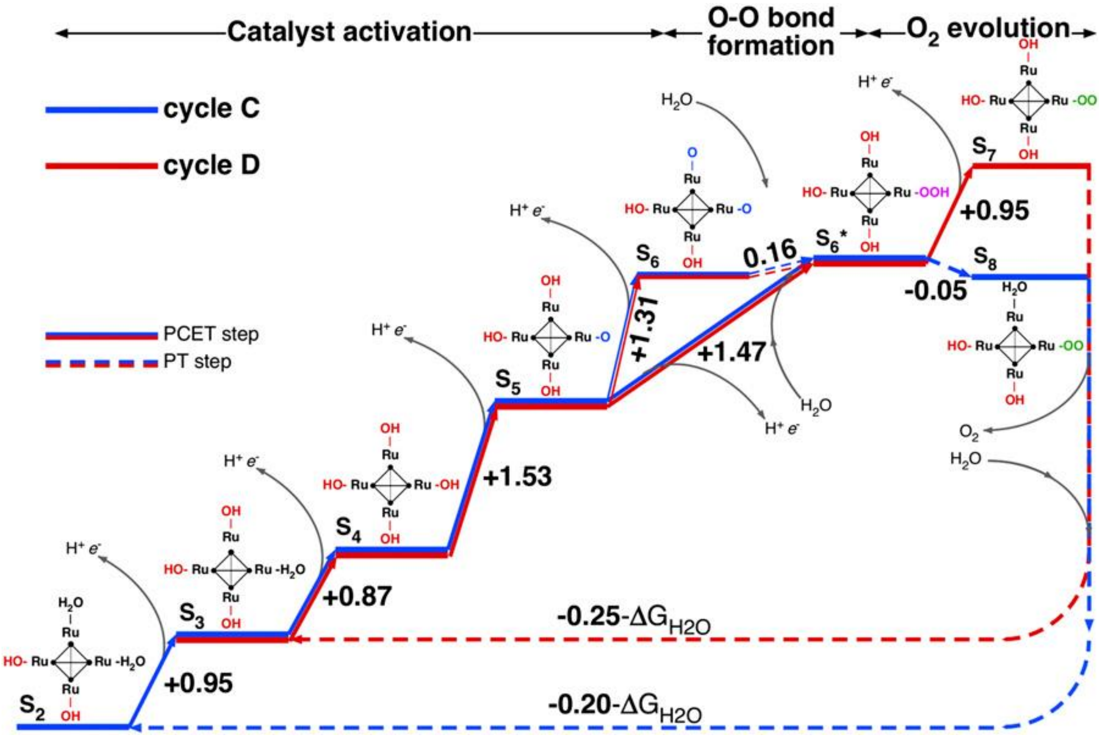

Scheme S1. Proposed mechanistic manifolds for Ru_4POM WOC, reprinted with permission from reference 8 in the manuscript (PNAS, 2013, 110 (13), 4917–4922).

3.15 PEIS analysis for IO-ITO|QS and IO-ITO|QS-TEG$_{lock}$

A) B)

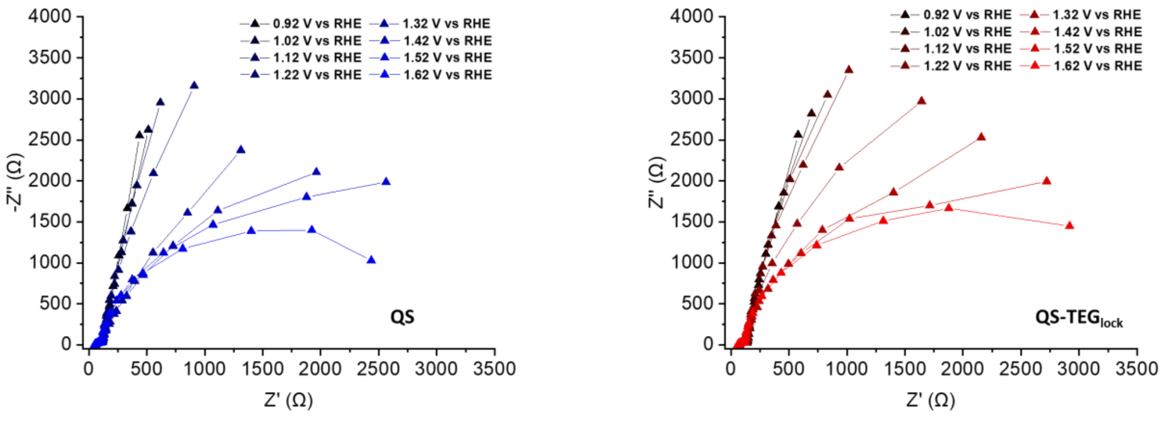

Figure S17. PEIS Nyquist plots at different applied potentials of A) IO-ITO|**QS** and B) IO-ITO|**QS-TEG$_{lock}$** registered in 0.1 M $NaHCO_3$, pH 7, with solar simulator equipped with AM 1.5 G filter, 850 mW cm^{-2}, $\lambda > 450$ nm. Samples prepared via co-deposition of 12 nmol cm^{-2} of **QS** and **QS-TEG$_{lock}$** on IO-ITO electrodes.

A) B)

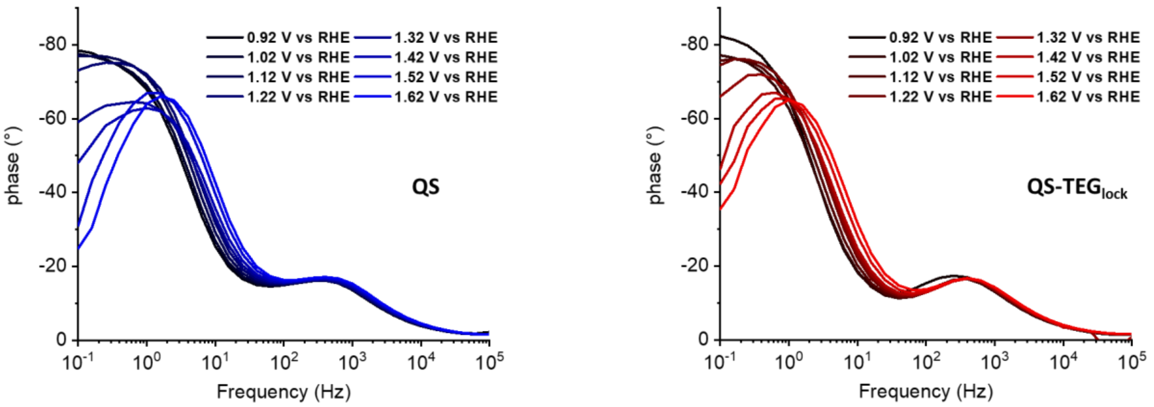

Figure S18. Bode phase plots at different applied potentials of A) IO-ITO|**QS** and B) IO-ITO|**QS-TEG$_{lock}$** registered in 0.1 M $NaHCO_3$, pH 7, with solar simulator equipped with AM 1.5 G filter, 850 mW cm^{-2}, $\lambda > 450$ nm. Samples prepared via co-deposition of 12 nmol cm^{-2} of **QS** and **QS-TEG$_{lock}$** on IO-ITO electrodes.

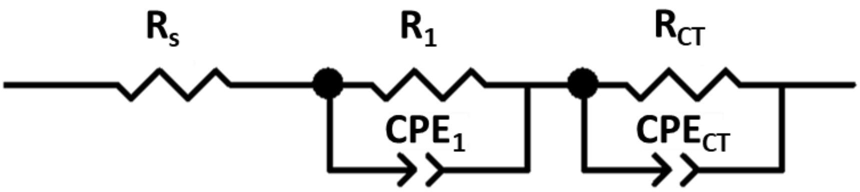

Figure S19. Equivalent electric circuit (EC) used for interpretation of the PEIS data. **R_s**: series resistance associated to cell resistance, **R_1**: resistance independent of the applied potential, associated to the FTO|IO-ITO interface, **R_{CT}**: potential dependent resistance associated to interfacial charge transfer. **CPE_1** and **CPE_{CT}** are related capacitances.

Table S5. R_{CT} and CPE_{CT} values obtained from fitting PEIS data of IO-ITO|**QS** and IO-ITO|**QS-TEG$_{lock}$** with Z View. Samples are prepared via co-deposition of **QS** (12 nmol cm^{-2}), **QS-TEG$_{lock}$** (12 nmol cm^{-2}) on IO-ITO electrodes.

	IO-ITO|**QS**		IO-ITO|**QS-TEG$_{lock}$**	
**E (V) vs RHE[a]**	**R_{CT} (kΩ cm^{-2})[b]**	**CPE_{CT} (mF cm^{-2})[c]**	**R_{CT} (kΩ cm^{-2})[b]**	**CPE_{CT} (mF cm^{-2})[c]**
0.9	40 ± 10	2.8 ± 0.6	15 ± 1	2.70 ± 0.01
1.0	19 ± 3	2.5 ± 0.6	9.5 ± 0.7	2.630 ± 0.002
1.1	11.0 ± 0.7	2.0 ± 0.5	6.8 ± 0.3	2.242 ± 0.001
1.2	6.4 ± 0.3	1.7 ± 0.4	5.6 ± 0.1	1.930 ± 0.002
1.3	2.70 ± 0.07	2.2 ± 0.6	2.8 ± 0.5	1.802 ± 0.003
1.4	1.62 ± 0.04	1.6 ± 0.4	1.38 ± 0.02	1.572 ± 0.001
1.5	1.15 ± 0.01	1.0 ± 0.2	1.13 ± 0.01	1.277 ± 0.003
1.6	0.881 ± 0.006	0.8 ± 0.2	0.98 ± 0.01	1.073 ± 0.002

[a]Voltage applied at working electrode reported vs RHE. [b] Interfacial charge-transfer resistance. [c]Capacitance associated to interfacial charge-transfer resistance. Experiments are registered in 0.1 M $NaHCO_3$, pH 7 with solar simulator equipped with AM 1.5 G filter, 850 mW cm^{-2}, $\lambda > 450$ nm. Every measure is repeated at least 3 times and the value here reported is the mean value.

3.16 IPCE and APCE of IO-ITO|QS and IO-ITO|QS-TEG$_{lock}$

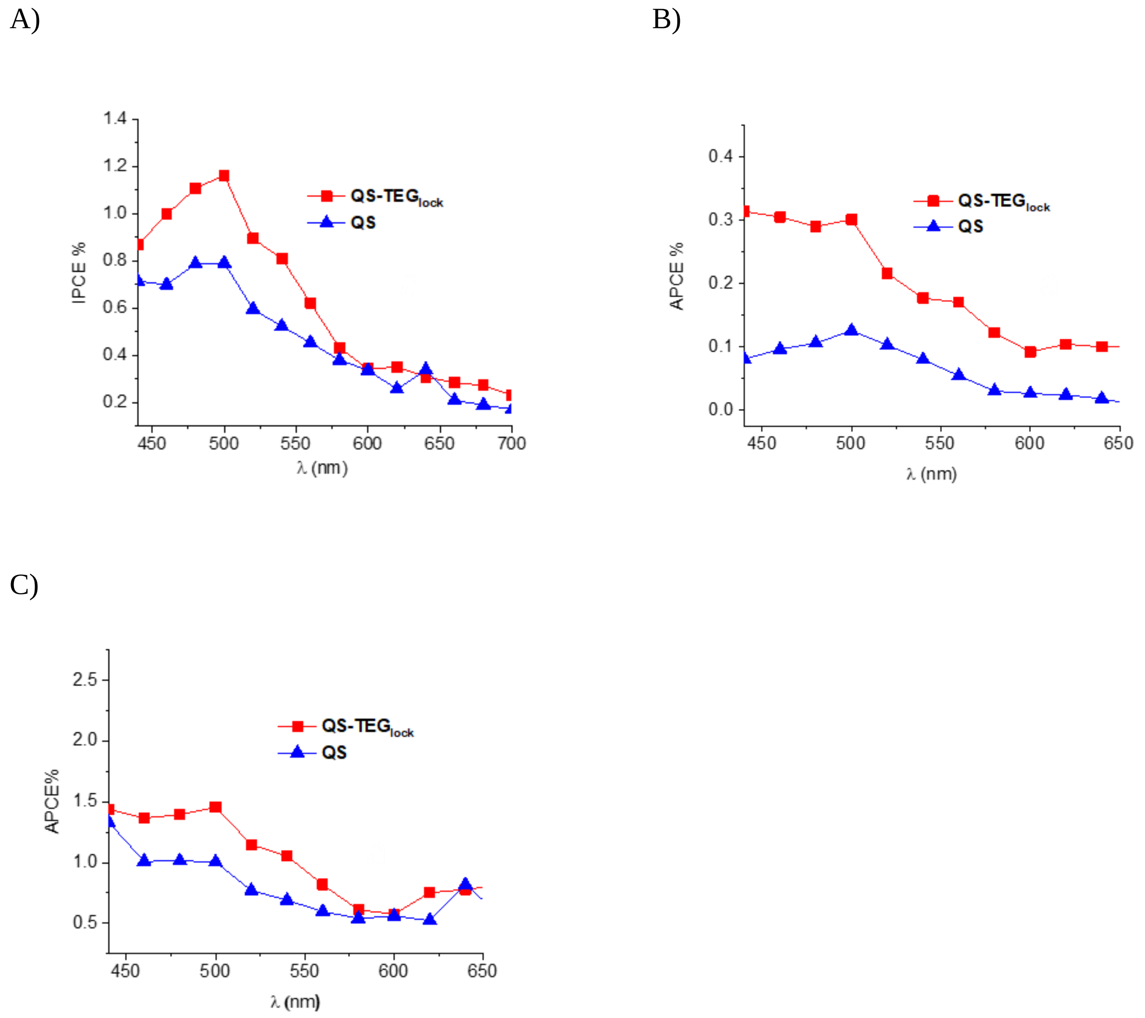

Figure S20. A) Incident photon-to-current efficiency (IPCE) at 1.52 V vs RHE and absorbed photon-to-current efficiency (APCE) at B) 1.12 V vs RHE and at C) 1.52 V vs RHE of IO-ITO|**QS** (blue trace) and IO-ITO|**QS-TEG$_{lock}$** (red trace) (12 nmol cm^{-2} loading) in 0.1 M $NaHCO_3$, pH 7, as a function of the irradiation wavelength.

Table S6. IPCE% and APCE% of IO-ITO|**QS** and IO-ITO|**QS-TEG$_{lock}$** prepared via co-deposition of **QS** (12 nmol cm^{-2}), **QS-TEG$_{lock}$** (12 nmol cm^{-2}) on IO-ITO electrodes.

**Photoanode**	**IPCE %[a] (at 1.12 V vs RHE)**	**APCE %[b] (at 1.12 V vs RHE)**	**IPCE %[c] (at 1.52 V vs RHE)**	**APCE %[d] (at 1.52 V vs RHE)**
IO-ITO|**QS**	0.1 ± 0.01	0.13 ± 0.01	0.8 ± 0.03	1 ± 0.03
IO-ITO|**QS-TEG$_{lock}$**	0.25 ± 0.015	0.3 ± 0.015	1.2 ± 0.05	1.5 ± 0.05

[a] Incident photon to current conversion and [b]absorbed photon to current conversion efficiency = $IPCE(\lambda)/(1 - 10^{-A(\lambda)})$ reported at λ = 500 nm. Mean values of 3 experiments.

3.17 Quantasome Loading

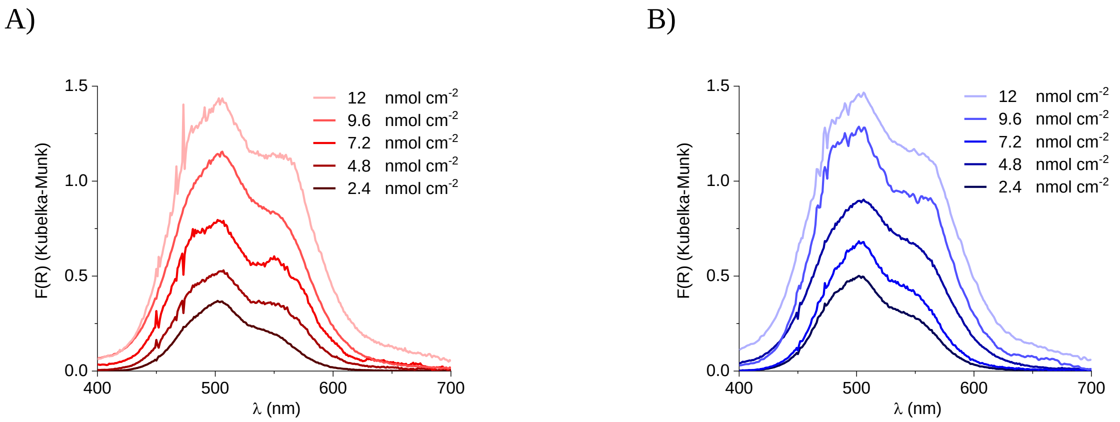

Figure S21. Diffuse reflectance spectra converted in Kubelka Munk units of fresh co-deposited A) ITO|**QS-TEG$_{lock}$** (red traces) and B) IO-ITO|**QS** (blue traces) as a function of loading from 2.4 to 12 nmol cm^{-2}. Reflectance spectra are registered on 3 samples per each loading.

Table S7. Photocurrent density at several time delays and normalized J/Jo (scaled with respect to the minimum value, Jo) response of IO-ITO|**QS-TEG$_{lock}$** as a function of quantasome loading registered by chronoamperometries at 1.12 V vs RHE and corresponding diffuse reflectance intensity converted in KM units F(R) values at 500 nm.

**QS-TEG$_{lock}$ Loading (nmol cm^{-2})[a]**	**J @ 1.12 V vs RHE [t=10s] (μA cm^{-2})[b]**	**J @ 1.12 V vs RHE [t=110s] (μA cm^{-2})[c]**	**J @ 1.12 V vs RHE [t=300s] (μA cm^{-2})[d]**	**J/J_0 [t=10s] @ 1.12 V vs RHE[e]**	**$F(R)_0$ (KM units)[f]**
2.4	19 ± 2	11 ± 1	8.2 ± 0.7	1.0	0.36 ± 0.02
4.8	31 ± 2	17.9 ± 0.4	13.4 ± 0.1	1.6	0.51 ± 0.01
7.2	45 ± 2	25 ± 1	18 ± 1	2.4	0.78 ± 0.04
9.6	58 ± 4	37 ± 2	29 ± 1	3.1	1.14 ± 0.03
12	65 ± 8	40 ± 6	31 ± 4	3.5	1.41 ± 0.04

[a]Loading of **QS-TEG$_{lock}$** on IO-ITO electrodes. [b, c, d]Photocurrent density at several time delays of IO-ITO|**QS-TEG$_{lock}$** registered by chronoamperometries at 1.12 V vs RHE in 0.1 M $NaHCO_3$, pH 7, with solar simulator equipped with AM 1.5 G filter, 850 mW cm^{-2}, λ > 450 nm. [e]Photocurrent density normalized with J_0 as lower loading sample performance (2.4 nmol cm^{-2}). [f]Diffuse reflectance intensity at 500 nm of freshly deposited photoanodes converted in KM units. Samples prepared via co-deposition of **QS-TEG$_{lock}$** on IO-ITO electrodes. Every loading is tested with 3 different samples and the value here reported is the mean value.

Table S8. Photocurrent density at several time delays and normalized J/J_0 (scaled with respect to the minimum value, Jo) response of IO-ITO|**QS** as a function of quantasome loading registered by chronoamperometries at 1.12 V vs RHE and corresponding diffuse reflectance intensity converted in KM units F(R) values at 500 nm.

**QS Loading (nmol cm^{-2})[a]**	**J @ 1.12 V vs RHE [t=10s] (μA cm^{-2})[b]**	**J @ 1.12 V vs RHE [t=110s] (μA cm^{-2})[c]**	**J @ 1.12 V vs RHE [t=300s] (μA cm^{-2})[d]**	**J/J_0 [t=10s] @ 1.12 V vs RHE[e]**	**$F(R)_0$ (KM units)[f]**
2.4	12 ± 1	7 ± 1	5.0 ± 0.4	1.0	0.49 ± 0.01
4.8	16 ± 3	9 ± 1	7 ± 1	1.3	0.67 ± 0.01
7.2	24 ± 3	12 ± 2	9 ± 2	2.0	0.90 ± 0.05
9.6	24 ± 3	14 ± 2	11 ± 2	2.0	1.11 ± 0.03
12	28 ± 2	14 ± 2	11 ± 1	2.3	1.46 ± 0.06

[a]Loading of **QS** on IO-ITO electrodes. [b, c, d]Photocurrent density at several time delays of IO-ITO|**QS** registered by chronoamperometries at 1.12 V vs RHE in 0.1 M $NaHCO_3$, pH 7, with solar simulator equipped with AM 1.5 G filter, 850 mW cm^{-2}, λ > 450 nm. [e]Photocurrent density normalized with J_0 as lower loading sample performance (2.4 nmol cm^{-2}). [f]Diffuse reflectance intensity at 500 nm of freshly deposited photoanodes converted in KM units. Samples prepared via co-deposition of **QS** on IO-ITO electrodes. Every loading is tested with 3 different samples and the value here reported is the mean value.

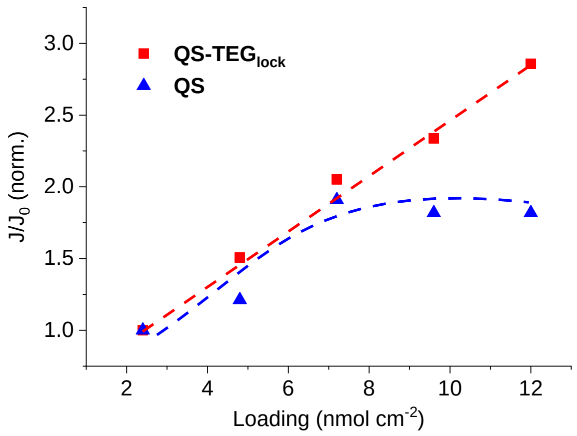

Figure S22. Normalized J/J$_o$ (scaled with respect to the minimum value, Jo) plots (mean value of three experiments) for IO-ITO|**QS** (blue triangles) and IO-ITO|**QS-TEG$_{lock}$** (red squares) at increasing loading (2.4-12 nmol/cm^2). Values are obtained from chronoamperometries (t=10 s) registered at 1.52 V vs RHE in 0.1 M $NaHCO_3$ solution, pH 7, with solar simulator equipped with AM 1.5 G filter, 850 mW cm^{-2}, $\lambda > 450$ nm.

Table S9. Photocurrent density at several time delays and normalized J/J$_o$ (scaled with respect to the minimum value, Jo) response of IO-ITO|**QS-TEG$_{lock}$** as a function of quantasome loading registered by chronoamperometries at 1.52 V vs RHE.

QS-TEG$_{lock}$ Loading (nmol cm^{-2})[a]	J @ 1.52 V vs RHE [t=10s] (μA cm^{-2})[b]	J @ 1.52 V vs RHE [t=110s] (μA cm^{-2})[c]	J @ 1.52 V vs RHE [t=300s] (μA cm^{-2})[d]	J/J$_0$ [t=10s] @ 1.52 V vs RHE[e]
2.4	77 ± 5	64 ± 5	51 ± 4	1.0
4.8	116 ± 4	84 ± 5	70 ± 6	1.5
7.2	158 ± 6	126 ± 4	109 ± 2	2.1
9.6	180 ± 10	135 ± 9	111 ± 9	2.3
12	220 ± 20	180 ± 10	130 ± 10	2.9

[a]Loading of **QS-TEG$_{lock}$** on IO-ITO electrodes. [b, c, d]Photocurrent density at several time delays of IO-ITO|**QS-TEG$_{lock}$** registered by chronoamperometries at 1.52 V vs RHE in 0.1 M $NaHCO_3$, pH 7, with solar simulator equipped with AM 1.5 G filter, 850 mW cm^{-2}, $\lambda > 450$ nm. [e]Photocurrent density normalized with J$_0$ as lower loading sample performance (2.4 nmol cm^{-2}). Samples prepared via co-deposition of **QS-TEG$_{lock}$** on IO-ITO electrodes. Every loading is tested with 3 different samples and the value here reported is the mean value.

Table S10. Photocurrent density at several time delays and normalized J/J$_o$ (scaled with respect to the minimum value, Jo) response of IO-ITO|**QS** as a function of quantasome loading registered by chronoamperometries at 1.52 V vs RHE.

QS Loading (nmol cm^{-2})[a]	J @ 1.52 V vs RHE [t=10s] (μA cm^{-2})[b]	J @ 1.52 V vs RHE [t=110s] (μA cm^{-2})[c]	J @ 1.52 V vs RHE [t=300s] (μA cm^{-2})[d]	J/J$_0$ [t=10s] @ 1.52 V vs RHE[e]
2.4	99 ± 9	67 ± 4	38 ± 2	1.0
4.8	120 ± 10	95 ± 2	64 ± 4	1.2
7.2	189 ± 7	130 ± 6	80 ± 10	1.9
9.6	180 ± 30	140 ± 20	110 ± 20	1.8
12	180 ± 10	135 ± 7	112 ± 2	1.8

[a]Loading of **QS** on IO-ITO electrodes. [b, c, d]Photocurrent density at several time delays of IO-ITO|**QS** registered by chronoamperometries at 1.52 V vs RHE in 0.1 M $NaHCO_3$, pH 7, with solar simulator equipped with AM 1.5 G filter, 850 mW cm^{-2}, $\lambda > 450$ nm. [e]Photocurrent density normalized with J$_0$ as lower loading sample performance (2.4 nmol cm^{-2}). Samples prepared via co-deposition of **QS** on IO-ITO electrodes. Every loading is tested with 3 different samples and the value here reported is the mean value.

3.18 Light management

Table S11. Photocurrent density at several time delays and normalized J/J_0 (scaled with respect to the minimum value, Jo) response of IO-ITO|**QS-TEG$_{lock}$** as a function of incident light intensity registered by chronoamperometries at 1.12 V vs RHE.

Light Intensity [mW/cm^2][a]	J @ 1.12 V vs RHE [t=10s] (μA cm^{-2})[b]	J @ 1.12 V vs RHE [t=110s] (μA cm^{-2})[c]	J @ 1.12 V vs RHE [t=300s] (μA cm^{-2})[d]	J/J_0 [t=10s] @ 1.12 V vs RHE[e]
100	20 ± 1	12,1 ± 0,9	10,1 ± 0,6	1.0
200	28 ± 4	18 ± 3	15 ± 2	1.4
300	35 ± 1	23,1	18,3	1.8
400	41 ± 3	27 ± 1	22 ± 1	2.1
670	65 ± 3	40 ± 1	31 ± 1	3.3
850	65 ± 8	40 ± 6	31 ± 4	3.3

[a]Incident light intensity. [b, c, d]Photocurrent density at several time delays of IO-ITO|**QS-TEG$_{lock}$** registered by chronoamperometries at 1.12 V vs RHE in 0.1 M $NaHCO_3$, pH 7, with solar simulator equipped with AM 1.5 G filter, λ > 450 nm. [e]Photocurrent density normalized with J_0 as performance at 100 mW/cm^2. Samples prepared via co-deposition of 12 nmol cm^{-2} of **QS-TEG$_{lock}$** on IO-ITO electrodes. Every light intensity is tested with 3 different samples and the value here reported is the mean value.

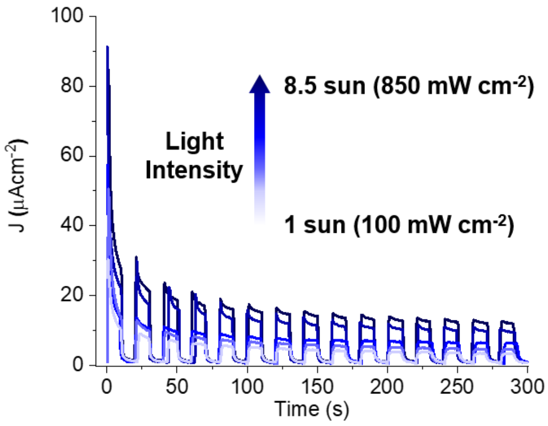

Figure S23. Representative chopped light chronoamperometry transients of IO-ITO|**QS** (12 nmol cm^{-2} loading) performed at different light intensity (I_{ph} = 100 - 850 mW cm^{-2}) and registered at 1.12 V vs RHE in 0.1 M $NaHCO_3$ solution, pH 7, with a solar simulator equipped with AM 1.5G filter, λ > 450nm (10 s light and 10 s dark).

Table S12. Photocurrent density at several time delays and normalized J/J_0 (scaled with respect to the minimum value, Jo) response of IO-ITO|**QS** as a function of incident light intensity registered by chronoamperometries at 1.12 V vs RHE.

Light Intensity [mW/cm^2][a]	J @ 1.12 V vs RHE [t=10s] (μA cm^{-2})[b]	J @ 1.12 V vs RHE [t=110s] (μA cm^{-2})[c]	J @ 1.12 V vs RHE [t=300s] (μA cm^{-2})[d]	J/J_0 [t=10s] @ 1.12 V vs RHE[e]
100	9.5 ± 0.2	4,2 ± 0,1	2,8 ± 0,2	1.0
200	12.4 ± 0.7	5,7 ± 0,4	3,9 ± 0,3	1.3
300	15 ± 2	7 ± 1	5,5 ± 0,9	1.6
400	20.6 ± 0.7	12,0 ± 0,3	9,0 ± 0,3	2.2
670	30 ± 2	17 ± 2	13 ± 2	2.9
850	28 ± 2	14 ± 2	11 ± 1	2.9

[a]Incident light intensity. [b, c, d]Photocurrent density at several time delays of IO-ITO|**QS-TEG$_{lock}$** registered by chronoamperometries at 1.12 V vs RHE in 0.1 M $NaHCO_3$, pH 7, with solar simulator equipped with AM 1.5 G filter, λ > 450 nm. [e]Photocurrent density normalized with J_0 as performance at 100 mW/cm^2. Samples prepared via co-deposition of 12 nmol cm^{-2} of **QS** on IO-ITO electrodes. Every light intensity is tested with 3 different samples and the value here reported is the mean value.

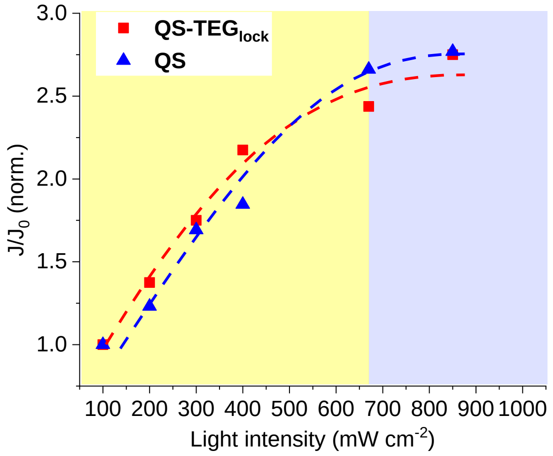

Figure S24. Normalized J/Jo (scaled with respect to the minimum value, Jo) plots (mean value of three experiments) for IO-ITO|**QS** (blue triangles) and IO-ITO|**QS-TEG$_{lock}$** (red squares) at increasing light intensity (100 - 850 mW cm^{-2}). Values are obtained from chronoamperometries (t=10 s) registered at 1.52 V vs RHE in 0.1 M $NaHCO_3$ solution, pH 7, with a solar simulator equipped with AM 1.5G filter, $\lambda > 450$ nm.

A) B)

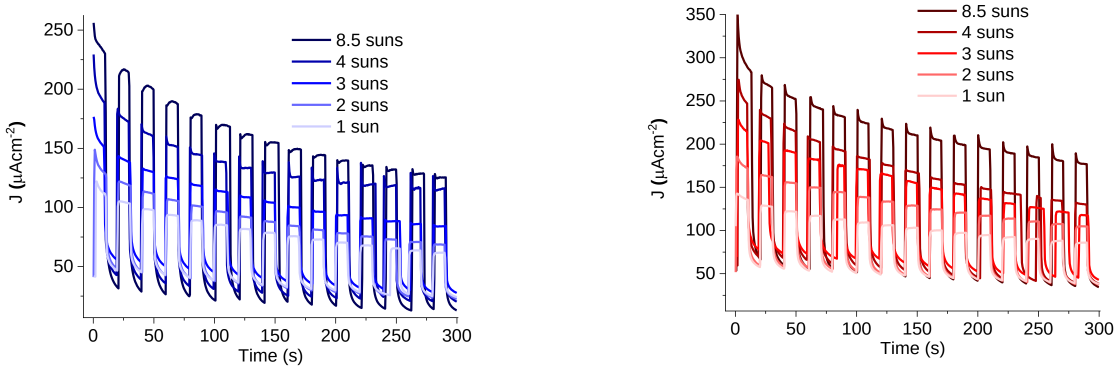

Figure S25. Representative chopped light chronoamperometries of A) IO-ITO|**QS-TEG$_{lock}$** and B) IO-ITO|**QS** (12 nmol cm^{-2} loading) performed at different light intensity (I_{ph} = 100 - 850 mW cm^{-2}) and registered at 1.52 V vs RHE in 0.1 M $NaHCO_3$ solution, pH 7, with a solar simulator equipped with AM 1.5G filter, $\lambda > 450$ nm (10 s light and 10 s dark).

Table S13. Photocurrent density at several time delays and normalized J/J$_0$ (scaled with respect to the minimum value, Jo) response of IO-ITO|**QS-TEG$_{lock}$** as a function of incident light intensity registered by chronoamperometries at 1.52 V vs RHE.

Light Intensity [mW/cm^2][a]	J @ 1.52 V vs RHE [t=10s] (µA cm^{-2})[b]	J @ 1.52 V vs RHE [t=110s] (µA cm^{-2})[c]	J @ 1.52 V vs RHE [t=300s] (µA cm^{-2})[d]	J/J$_0$ [t=10s] @1.52 V vs RHE[e]
100	80 ± 2	62 ± 2	49 ± 1	1.0
200	110 ± 20	90 ± 10	67 ± 6	1.4
300	140 ± 10	98 ± 9	72 ± 7	1.8
400	174 ± 3	126 ± 1	91 ± 1	2.2
670	195 ± 6	150 ± 10	120 ± 10	2.4
850	220 ± 20	170 ± 10	130 ± 10	2.8

[a]Incident light intensity. [b, c, d]Photocurrent density at several time delays of IO-ITO|**QS-TEG$_{lock}$** registered by chronoamperometries at 1.52 V vs RHE in 0.1 M $NaHCO_3$, pH 7, with solar simulator equipped with AM 1.5 G filter, $\lambda > 450$ nm. [e]Photocurrent density normalized with J$_0$ as performance at 100 mW/cm^2. Samples prepared via co-deposition of 12 nmol cm^{-2} of **QS-TEG$_{lock}$** on IO-ITO electrodes. Every light intensity is tested with 3 different samples and the value here reported is the mean value.

Table S14. Photocurrent density at several time delays and normalized J/J_0 (scaled with respect to the minimum value, Jo) response of IO-ITO|**QS** as a function of incident light intensity registered by chronoamperometries at 1.52 V vs RHE.

Light Intensity [mW/cm^2][a]	J @ 1.52 V vs RHE [t=10s] (μA cm^{-2})[b]	J @ 1.52 V vs RHE [t=110s] (μA cm^{-2})[c]	J @ 1.52 V vs RHE [t=300s] (μA cm^{-2})[d]	J/J_0 [t=10s] @1.52 V vs RHE[c]
100	65 ± 1	49 ± 1	37 ± 1	1.0
200	80 ± 10	61 ± 8	46 ± 5	1.2
300	110 ± 20	80 ± 10	61 ± 9	1.7
400	120 ± 20	100 ± 10	90 ± 6	1.8
670	173 ± 6	138 ± 7	120 ± 4	2.7
850	180 ± 10	135 ± 7	112 ± 2	2.8

[a]Incident light intensity. [b, c, d]Photocurrent density at several time delays of IO-ITO|**QS** registered by chronoamperometries at 1.52 V vs RHE in 0.1 M $NaHCO_3$, pH 7, with solar simulator equipped with AM 1.5 G filter, λ > 450 nm. [e]Photocurrent density normalized with J_0 as performance at 100 mW/cm^2. Samples prepared via co-deposition of 12 nmol cm^{-2} of **QS** on IO-ITO electrodes. Every light intensity is tested with 3 different samples and the value here reported is the mean value.

3.19 Synthesis and PEC performance of QS-TEG$_{unlock}$

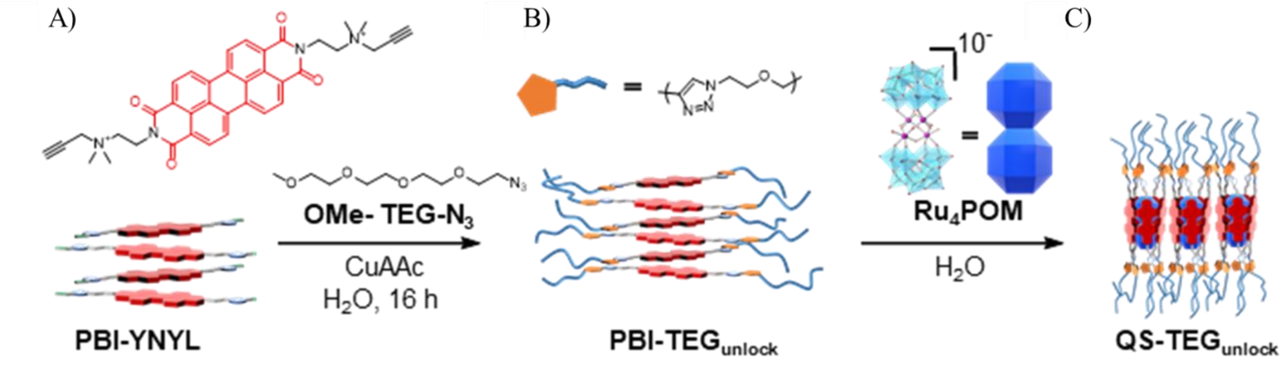

Figure S26. A) Molecular structure of **PBI-YNYL** and B) synthetic procedure to obtain **PBI-TEG$_{unlock}$**. C). self-assembly of **QS-TEG$_{unlock}$**.

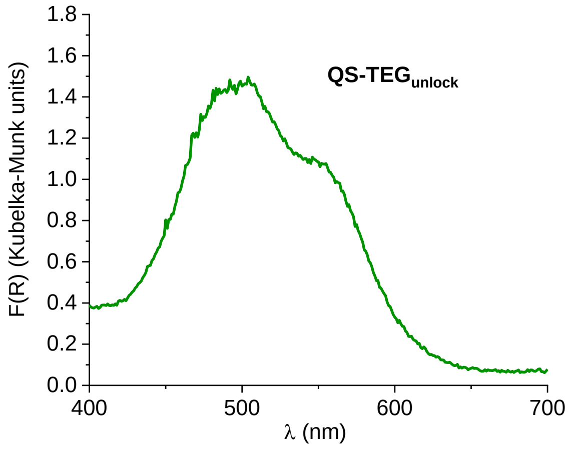

Figure S27. Diffuse reflectance spectra converted in Kubelka Munk units of IO-ITO|**QS-TEG$_{unlock}$** (12 nmol cm^{-2} loading).

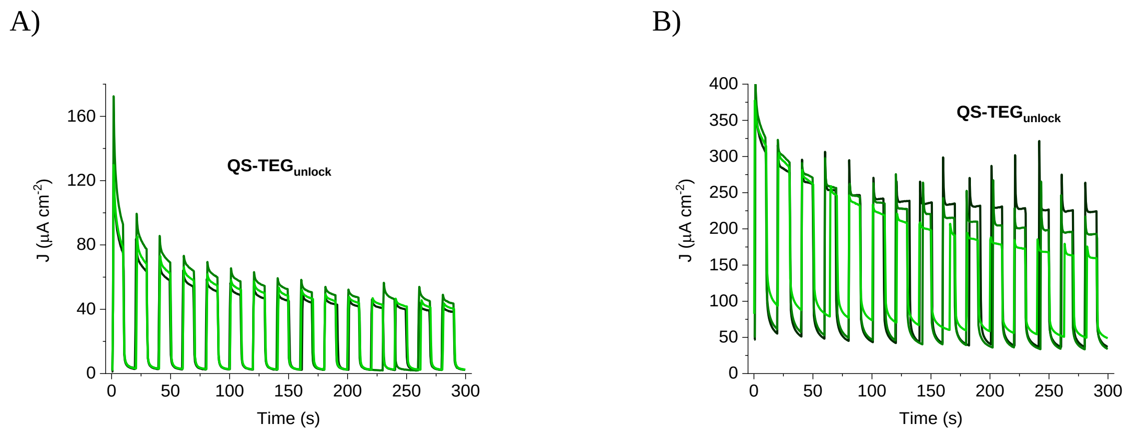

Figure S28. Chopped light chronoamperometries of IO-ITO|**QS-TEG$_{unlock}$** (12 nmol cm^{-2} loading) registered at A) 1.12 V vs RHE and B) 1.52 V vs RHE in 0.1 M $NaHCO_3$ solution, pH 7, with a solar simulator equipped with AM 1.5G filter, 850 mW cm^{-2}, $\lambda > 450$ nm (10 s light and 10 s dark).

Table S15. Photocurrent density at several time delays of IO-ITO|**QS-TEG$_{unlock}$** registered by chronoamperometries at 1.12 V and 1.52 V vs RHE.

**E (V) vs RHE**[a]	**J [t=10s] (μA cm^{-2})**[b]	**J [t=110s] (μA cm^{-2})**[c]	**J [t=300s] (μA cm^{-2})**[d]
1.12	79 ± 8	53 ± 3	41 ± 2
1.52	250 ± 20	170 ± 20	140 ± 30

[a]Potential applied. [b, c, d]Photocurrent density at several time delays of IO-ITO|**QS-TEG$_{unlock}$** registered by chronoamperometries at 1.12 V and 1.52 vs RHE in 0.1 M $NaHCO_3$, pH 7, with solar simulator equipped with AM 1.5 G filter, 850 mW cm^{-2}, $\lambda > 450$ nm. Samples prepared via co-deposition of 12 nmol cm^{-2} of **QS-TEG$_{unlock}$** on IO-ITO electrodes. Every measure is repeated 3 times and the value here reported is the mean value.

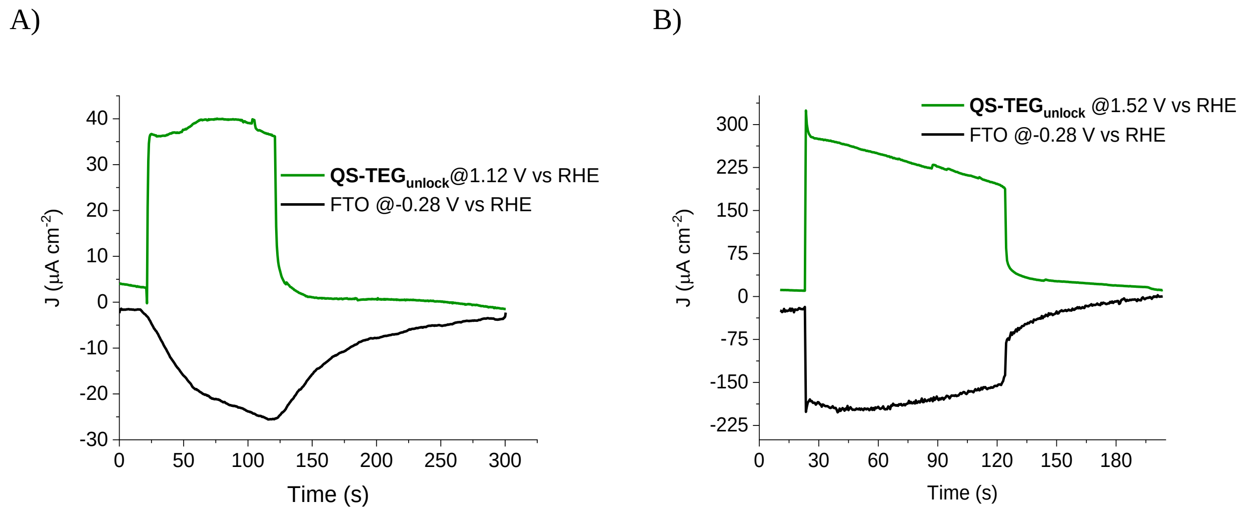

Figure S29. Photocurrent traces of IO-ITO|**QS-TEG$_{unlock}$** (green trace) (12 nmol cm^{-2} loading) registered at A) 1.12 V vs RHE and B) 1.52 V vs RHE applied bias in 0.1 M $NaHCO_3$, pH 7, with solar simulator equipped with AM 1.5 G filter, 850 mW cm^{-2}, $\lambda > 450$ nm, and corresponding O_2 reduction trace registered at the FTO collector under -0.28 V vs RHE applied bias (black trace).

Table S16. FE_{O2}, determined with generator – collector method,[1,8–10] of IO-ITO|**QS-TEG**$_{\textbf{unlock}}$ prepared via co-deposition of **QS-TEG**$_{\textbf{unlock}}$ (12 nmol cm^{-2}) on IO-ITO electrodes.

Photoanode	E (V) vs RHE[a]	Faradaic Efficiency (%)[b]	E (V) vs RHE[c]	Faradaic Efficiency (%)[d]
IO-ITO|**QS-TEG**$_{\textbf{unlock}}$	1.12	100 ± 10	1.52	99 ± 3

[a,c] Voltage applied at generator electrode reported vs RHE. [b,d] Faradaic efficiency measured with a collection efficiency of 78%. Experiments are registered in 0.1 M $NaHCO_3$, pH 7 with solar simulator equipped with AM 1.5 G filter, 850 mW cm^{-2}, λ >450 nm. Every measure is repeated at least 3 times and the value here reported is the mean value.

3.20 Determination of surface-active sites of Ru$_4$POM used for calculation of the dark electrocatalytic TOF

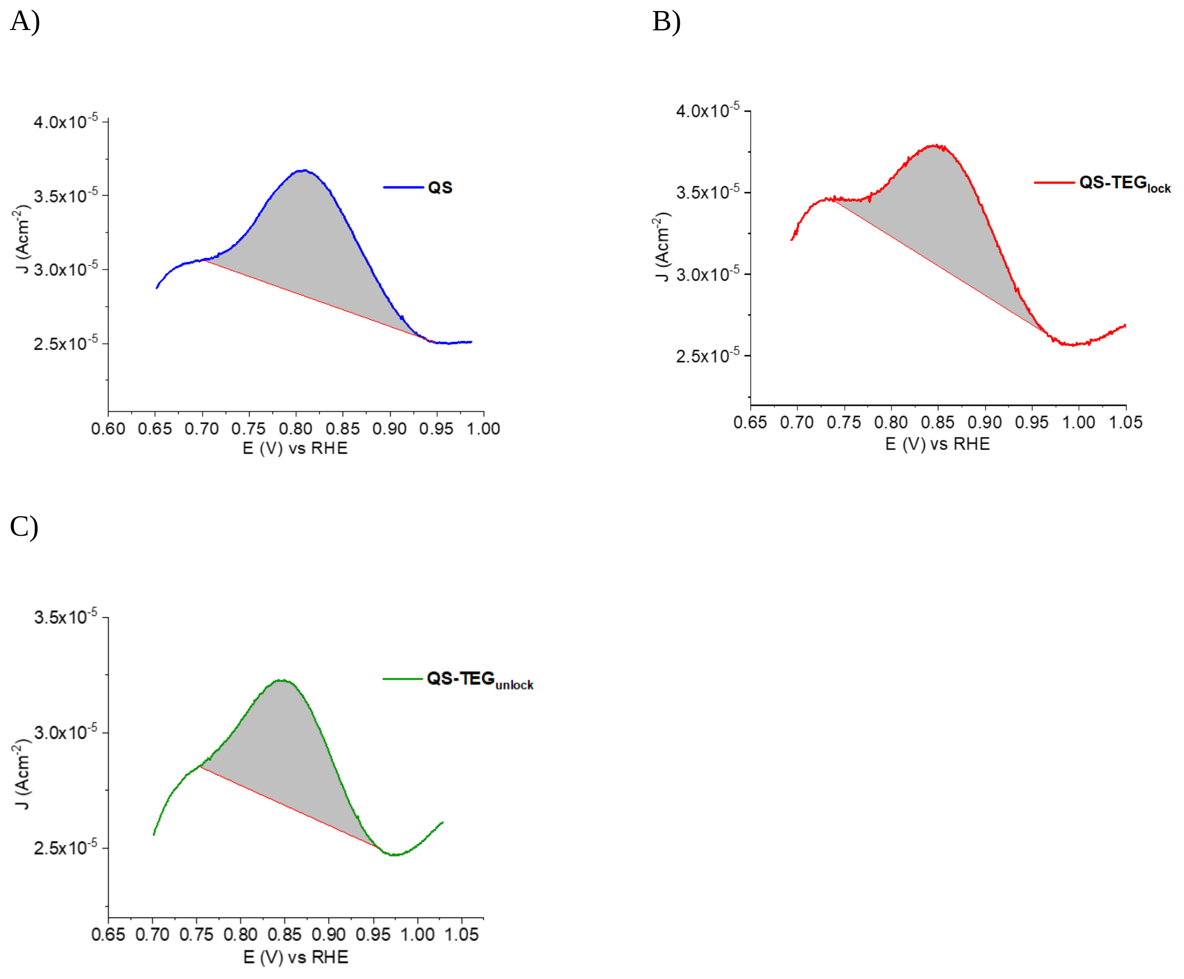

Figure S30. Area of the oxidation peak of **Ru$_4$POM** on A) IO-ITO|**QS**, B) IO-ITO|**QS-TEG**$_{\textbf{lock}}$ and C) IO-ITO|**QS-TEG**$_{\textbf{unlock}}$. (12 nmol cm^{-2} loading) .

Table S17. Calculation of active sites of **Ru$_4$POM** on IO-ITO|**QS,** IO-ITO|**QS-TEG**$_{\textbf{lock}}$ and ITO|**QS-TEG**$_{\textbf{unlock}}$. prepared via co-deposition of **QS** (12 nmol cm^{-2}), **QS-TEG**$_{\textbf{lock}}$ (12 nmol cm^{-2}) and **QS-TEG**$_{\textbf{unlock}}$ (12 nmol cm^{-2}) on IO-ITO electrodes.

Photoanode	E (V) vs RHE[a]	Calculated Area(AV/cm^2)[b]	Charge(As/cm^2)[c]	Active sites(nmol/cm^2)[d]
IO-ITO|**QS**	0.81 ± 0.1	(1.1± 0.2)10^{-6}	(1.1 ± 0.2)10^{-4}	1.2 ± 0.2
IO-ITO|**QS-TEG**$_{\textbf{lock}}$	0.83 ± 0.2	(1.0 ± 0.2)10^{-6}	(1 ± 0.2)10^{-4}	1.0 ± 0.2
IO-ITO|**QS-TEG**$_{\textbf{unlock}}$	0.83 ± 0.2	(9 ± 3)10^{-7}	(9 ± 3)10^{-5}	0.9 ± 0.3

[a] Oxidation peak potential, [b]calculated area and [c]charge extrapolated from the oxidation redox active peak associated to a monoelectronic process as reported in Pourbaix diagram from literature;[15] [d]Active sites of **Ru$_4$POM** deposited on IO-ITO|**QS,** IO-ITO|**QS-TEG**$_{\textbf{lock}}$ and IO-ITO|**QS-TEG**$_{\textbf{unlock}}$. Every parameter is obtained from the mean value of at least 3 different samples.

3.21 Dark electrocatalytic water oxidation on IO-ITO|QS, IO-ITO|QS-TEG$_{lock}$ and IO-ITO|QS-TEG$_{unlock}$

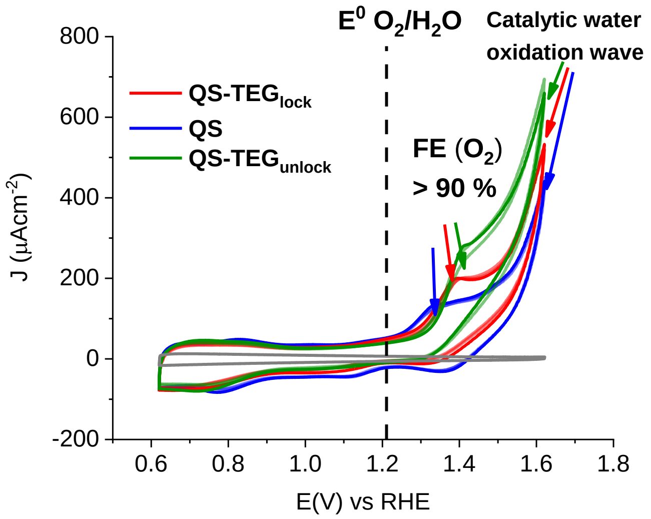

Figure S31. Representative cyclic voltammetry responses IO-ITO|**QS** (blue traces), IO-ITO|**QS-TEG$_{lock}$** (red traces), IO-ITO|**QS-TEG$_{unlock}$** (green traces) (12 nmol cm^{-2} loading) superimposed to bare IO-ITO (grey trace) registered in 0.1 M $NaHCO_3$ solution, pH 7, scan rate 10 mV/s.

Table S18. Electrocatalytic performances of IO-ITO|**QS**, IO-ITO|**QS-TEG$_{lock}$** and IO-ITO|**QS-TEG$_{unlock}$** prepared via co-deposition of **QS** (12 nmol cm^{-2}), **QS-TEG$_{lock}$** (12 nmol cm^{-2}) and **QS-TEG$_{unlock}$** (12 nmol cm^{-2}) on IO-ITO electrodes.

Photoanode	Onset Potential (vs RHE)[a]	Peak Potential (vs RHE)[b]	Peak Current (µA cm^{-2})[c]	Current at 1.62 V vs RHE (µA cm^{-2})[d]	TOF (1/s)[e]
**IO-ITO|QS**	1.25 ±0.03	1.35 ± 0.04	120 ± 20	300 ± 100	0.94 ± 0.03
**IO-ITO|QS-TEG$_{lock}$**	1.30 ± 0.02	1.43 ± 0.03	200 ± 20	450 ± 60	1.36 ± 0.02
**IO-ITO|QS-TEG$_{unlock}$**	1.33 ± 0.03	1.42 ± 0.03	240 ± 40	500 ± 100	1.92 ± 0.06

[a] Onset potential, [b] peak potential and [c] peak current of the first oxygenic manifold in 0.1 M $NaHCO_3$, pH 7, scan rate 10 mV/s; [d] Dark current related to water oxidation at 1.62 V vs RHE in 0.1 M $NaHCO_3$, pH 7, scan rate 10 mV/s. [e] Turnover frequency of the catalyst calculated from registered current at 1.62 V vs RHE considering the active sites measured in section 3.20. Every parameter is obtained from the mean value of at least 3 different samples.

A)

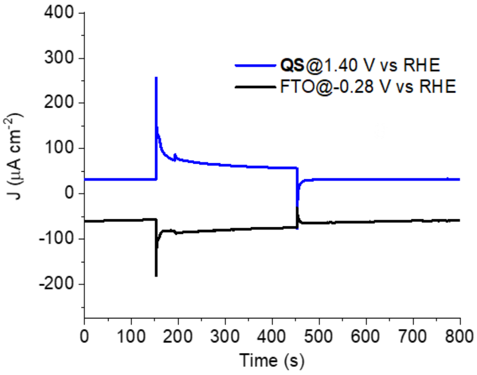

B)

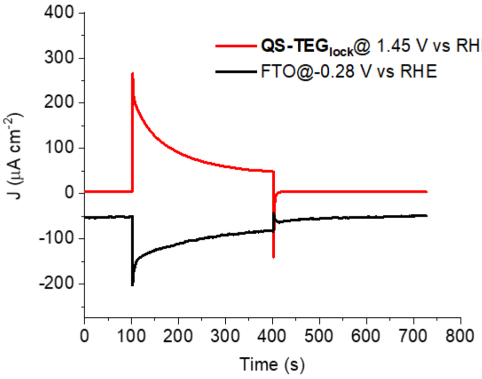

C)

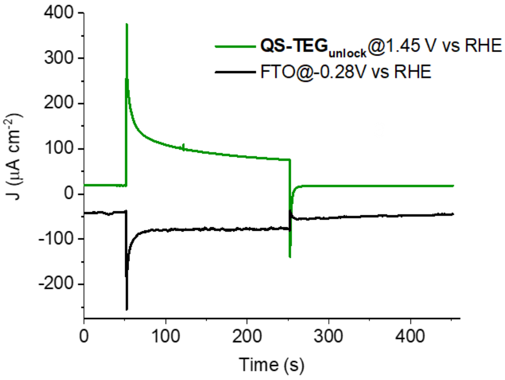

Figure S32. Generator-collector traces of A) IO-ITO|**QS** (blue trace), B) IO-ITO|**QS-TEG$_{lock}$** (red trace) and C) IO-ITO|**QS-TEG$_{unlock}$** (green trace) (12 nmol cm^{-2} loading) registered in 0.1 M $NaHCO_3$, pH 7 and corresponding O_2 reduction trace registered at the FTO collector under -0.28 V vs RHE applied bias (black trace).

Table S19. FE_{O2} under dark conditions, determined with generator – collector method,[1,8–10] of IO-ITO|**QS**, IO-ITO|**QS-TEG$_{lock}$** and IO-ITO|**QS-TEG$_{unlock}$** prepared via co-deposition of **QS** (12 nmol cm^{-2}), **QS-TEG$_{lock}$** (12 nmol cm^{-2}) and **QS-TEG$_{unlock}$** (12 nmol cm^{-2}) on IO-ITO electrodes.

Photoanode	E (V) vs RHE[a]	Faradaic Efficiency (%)[b]
**IO-ITO|QS**	1.40	100 ± 10
**IO-ITO|QS-TEG$_{lock}$**	1.45	99 ± 5
**IO-ITO|QS-TEG$_{unlock}$**	1.45	90 ± 10

[a] Voltage applied at generator electrode reported vs RHE. [b] Faradaic efficiency measured with a collection efficiency of 78%. Experiments are registered in 0.1 M $NaHCO_3$, pH 7. Every measure is repeated at least 3 times and the value here reported is the mean value.

3.22 Raman Spectroscopy Mapping of Quantasomes Hydration Shells

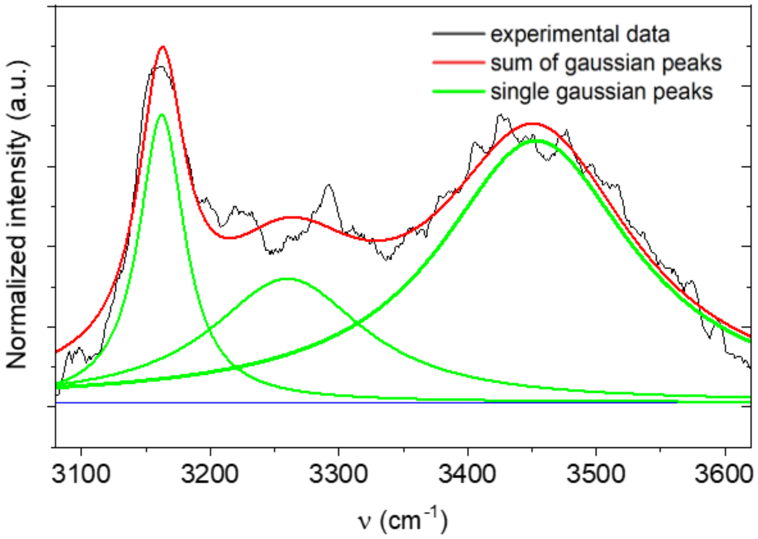

Figure S33. Raman spectroscopy measurements of IO-ITO|**QS** upon addition of 5 μL of water. Black line represents experimental smoothed data (average of 3 measurements) while the solid red line is the fit to the sum of multiple gaussian components (green lines).

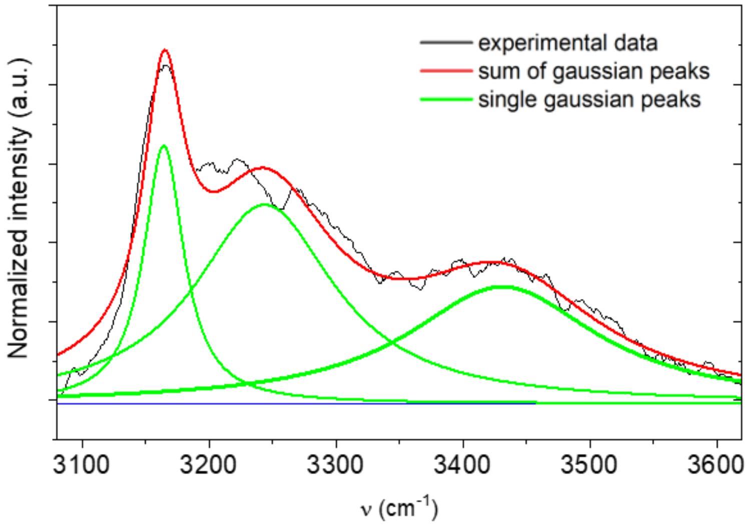

Figure S34. Raman spectroscopy measurements of IO-ITO|**QS-TEG$_{lock}$** upon addition of 5 μL of water. Black line represents experimental smoothed data (average of 3 measurements) while the solid red line is the fit to the sum of multiple gaussian components (green lines).

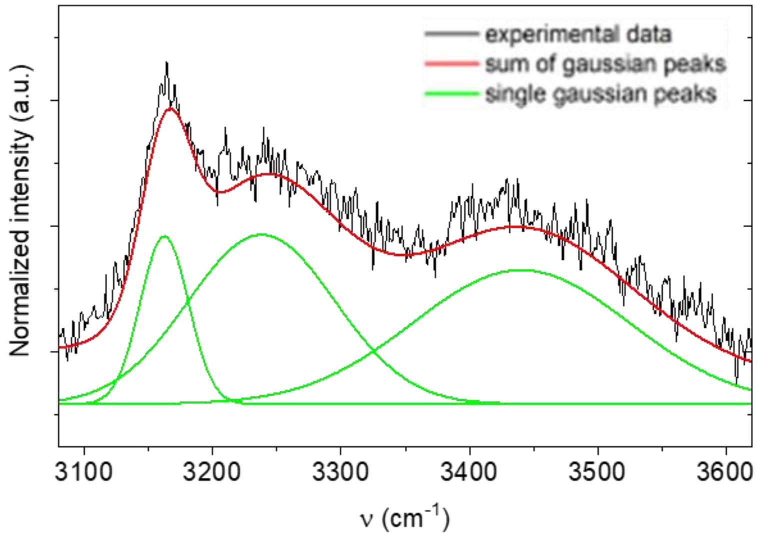

Figure S35. Raman spectroscopy measurements of IO-ITO|**QS-TEG$_{unlock}$** upon addition of 5 µL of water. Black line represents experimental smoothed data (average of 3 measurements) while the solid red line is the fit to the sum of multiple gaussian components (green lines).

3.23 Prolonged photoelectrochemical experiments

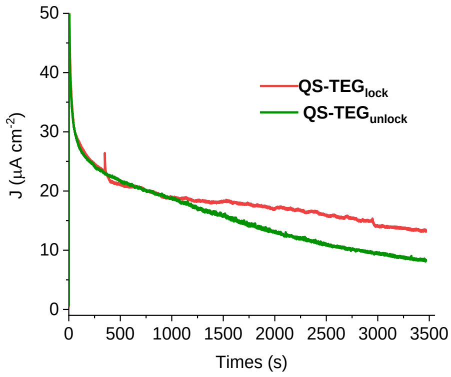

Figure S36. Prolonged photoelectrochemical response of IO – ITO|**QS-TEG$_{lock}$** (red trace) and of IO – ITO|**QS-TEG$_{unlock}$** (green trace) (12 nmol cm^{-2} loading) registered by chronoamperometry (CA) at 1.12 V vs RHE in 0.1 M $NaHCO_3$, pH 7, with solar simulator equipped with AM 1.5 G filter, 850 mW cm^{-2}, λ > 450 nm.

3.24 Stability of Photoanodes before and after oxygenic turnover

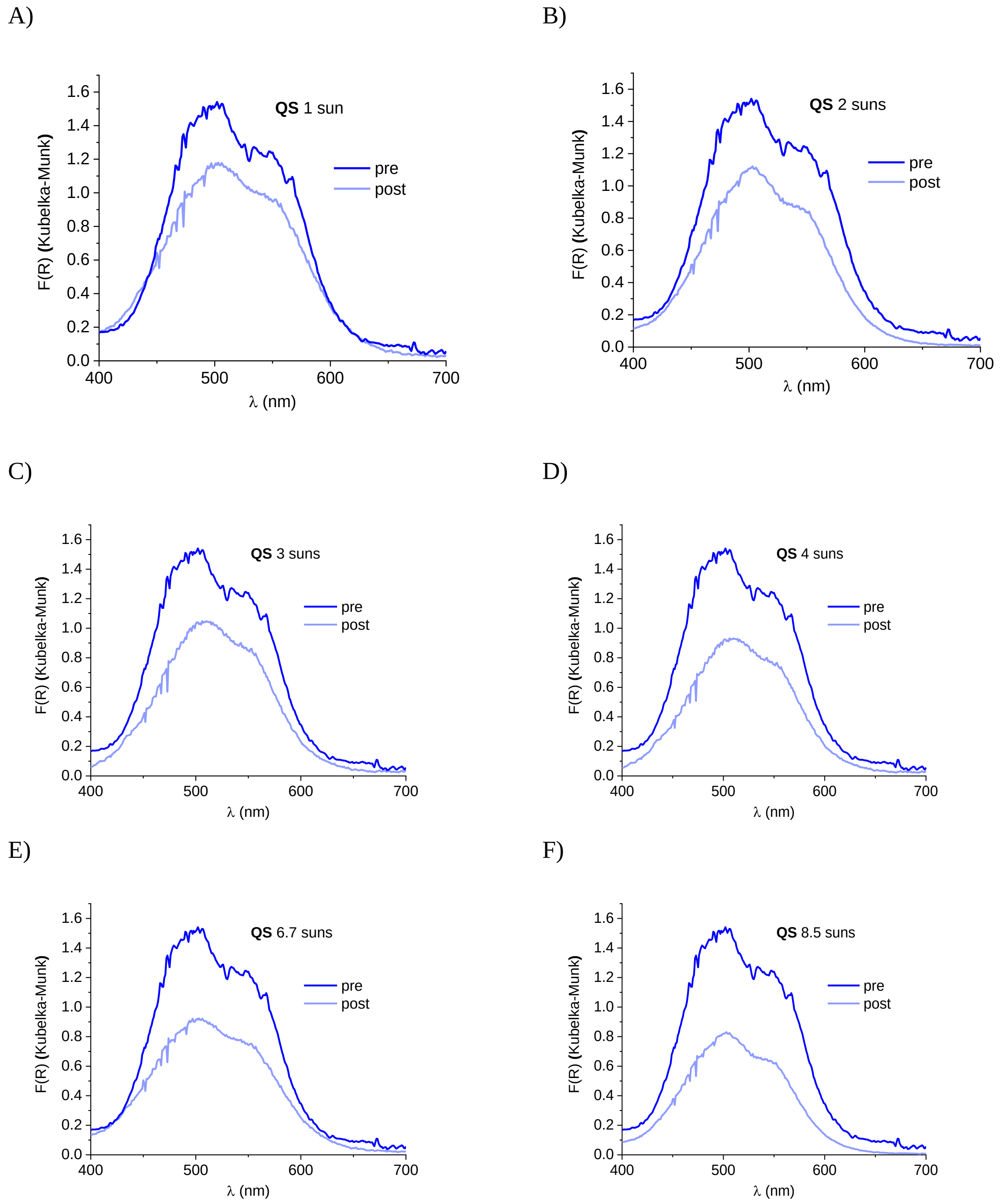

Figure S37. Diffuse reflectance spectra converted in Kubelka Munk units of IO-ITO|**QS** (12 nmol cm^{-2} loading) before and after 300 s chronoamperometry at 1.12 V and 1.52 V vs RHE recorded at light intensity equal to a) 100 mW cm^{-2}; b) 200 mW cm^{-2}; c) 300 mW cm^{-2}; d) 400 mW cm^{-2}; e) 670 mW cm^{-2} ;f) 850 mW cm^{-2}.

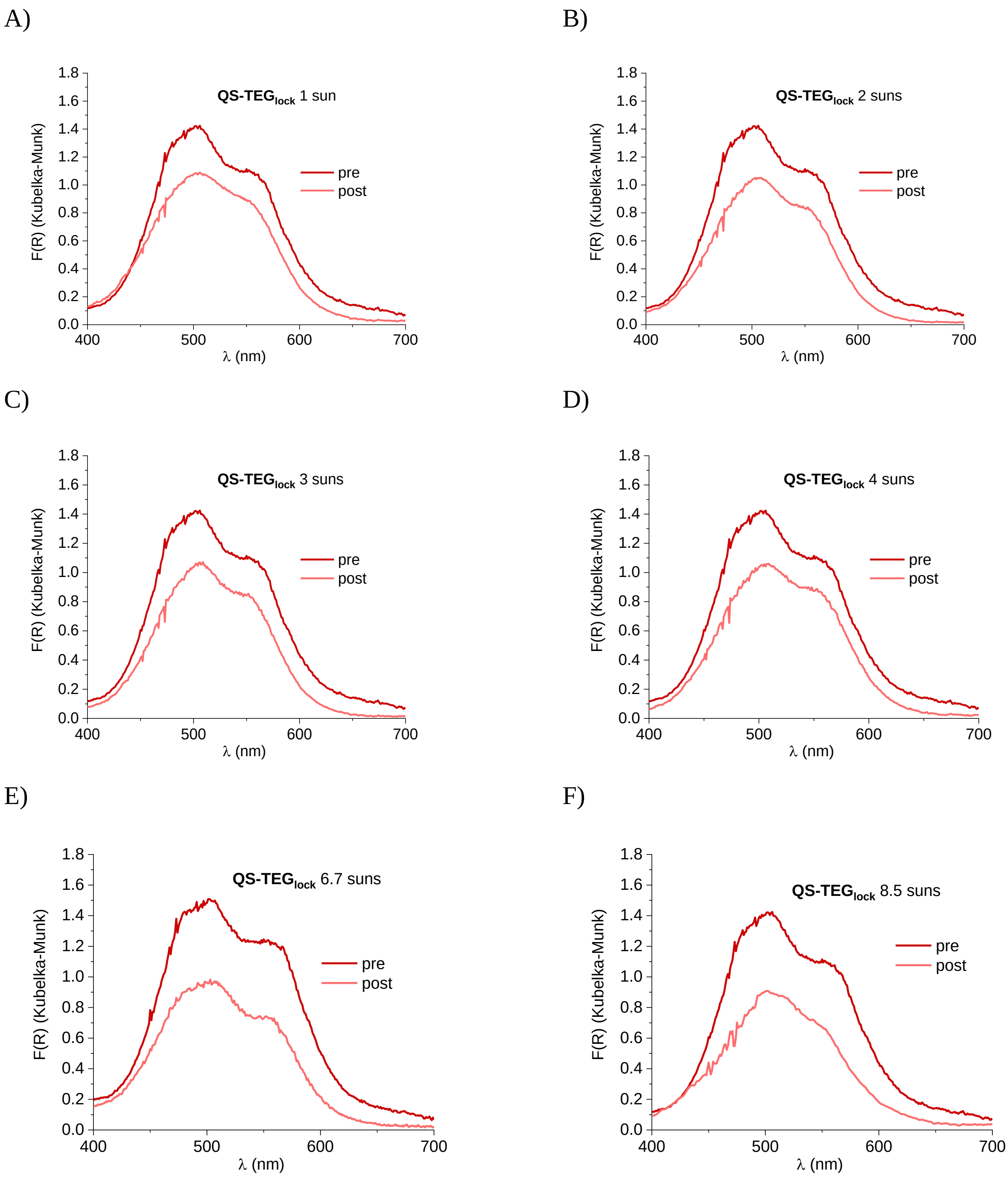

Figure S38. Diffuse reflectance spectra converted in Kubelka Munk units of IO-ITO|**QS-TEG$_{lock}$** (12 nmol cm^{-2} loading) before and after 300s chronoamperometry at 1.12 V and 1.52 V vs RHE recorded at sun power equal to a) 100 mW cm^{-2}; b) 200 mW cm^{-2}; c) 300 mW cm^{-2}; d) 400 mW cm^{-2}; e) 670 mW cm^{-2}; f) 850 mW cm^{-2}.

A) B)

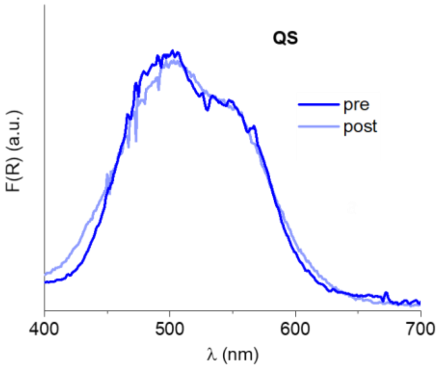

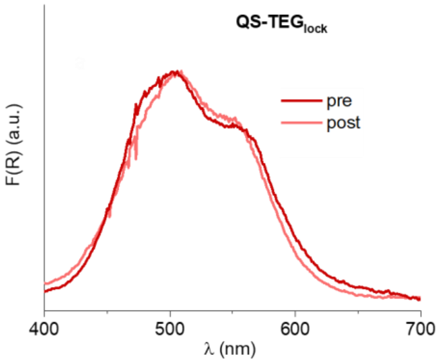

Figure S39. Superimposed diffuse reflectance spectra converted in Kubelka Munk units of (12 nmol cm^{-2} loading) A) IO-ITO|**QS** (blue traces) and B) IO-ITO|**QS-TEG$_{lock}$** (red traces) before and after 300s chronoamperometry at 1.12 V and 1.52 V vs RHE recorded at 850 mW cm^{-2}. Reflectance spectra are registered on 3 samples per each loading.

Table S20. Diffuse reflectance loss converted in Kubelka Munk units (F(R)) at 500 nm of IO-ITO|**QS** and IO-ITO|**QS-TEG$_{lock}$** after photoelectrochemical experiments at different sun power.

**Sun Power [mW/cm^2]**[a]	IO-ITO|**QS** **1 -F(R)/F(R)$_0$**[b]	IO-ITO|**QS-TEG$_{lock}$** **1 -F(R)/F(R)$_0$**[b]
100	27 %	24 %
200	29 %	25 %
300	34 %	26 %
400	36 %	32 %
670	35%	32%
850	38 %	36 %

[a]Applied sun power. [b]Loss of reflectance intensity at 500 nm converted in Kubelka Munk units after recording experiments at different light intensities (F(R) = intensity after 300 s chronoamperometries at 1.12 V and 1.52 V vs RHE; F(R)$_0$ = intensity of the photoanodes freshly deposited). Samples prepared via co-deposition of 12 nmol cm^{-2} of **QS** or **QS-TEG$_{lock}$** on IO-ITO electrodes. Every measure is repeated at least 3 times and the value here reported is the mean value.

4. Appendix

4.1 Reproducibility of QS and QS-TEG$_{lock}$ photoanodes

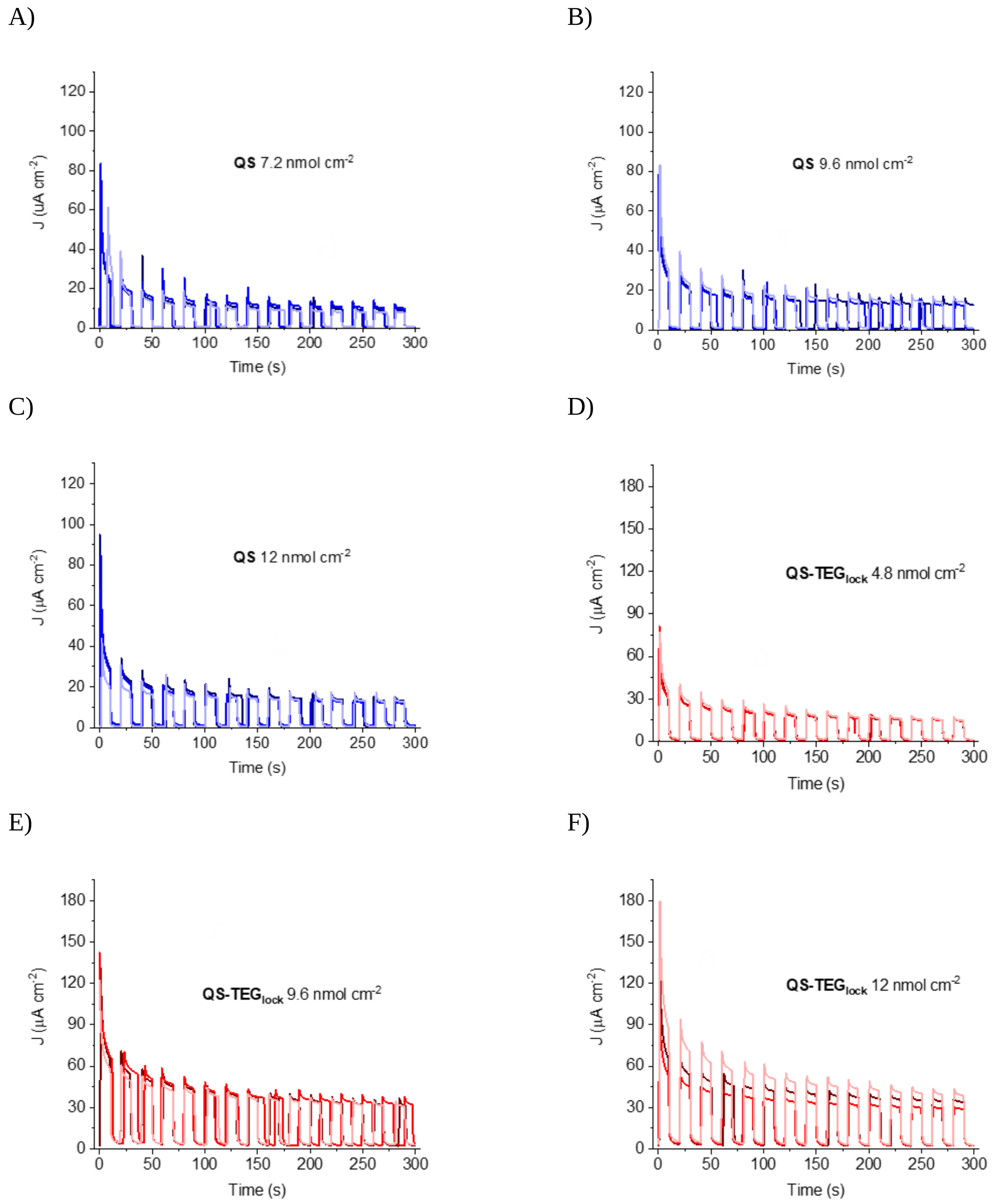

Figure S40. Chopped light chronoamperometries of IO-ITO|**QS** a) 7.2 nmol cm^{-2}, b) 9.6 nmol cm^{-2}, c) 12 nmol cm^{-2} and of IO-ITO|**QS-TEG$_{lock}$** d) 4.8 nmol cm^{-2}, e) 9.6 nmol cm^{-2}, f) 12 nmol cm^{-2} registered at 1.12 V vs RHE in 0.1 M $NaHCO_3$ solution, pH 7, with a solar simulator equipped with AM 1.5G filter, 850 mW cm^{-2}, λ > 450 nm (10 s light and 10 s dark).

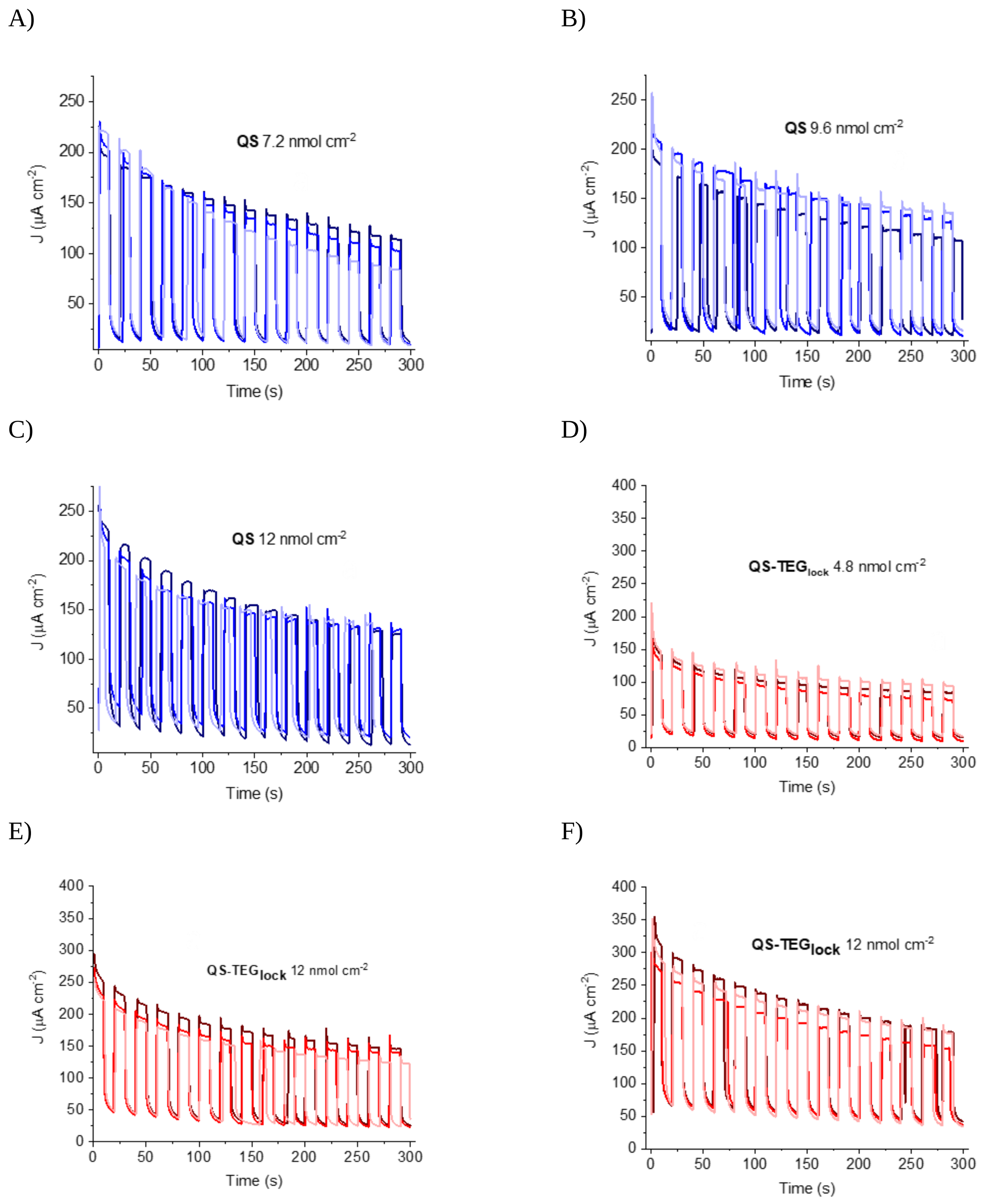

Figure S41. Chopped light chronoamperometries of IO-ITO|**QS** a) 7.2 nmol cm^{-2}, b) 9.6 nmol cm^{-2}, c) 12 nmol cm^{-2} and of IO-ITO|**QS-TEG$_{lock}$** d) 4.8 nmol cm^{-2}, e) 9.6 nmol cm^{-2}, f) 12 nmol cm^{-2} registered at 1.52 V vs RHE in 0.1 M $NaHCO_3$ solution, pH 7, with a solar simulator equipped with AM 1.5G filter, 850 mW cm^{-2}, λ > 450 nm (10 s light and 10 s dark).

5. References

(1) Bonchio, M.; Syrgiannis, Z.; Burian, M.; Marino, N.; Pizzolato, E.; Dirian, K.; Rigodanza, F.; Volpato, G. A.; La Ganga, G.; Demitri, N.; Berardi, S.; Amenitsch, H.; Guldi, D. M.; Caramori, S.; Bignozzi, C. A.; Sartorel, A.; Prato, M. Hierarchical Organization of Perylene Bisimides and Polyoxometalates for Photo-Assisted Water Oxidation. *Nat. Chem.* **2019**, *11* (2), 146–153. https://doi.org/10.1038/s41557-018-0172-y.

(2) Rigodanza, F.; Tenori, E.; Bonasera, A.; Syrgiannis, Z.; Prato, M. Fast and Efficient Microwave-Assisted Synthesis of Perylenebisimides. *European J. Org. Chem.* **2015**, *2015* (23), 5060–5063.

https://doi.org/10.1002/ejoc.201500670.

(3) Sartorel, A.; Carraro, M.; Scorrano, G.; De Zorzi, R.; Geremia, S.; McDaniel, N. D.; Bernhard, S.; Bonchio, M. Polyoxometalate Embedding of a Tetraruthenium(IV)-Oxo-Core by Template-Directed Metalation of [γ-SiW10O36] 8-: A Totally Inorganic Oxygen-Evolving Catalyst. *J. Am. Chem. Soc.* **2008**, *130* (15), 5006–5007. https://doi.org/10.1021/ja077837f.

(4) Würth, C.; Grabolle, M.; Pauli, J.; Spieles, M.; Resch-Genger, U. Relative and Absolute Determination of Fluorescence Quantum Yields of Transparent Samples. *Nat. Protoc.* **2013**, *8* (8), 1535–1550. https://doi.org/10.1038/nprot.2013.087.

(5) Connelly, N. G.; Geiger, W. E. Chemical Redox Agents for Organometallic Chemistry. *Chem. Rev.* **1996**, *96* (2), 877–910. https://doi.org/10.1021/cr940053x.

(6) Clark, R. N.; Roush, T. L. Reflectance Spectroscopy: Quantitative Analysis Techniques for Remote Sensing Applications. *J. Geophys. Res.* **1984**, *89* (B7), 6329–6340. https://doi.org/10.1029/JB089iB07p06329.

(7) Sheridan, M. V.; Wang, Y.; Wang, D.; Troian-Gautier, L.; Dares, C. J.; Sherman, B. D.; Meyer, T. J. Light-Driven Water Splitting Mediated by Photogenerated Bromine. *Angew. Chemie - Int. Ed.* **2018**, *57* (13), 3449–3453. https://doi.org/10.1002/anie.201708879.

(8) Zhao, Y.; Swierk, J. R.; Megiatto, J. D.; Sherman, B.; Youngblood, W. J.; Qin, D.; Lentz, D. M.; Moore, A. L.; Moore, T. A.; Gust, D.; Mallouk, T. E. Improving the Efficiency of Water Splitting in Dye-Sensitized Solar Cells by Using a Biomimetic Electron Transfer Mediator. *Proc. Natl. Acad. Sci. U. S. A.* **2012**, *109* (39), 15612–15616. https://doi.org/10.1073/pnas.1118339109.

(9) Sherman, B. D.; Sheridan, M. V.; Dares, C. J.; Meyer, T. J. Two Electrode Collector-Generator Method for the Detection of Electrochemically or Photoelectrochemically Produced O2. *Anal. Chem.* **2016**, *88* (14), 7076–7082. https://doi.org/10.1021/acs.analchem.6b00738.

(10) Kirner, J. T.; Finke, R. G. Sensitization of Nanocrystalline Metal Oxides with a Phosphonate-Functionalized Perylene Diimide for Photoelectrochemical Water Oxidation with a CoOx Catalyst. *ACS Appl. Mater. Interfaces* **2017**, *9* (33), 27625–27637. https://doi.org/10.1021/acsami.7b05874.

(11) Mersch, D.; Lee, C. Y.; Zhang, J. Z.; Brinkert, K.; Fontecilla-Camps, J. C.; Rutherford, A. W.; Reisner, E. Wiring of Photosystem II to Hydrogenase for Photoelectrochemical Water Splitting. *J. Am. Chem. Soc.* **2015**, *137* (26), 8541–8549. https://doi.org/10.1021/jacs.5b03737.

(12) Mascaretti, L.; Niorettini, A.; Bricchi, B. R.; Ghidelli, M.; Naldoni, A.; Caramori, S.; Li Bassi, A.; Berardi, S. Syngas Evolution from CO2 Electroreduction by Porous Au Nanostructures. *ACS Appl. Energy Mater.* **2020**, *3* (5), 4658–4668. https://doi.org/10.1021/acsaem.0c00301.

(13) Johnson, B. A.; Bhunia, A.; Ott, S. Electrocatalytic Water Oxidation by a Molecular Catalyst Incorporated into a Metal-Organic Framework Thin Film. *Dalt. Trans.* **2017**, *46* (5), 1382–1388. https://doi.org/10.1039/c6dt03718f.

(14) Anantharaj, S.; Ede, S. R.; Karthick, K.; Sam Sankar, S.; Sangeetha, K.; Karthik, P. E.; Kundu, S. Precision and Correctness in the Evaluation of Electrocatalytic Water Splitting: Revisiting Activity Parameters with a Critical Assessment. *Energy Environ. Sci.* **2018**, *11* (4), 744–771. https://doi.org/10.1039/c7ee03457a.

(15) Liu, Y.; Guo, S.-X.; M. Bond, A.; Zhang, J.; V. Geletii, Y.; L. Hill, C. Voltammetric Determination of the Reversible Potentials for [{Ru4O4(OH)2(H2O)4}(γ-SiW10O36)2]10– over the PH Range of 2–12: Electrolyte Dependence and Implications for Water Oxidation Catalysis. *Inorg. Chem.* **2013**, *52* (20), 11986–11996. https://doi.org/10.1021/ic401748y.

(16) Ashcraft, A.; Liu, K.; Mukhopadhyay, A.; Paulino, V.; Liu, C.; Bernard, B.; Husainy, D.; Phan, T.; Olivier, J. H. A Molecular Strategy to Lock-in the Conformation of a Perylene Bisimide-Derived Supramolecular Polymer. *Angew. Chemie - Int. Ed.* **2020**, *59* (19), 7487–7493. https://doi.org/10.1002/anie.201911780.

(17) Vallée, M. R. J.; Majkut, P.; Wilkening, I.; Weise, C.; Müller, G.; Hackenberger, C. P. R. Staudinger-Phosphonite Reactions for the Chemoselective Transformation of Azido-Containing Peptides and Proteins. *Org. Lett.* **2011**, *13* (20), 5440–5443. https://doi.org/10.1021/ol2020175.

(18) Sokol, K. P.; Mersch, D.; Hartmann, V.; Zhang, J. Z.; Nowaczyk, M. M.; Rögner, M.; Ruff, A.; Schuhmann, W.; Plumeré, N.; Reisner, E. Rational Wiring of Photosystem II to Hierarchical Indium Tin Oxide Electrodes Using Redox Polymers. *Energy Environ. Sci.* **2016**, *9* (12), 3698–3709. https://doi.org/10.1039/c6ee01363e.

(19) Shen, L.; Zhang, S.; Ding, H.; Niu, F.; Chu, Y.; Wu, W.; Hu, Y.; Hu, K.; Hua, J. Pure Organic Quinacridone Dyes as Dual Sensitizers in Tandem Photoelectrochemical Cells for Unassisted Total Water Splitting. *Chem. Commun.* **2021**, *57* (46), 5634–5637. https://doi.org/10.1039/d1cc01570b.

(20) Youngblood, J. W.; Lee, S. H. A.; Kobayashi, Y.; Hernandez-Pagan, E. A.; Hoertz, P. G.; Moore, T. A.; Moore, A. L.; Gust, D.; Mallouk, T. E. Photoassisted Overall Water Splitting in a Visible Light-Absorbing Dye-Sensitized Photoelectrochemical Cell. *J. Am. Chem. Soc.* **2009**, *131* (3), 926–927. https://doi.org/10.1021/ja809108y.

(21) Fielden, J.; Sumliner, J. M.; Han, N.; Geletii, Y. V.; Xiang, X.; Musaev, D. G.; Lian, T.; Hill, C. L. Water Splitting with Polyoxometalate-Treated Photoanodes: Enhancing Performance through Sensitizer Design. *Chem. Sci.* **2015**, *6* (10), 5531–5543. https://doi.org/10.1039/c5sc01439e.

(22) Wang, D.; Sampaio, R. N.; Troian-Gautier, L.; Marquard, S. L.; Farnum, B. H.; Sherman, B. D.; Sheridan, M. V.; Dares, C. J.; Meyer, G. J.; Meyer, T. J. Molecular Photoelectrode for Water Oxidation Inspired by Photosystem II. *J. Am. Chem. Soc.* **2019**, *141* (19), 7926–7933. https://doi.org/10.1021/jacs.9b02548.

(23) Kamire, R. J.; Materna, K. L.; Hoffeditz, W. L.; Phelan, B. T.; Thomsen, J. M.; Farha, O. K.; Hupp, J. T.; Brudvig, G. W.; Wasielewski, M. R. Photodriven Oxidation of Surface-Bound Iridium-Based Molecular Water-Oxidation Catalysts on Perylene-3,4-Dicarboximide-Sensitized TiO2 Electrodes Protected by an Al2O3 Layer. *J. Phys. Chem. C* **2017**, *121* (7), 3752–3764. https://doi.org/10.1021/acs.jpcc.6b11672.

(24) Zhu, Y.; Wang, D.; Huang, Q.; Du, J.; Sun, L.; Li, F.; Meyer, T. J. Stabilization of a Molecular Water Oxidation Catalyst on a Dye–sensitized Photoanode by a Pyridyl Anchor. *Nat. Commun.* **2020**, *11* (1), 1–8. https://doi.org/10.1038/s41467-020-18417-5.

(25) Kirner, J. T.; Stracke, J. J.; Gregg, B. A.; Finke, R. G. Visible-Light-Assisted Photoelectrochemical Water Oxidation by Thin Films of a Phosphonate-Functionalized Perylene Diimide plus CoOx Cocatalyst. *ACS Appl. Mater. Interfaces* **2014**, *6* (16), 13367–13377. https://doi.org/10.1021/am405598w.

(26) Wang, D.; Niu, F.; Mortelliti, M. J.; Sheridan, M. V.; Sherman, B. D.; Zhu, Y.; McBride, J. R.; Dempsey, J. L.; Shen, S.; Dares, C. J.; Li, F.; Meyer, T. J. A Stable Dye-Sensitized Photoelectrosynthesis Cell Mediated by a NiO Overlayer for Water Oxidation. *Proc. Natl. Acad. Sci. U. S. A.* **2020**, *117* (23), 12564–12571. https://doi.org/10.1073/pnas.1821687116.

(27) Chen, Z.; Stepanenko, V.; Dehm, V.; Prins, P.; Siebbeles, L. D. A.; Seibt, J.; Marquetand, P.; Engel, V.; Würthner, F. Photoluminescence and Conductivity of Self-Assembled π-π Stacks of Perylene Bisimide Dyes. *Chem. - A Eur. J.* **2007**, *13* (2), 436–449. https://doi.org/10.1002/chem.200600889.

(28) You, C. C.; Hippius, C.; Grüne, M.; Würthner, F. Light-Harvesting Metallosupramolecular Squares Composed of Perylene Bisimide Walls and Fluorescent Antenna Dyes. *Chem. - A Eur. J.* **2006**, *12* (28), 7510–7519. https://doi.org/10.1002/chem.200600413.

(29) Shao, C.; Grüne, M.; Stolte, M.; Würthner, F. Perylene Bisimide Dimer Aggregates: Fundamental Insights into Self-Assembly by NMR and UV/Vis Spectroscopy. *Chem. - A Eur. J.* **2012**, *18* (43), 13665–13677. https://doi.org/10.1002/chem.201201661.

(30) Shao, C.; Stolte, M.; Würthner, F. Backbone-Directed Perylene Dye Self-Assembly into Oligomer Stacks. *Angew. Chemie - Int. Ed.* **2013**, *52* (40), 10463–10467. https://doi.org/10.1002/anie.201305894.

(31) Burian, M.; Rigodanza, F.; Amenitsch, H.; Almásy, L.; Khalakhan, I.; Syrgiannis, Z.; Prato, M. Structural and Optical Properties of a Perylene Bisimide in Aqueous Media. *Chem. Phys. Lett.* **2017**, *683*, 454–458. https://doi.org/10.1016/j.cplett.2017.03.056.

(32) Ashford, D. L.; Brennaman, M. K.; Brown, R. J.; Keinan, S.; Concepcion, J. J.; Papanikolas, J. M.; Templeton, J. L.; Meyer, T. J. Varying the Electronic Structure of Surface-Bound Ruthenium(II) Polypyridyl Complexes. *Inorg. Chem.* **2015**, *54* (2), 460–469. https://doi.org/10.1021/ic501682k.

(33) McCrory, C. C. L.; Jung, S.; Ferrer, I. M.; Chatman, S. M.; Peters, J. C.; Jaramillo, T. F. Benchmarking Hydrogen Evolving Reaction and Oxygen Evolving Reaction Electrocatalysts for Solar Water Splitting Devices. *J. Am. Chem. Soc.* **2015**, *137* (13), 4347–4357. https://doi.org/10.1021/ja510442p.
